# Evolution of visual guanylyl cyclases and their activating proteins with respect to clade and species-specific visual system adaptation

**DOI:** 10.3389/fnmol.2023.1131093

**Published:** 2023-03-16

**Authors:** Matthias Gesemann, Stephan C. F. Neuhauss

**Affiliations:** Department of Molecular Life Sciences, University of Zurich, Zurich, Switzerland

**Keywords:** guanylyl cyclase activating protein (GCAP), evolution, opsin, blind, visual regression, photoreceptor, nocturnal, crepuscular

## Abstract

Membrane guanylyl cyclase receptors are important regulators of local cGMP production, critically influencing cell growth and differentiation as well as ion transport, blood pressure and calcium feedback of vertebrate phototransduction. Currently, seven different subtypes of membrane guanylyl cyclase receptors have been characterized. These receptors have tissue specific expression and are activated either by small extracellular ligands, changing CO_2_ concentrations or, in the case of visual guanylyl cyclases, intracellularly interacting Ca^2+^-dependent activating proteins. In this report, we focus on the visual guanylyl cyclase receptors (GCs) GC-E (*gucy2d/e*) and GC-F (*gucy2f*) and their activating proteins (GCAP1/2/3; *guca1a/b/c*). While *gucy2d/e* has been detected in all analyzed vertebrates, GC-F receptors are missing in several clades (reptiles, birds, and marsupials) and/or individual species. Interestingly, the absence of GC-F in highly visual sauropsida species with up to 4 different cone-opsins is compensated by an increased number of guanylyl cyclase activating proteins, whereas in nocturnal or visually impaired species with reduced spectral sensitivity it is consolidated by the parallel inactivation of these activators. In mammals, the presence of GC-E and GC-F is accompanied by the expression of one to three GCAPs, whereas in lizards and birds, up to five different GCAPs are regulating the activity of the single GC-E visual membrane receptor. In several nearly blind species, a single GC-E enzyme is often accompanied by a single variant of GCAP, suggesting that one cyclase and one activating protein are both sufficient and required for conferring the basic detection of light.

## Introduction

**Figure 1:**
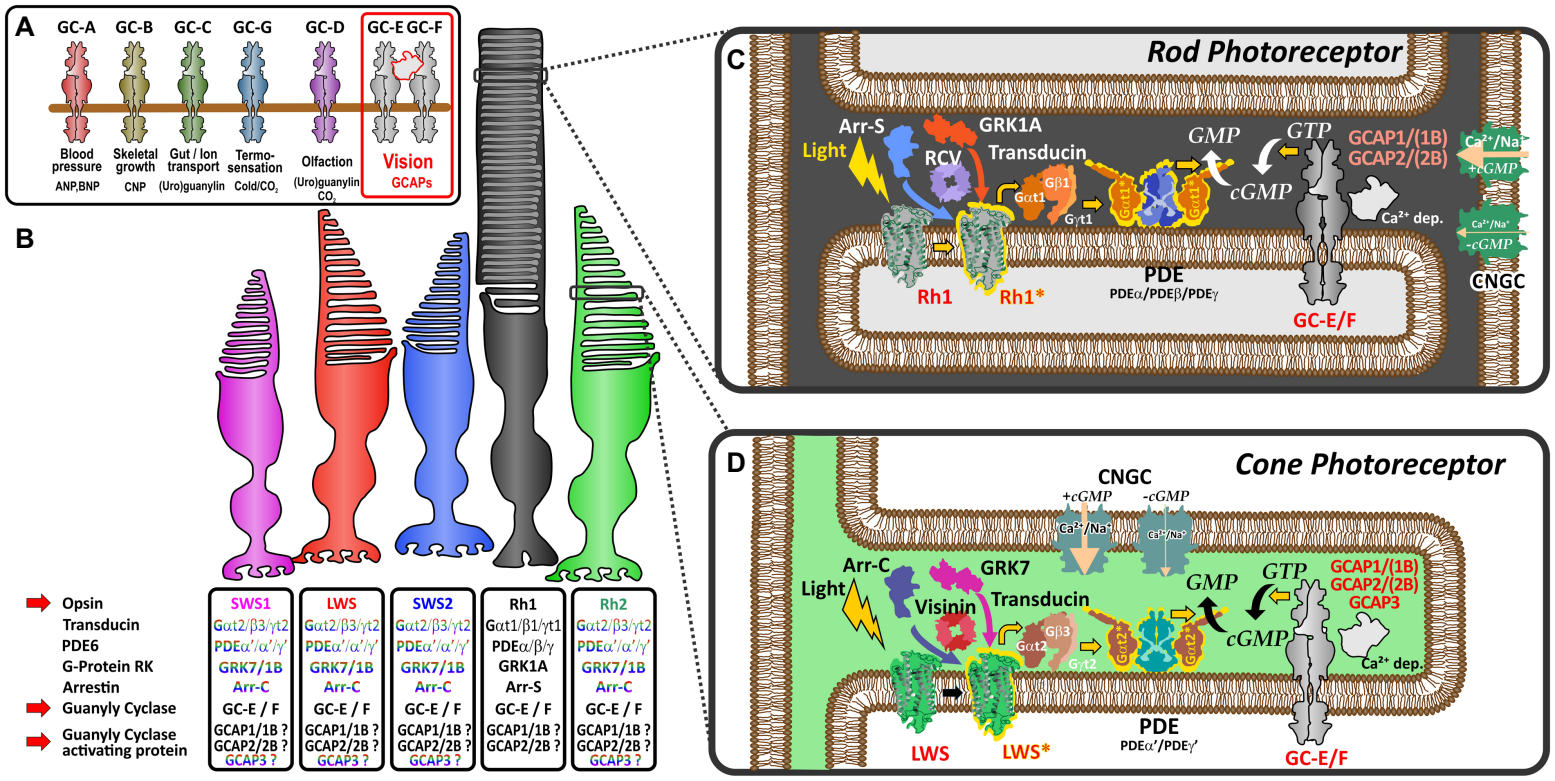
Schematic representation of vertebrate phototransduction and the role of receptor guanylyl cyclases and their activating proteins in vertebrate vision **(A)** Name, activators (ligands) and functions regulated by the different subtypes of membrane guanylyl cyclase receptors. Note that visual guanylyl cyclases have no external activators and are controlled by calcium sensitive intracellular guanylyl cyclase activating proteins (GCAPs). For more details on membrane guanylyl cyclase see Kuhn (2016). **(B)** Photoreceptor subtypes of the vertebrate retina. Four different single cone cell types (represented in color) and one rod cell type (represented in black) have been described in the vertebrate retina. Proteins involved in phototransduction in the different cell types are given in the boxes below the different photoreceptors. The font color indicates that these protein variants are cone specific whereas the black font indicates that cones and rods may use the same proteins to regulate phototransduction. Note that SWS2 and Rh2 expressing photoreceptors are absent in mammals. **(C,D)** Phototransduction cascade in rod **(C)** and cone **(D)** photoreceptors. Note that cones and rods use slightly different proteins in the phototransduction cascade (indicated by the different colors in the picture). Light triggers the activation of visual opsins (Rh1 in rods or SWS1, SWS2, Rh2, and LWS in cones), thereby activating transducin. The activated alpha subunit of transducin stimulates the phosphodiesterase PDE, which after activation increases the hydrolysis of cGMP, lowering the intracellular cGMP concentration. The decreased intracellular cGMP levels result in a closure of cyclic nucleotide gated ion channels (CNGCs). As the activity of the Na^+^/Ca^2+^ K^+^ exchanger NCKX (not depicted in this figure) is not affected by light, intracellular Ca^2+^ levels decrease due to the constant outward flow of Ca^2+^. The decreased Ca^2+^ concentration leads to the activation of calcium dependent guanylyl cyclase activating proteins (GCAPs) which in turn stimulate GCs and the generation of cGMP increasing the intracellular concentration, subsequently reopening CNGCs bringing the Ca^2+^ levels back to the resting stage.

Photoreceptors of the vertebrate retina use distinct sets of proteins to convert light stimulation into neurotransmitter mediated signals. While one set of proteins is involved in the light-induced activation of effector proteins (opsins, transducins, phosphodiesterases and cyclic nucleotide-gated channels), another set of proteins is responsible for shut-off reactions (GRKs, recoverins, arrestins, and RGS proteins) following light stimulation. Subsequently, visual guanylyl cyclase and their activating proteins play an essential role in the calcium feedback system of vertebrate phototransduction (Lamb and Hunt, 2018; Gesemann and Neuhauss, 2020; Lamb, 2022) for resetting the detection cascade (Figures 1C,D).

Dim-light specialized rod and bright-light adapted cone photoreceptors often use a related, but slightly different set of phototransduction cascade proteins (Figure 1B; Lamb, 2013). The basis for this cell type specific specialization originates in two rounds of whole genome duplications (WGD) that shaped the vertebrate genome some 500 million years ago. In several reports Lamb and colleagues have demonstrated that most phototransduction genes originate from one ancestral region that is now represented in no more than five paralogous regions in extant vertebrates (Lamb, 2013, 2022). However, several genes have already experienced local duplications prior to WGD, suggesting that the origin of specialization of phototransduction predates the genome duplication events. As a result of these local and WGD duplications, a variable number of copies of ancestral phototransduction genes have adopted specialized or new functions and are expressed in a cell-type specific manner. This is particularly apparent for the different visual pigments, such as rhodopsin and the color sensitive red-, green-, blue- and UV-opsin based pigments that are found in the different types of photoreceptors. Furthermore, such a specialization can also be seen for transducin, PDE6 subunits, CNG channel subunits, arrestins, GRKs, recoverins and NCKX ion exchangers (Lamb, 2022). However, for some proteins of the phototransduction cascade, like guanylyl cyclases (GCs) and their activating proteins (GCAPs), an exclusive cell type specific expression has not been demonstrated and they are present in different types of photoreceptors (Figure 1).

In the tetrapod lineage two visual (GC-E and GC-F) and one phylogenetically closely related olfactory guanylyl cyclase receptor (GC-D) have been identified (Lamb and Hunt, 2018; Gesemann and Neuhauss, 2020). While GC-E is expressed in rods and cones (Dizhoor et al., 1994), GC-F is predominantly expressed in rods (Yang et al., 1995; Yang and Garbers, 1997). Moreover, GC-F expression is generally more restricted and severalfold lower than that observed for GC-E (Helten et al., 2007). These findings go hand in hand with the notion that disruption of *gucy2e* in mice causes severe cone dystrophy, while having little or no effect on rod morphology (Yang et al., 1999). In contrast, mice lacking both cyclases not only completely lack a light induced electrical response but also show a progressive degeneration of rod and cone photoreceptors (Baehr et al., 2007). Similar phenotypes are seen in human patients having mutations in *GUCY2E* (originally called *GUCY2D*), whereas no mutations in *GUCY2F* affecting phototransduction have been described so far (Perrault et al., 1996, 2000).

Neuronal calcium sensor proteins (NCS), which are highly conserved EF-hand superfamily proteins, tightly regulate the activity of GCs. More than 20 different members of the NCS family have been described, with some of them being species specific (Lim et al., 2014; Ames, 2018). Among these, six subfamilies fall into the category of guanylyl cyclase activating proteins (GCAPs), with teleost fish possessing additional 3R and 4R duplicates (Imanishi et al., 2004; Lamb, 2022). The most intensely studied members of this subfamily are *guca1a* (GCAP1) and *guca1b* (GCAP2), which have been shown to be expressed at different levels in rod and cone photoreceptors (Cuenca et al., 1998). While GCAP1 seems to be the predominant variant in cones, GCAP2 expression levels are higher in rods. Mutations in both proteins have been shown to cause retinal dystrophies, albeit with more severe phenotypes seen for *guca1a* (GCAP1) mutations (Sato et al., 2005; Behnen et al., 2010; Jiang and Baehr, 2010). Due to the often species-specific presence, far less is known about function and abundance of the other four members of the GCAP subfamily.

In a previous study we have analyzed the evolution of guanylyl cyclase following the two rounds of WGD (Gesemann and Neuhauss, 2020). Some clades and isolated species lack either GC-F, GC-D or both receptors relying on a single membrane linked cyclase to control the calcium feedback system of phototransduction. Interestingly, not only have species with reduced visual capabilities lost the second visual guanyly cyclase, but the same is true for some entire clades comprised of species with excellent vision, such as birds and lizards. We have therefore hypothesized that g*ucy-2f* gene loss occurring in ancestral or recent “bottleneck” species, which affects all extant downstream species, might be compensated by functional adaptation and/or duplication of other phototransduction cascade proteins. In contrast, gene loss in isolated lineages might be the result of adaption to specific habitats. In this report, we focus on the evolution of GCAPs in all major tetrapod clades following 1R and 2R and concentrate on a variety of highly adapted underground living species that have only limited visual capabilities. Our results show that while birds and lizards react to the sole presence of one GC with an increase in number of GCAPs, in visually impaired species the observed GC gene reduction is often mirrored by a similar reduction in GCAP gene content.

## Materials and methods

### Annotation of guanylyl cyclase activating sequences

As gene and cDNA sequence annotations created by computerized methods have been shown to contain frequent errors, cDNA sequences of guanylyl cyclase activating proteins (*guca’s*), additional *gucy* genes and selected opsin genes used in this study were manually annotated. Sequences were identified and annotated using information from genome databases, nr/nt database, wgs contigs and expressed sequence tags (GeneBank,^1^ last accessed February 8th, 2023; Ensembl,^2^ last accessed December 8th, 2022). Mouse and human sequences were used as initial query [for more details on sequence annotation, see Gesemann et al. (2010)]. Nonsense as well as putative frame shift mutations within the manually annotated sequences were confirmed using whole genome shotgun (WGS) contigs and/or additional sequence information from expressed sequence tags. Only sequences with more than one inactivating or splice site mutation or with corresponding mutations in closely related species were considered relevant. Genome sequences with poor quality (multiple Ns in the extracted sequence or large sequence parts missing) were excluded from the analysis. Exon sizes as well as putative cDNA sequences from related species were used as additional references. Intron/Exon boundaries were identified using a combination of the genescan webpage^3^ and manual inspection analyzing major as well as minor splice site consensus sequences (Patel and Steitz, 2003). A complete list of *guca* intron and exons sizes and putative inactivating mutations as well as the genomic region used is given in the Supplementary Table S1. Regions of annotated mammalian opsin sequences are given in Supplementary Table S2 and corresponding regions of sauropsida opsins are given in Supplementary Table S3.

### Generation of phylogenetic trees

Phylogenetic trees were generated using the NGPhylogeny.fr platform^4^ (last accessed February 17th, 2023) (Dereeper et al., 2008; Lemoine et al., 2019). 117 cDNA sequences of *guca1a* were aligned using MAFFT version 7 with default parameters (Katoh and Standley, 2013). The length of input sequences varied between 588 and 636 nucleotides. After alignment, ambiguous regions (i.e., containing gaps and/or being poorly aligned) were removed with BMGE (Block Mapping and Gathering with Entropy) (Criscuolo and Gribaldo, 2010) using default parameters: Sliding window size: 3; maximum entropy threshold: 0.5; gap rate cuttoff: 0.5; minimus block size: 5; matix: PAM250. Following curation 602 nucleotides (some sequences with gaps) were used for further analysis. Phylogenetic tree reconstructions were done using the FastTree method (Price et al., 2009). Graphical representations of the phylogenetic trees were obtained using iTol utility^5^ and edited in Coral draw (CoralCorporation).

### Synteny analysis

An initial rough synteny analysis was done using the synteny database^6^ (last accessed November 14th, 2022) (Catchen et al., 2009). Synteny hits in the output files were further subjected to microsynteny analyses, where paralogous/orthologous genes of all the species of interest were analyzed in Ensembl (see Footnote 2) (last accessed December 20th, 2022) and confirmed by reciprocal tBLASTx searches. Final figures were edited using Coral draw (CoralCorporation).

### Animal pictures

Animals pictures used in our figures were downloaded from the shutterstock photo repository^7^ using a purchased license.

## Results and discussion

### Phylogeny and synteny analysis confirm absence of GC-F In sauropsids

**Figure 2:**
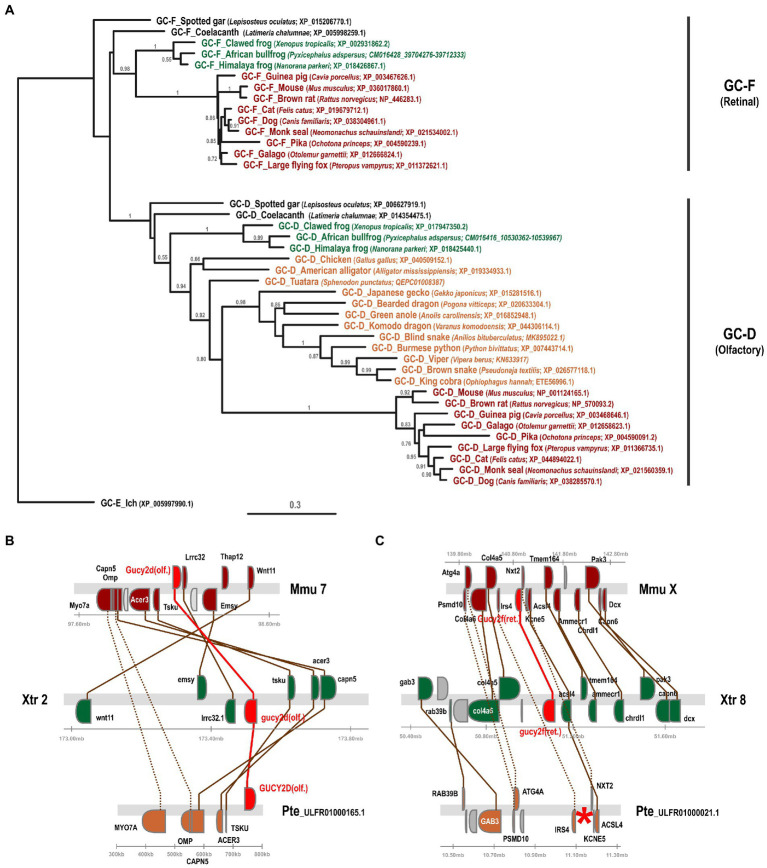
Phylogeny and synteny of *gucy2f*/GC-F (retinal) and *gucy2d*/GC-D (olfactory) confirm the absence of *gucy2f*/GC-F in sauropsids. **(A)** Phylogenetic tree of *gucy2f* and *gucy2d*. *gucy2f* as well as *gucy2d* (olf.) genes of selected mammalian (displayed in red) and amphibian (shown in green) species that have retained both genes were compared to the *gucy2f/d* genes described in sauropsids (depicted in orange). Common and scientific names for the species are given. The scale bar shows the percentage (0.3 equals 30%) of nucleotide substitutions required to generate the corresponding tree. Note that no *gucy2f* sequences are present in sauropsids. (B and C) Synteny of mouse (mmu), xenopus (xtr) and eastern brown snake (pte) *gucy2d* (olf.) **(B)** and *gucy2f* **(C)**. Genomic regions around the mouse, xenopus and eastern brown snake *gucy2d* **(B)** and *gucy2f* **(C)** are shown and the chromosomal location is indicated. The location of the *gucy* genes is highlighted in red and syntenic genes are given in colors. Orthologous genes are connected by colored lines, indicating that the vicinity of the *gucy2d* gene is conserved in all three species **(B)**, whereas, even if the vicinity of *gucy2f* is conserved between the species, the gene is absent in the eastern brown snake **(C)**. A red asterisk indicates the putative location of the missing gene.

In a previous study we have investigated the preservation, inactivation and possible functional adaptation of guanylyl cyclase genes across more than 300 different vertebrate species (Gesemann and Neuhauss, 2020). We have shown that eutherians commonly use two visual GCs, namely GC-E and GC-F. However, independent pseudogenization of the GC-F receptor occurred in some lineages, such as sauropsids and mustelidae (weasel-like) and some isolated, often non visual, species. A more recent report, however, suggested that sauropsids might still have two visual guanylyl cyclases (Gower et al., 2021), which is in contradiction to what we and Lamb and colleagues have reported. Therefore, we first reexamined our previously annotated sequences and compared them to the available sequences in the different databases and available information in the latest reports. In order to get a more conclusive overview, we used species that have both GC-F and GC-D variants and compared them to the putative *gucy2d/f* sequence of species of the sauropsida clade using phylogeny and synteny. Phylogeny as well as synteny analysis clearly indicated that the designated sauropsida GC-F variants group into the olfactory GC-D branch (Figure 2A). Remarkably, the relation between mammalian GC-D variants and the corresponding sauropsida ones is quite distant, as sequence conservation between these two clades is only in the range of 50%. Nevertheless, the sauropsida GC-D/F protein sequences are clearly more closely related to the GC-D sequences of anura (frogs) or coelacanth and holostei fish than that they are to their corresponding GC-F sequences. This view is further supported by the synteny of *gucy2f* (GC-F) and *gucy2d* (olf.) (GC-D) (Figures 2B,C). The gene alignment in the vicinity of mouse and Xenopus *gucy2d* (olf.) is evidently preserved in the eastern brown snake *Pseudonaja textilis* (Figure 2B). However, gene synteny in the region of mouse and Xenopus *gucy2f* clearly indicates that *gucy2f* is missing in the eastern brown snake. Taken together, we conclude that our previous analysis that sauropsids lack the second visual guanylyl cyclase GC-F holds true. This is further supported by the finding that none of the 13 lepidosauria eye transcriptomes detected a GC-F variant (Simões et al., 2018).

### Loss of *gucy2f*/GC-F in sauropsids can be compensated by an increasing number of GCAPs

Having confirmed our previous findings on *gucy* gene evolution across species, we were then interested in the influence of *gucy* inactivation on the abundance of GCAPs, in particular in the context of the visual requirements of the analyzed species.

We initially assembled *guca* coding sequences of same species that we have previously used for our g*ucy* analysis (Gesemann and Neuhauss, 2020). However, in the current study we omitted species, such as teleosts, whose genomes underwent an additional round of whole genome duplication, but included some additional species that have been shown to have either relatively poor eyesight or excellent visual capabilities. The coding sequences of all assembled *guca’s* are spread over four exons which between paralogs are very similar in size but display slight, consistent variations that are typical for the different *guca* variants (Supplementary Table S1). Introns of *guca1c* are constantly larger (3 to 15 times) than the corresponding introns of *guca1a* and *guca1b* derived paralogs (Supplementary Table S1), suggesting that *guca1c* might be differently regulated than the other activating proteins.

**Figure 3:**
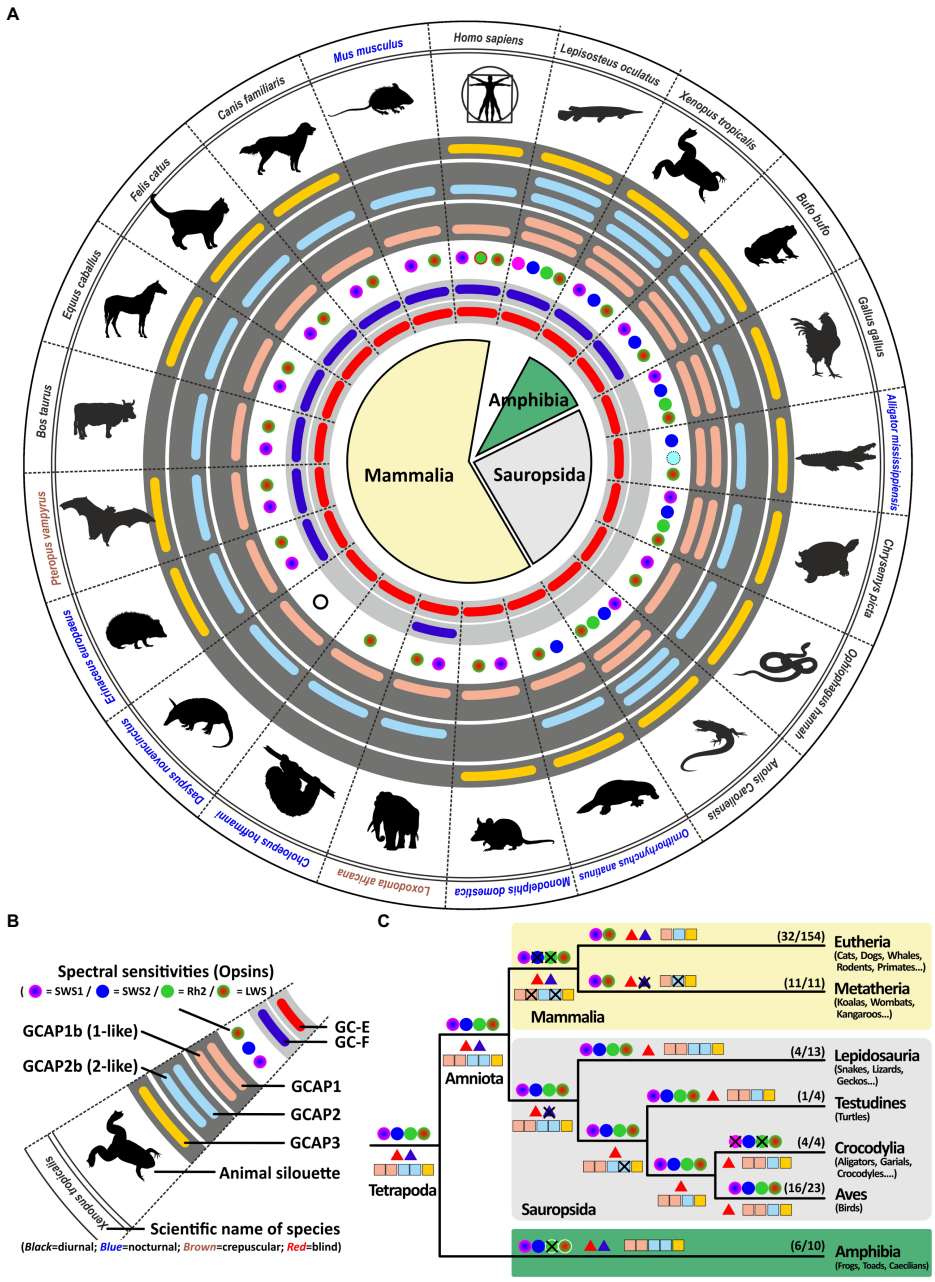
Reduction of visual GCs can be compensated by an increased number of GCAPs. **(A)** Expression of opsins, GCs and GCAPs in major vertebrate species. In the center, major vertebrate classes and clades are given. Mammals are depicted in light yellow, sauropsids in light gray and amphibians in green. **(B)** Explanation of symbols used in **(A)**. Pictograms for each chosen representative are shown and scientific names are given. Names of diurnal species are written in black, crepuscular species in brown and nocturnal species in blue. The presence of an open reading frame coding for GC-E is shown as a red bar in the innermost light gray circle and *gucy2f* genes without inactivating mutation are illustrated by a dark blue bar adjacent to it. The different types and color sensitivities of photoreceptor single cones are indicated by the colored dots and eventual rod monochromacy is shown by a white circle. Spectral sensitivities under debate are indicated by dotted lines. The presence or absence of intact ORFs for GCAPs are given by the colored bars in the dark gray circles. *guca1aa* and *guca1ab* (from inside to outside) are given in light red, *guca1ba* and *guca1bb* are given in light blue and *guca1c* is shown in yellow. **(C)** Evolution of opsins, GCs and GCAPs in major vertebrate clades. Major vertebrate linages are shown. Opsins are given by circles, GCs by triangles and GCAPs by squares. The most likely ancestral situation at every major branch point is given. Numbers in parenthesis represent the species still displaying this gene content (first number) and the total number of species analyzed (second number). Note that sauropsids lack the second visual GC, but have often a higher number of intact GCAPs when compared to eutherians.

Previous *in vitro* experiments have suggested that GCAP1 (encoded by *guca1a*) and GCAP2 (encoded by *guca1b*) are similarly active in stimulating GC-E and GC-F receptors (Koch and Stryer, 1988). However, more recent *in vivo* data from rod photoreceptors indicate that GC-E is predominantly regulated by GCAP1 (Olshevskaya et al., 2012). Therefore, we expected GCAP1 to be the dominant GCAP protein with GCAP2 and GCAP3 being under lower evolutionary pressure. In a first survey analysis, we selected one to two species per clade that are frequently used in scientific studies or represent key species for phylogenetic analysis. Among these analyzed species are 12 mammals; covering primates, rodents, carnivores, herbivores, bats, afrotherians and marsupials; 1 egg laying *prototheria*; five *sauropsida* species, including lizards, birds, turtles, crocodiles; two amphibians as well as 1 holostei fish species. As previously reported, all sauropsids and marsupials lack the second visual guanylyl cyclase (Lamb and Hunt, 2018; Gesemann and Neuhauss, 2020). This is also true for the mostly nocturnal monochromatic sloth *Choloepus hoffmanni*. As suggested in our previous report, sauropsids indeed compensate the loss of *gucy2f* with an increased number of *guca* genes (Figure 3). This is particularly striking for the lizard *Anolis caroliensis* in which we found five GCAPs, but also in chicken and turtles we detected four GCAP variants. Snakes only require one guanylyl cyclase and three guanylyl cyclase activating proteins. In general, visual requirements are reflected by the combinatorial possibilities of guanylyl cyclases and their activating proteins. Highly visual trichromats, such as humans, have six combinatorial possibilities (2 × 3), while dichromatic mammals such as elephants and cattle get by with four possibilities (2 × 2). Lizards on the other hand have five combinatorial possibilities, whereas birds and turtles have four and snakes still have three different possibilities. Interestingly, frogs and holeostei fish have the highest number of possibilities, as they possess both visual GCs in combination with five activating proteins.

### Visual system adaptation in sauropsids is reflected in the number of active GCAPs

**Figure 4:**
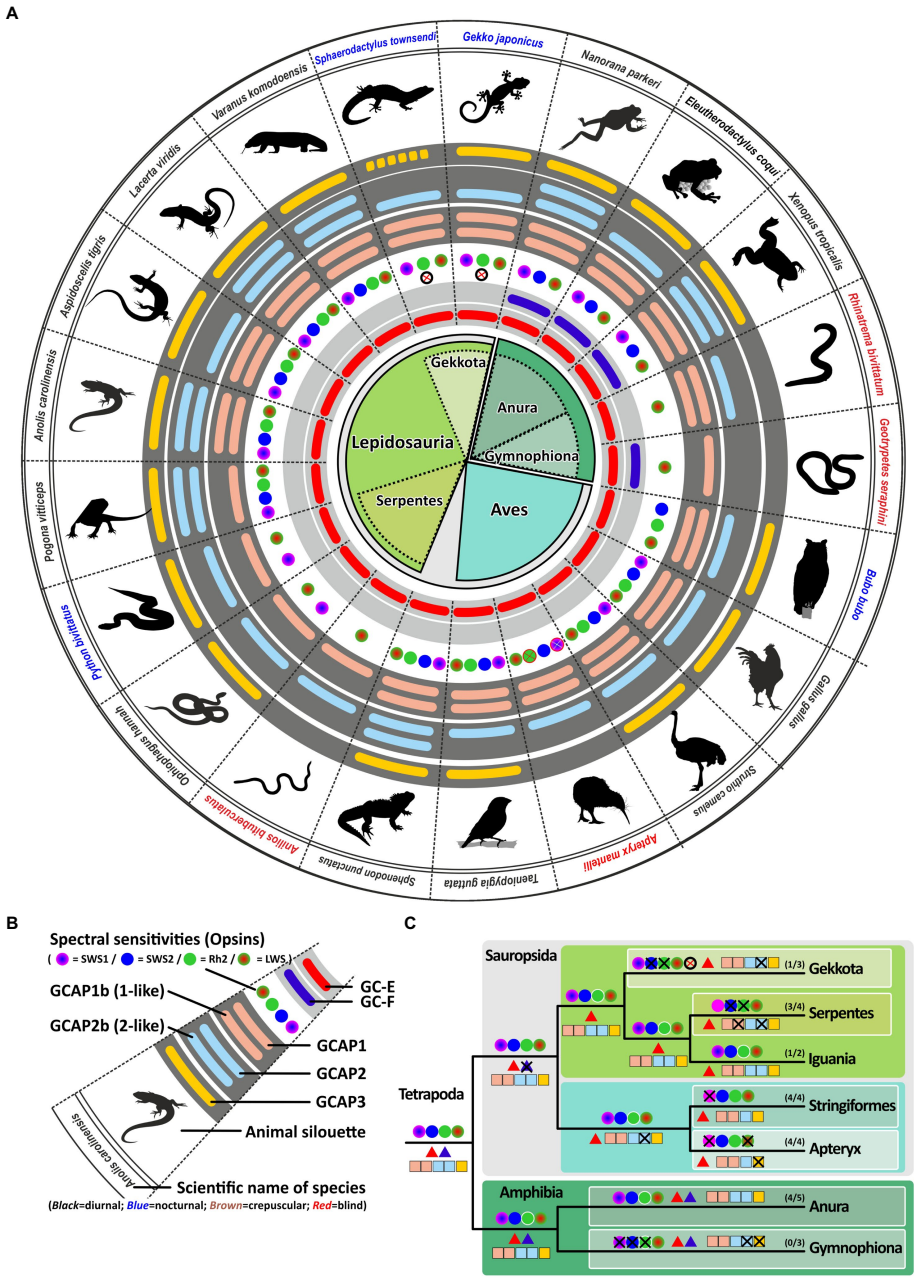
Reduction to one visual GC in sauropsids represents a bottleneck in the phototransduction calcium feedback system. **(A)** Expression of opsins, GCs and GCAPs in sauropsids and amphibians. In the center, major sauropside and amphibian classes, orders and suborders are given. Scaled lizard (*Lepidosauria*) are depicted in bright green, birds (*Aves*) in petrol, and amphibians in dark green. Suborders are indicated by a lighter shade. **(B)** Explanation of symbols used in **(A)**. Pictograms for each chosen representative are shown and scientific names are given. Names of diurnal species are written in black, nocturnal species in blue and blind or visually impaired species in red. The presence of an open reading frame encoding GC-E is shown as a red bar in innermost light gray circle and *gucy2f* genes without inactivating mutation are illustrated by a dark blue bar adjacent to it. The different types and color sensitivities of photoreceptor single cones are indicated by the colored dots. In the case of geckos the crossed out white circle indicates the inactivation of the Rh1 gene. Crossed out circles in the kiwi case represent unverified losses. The presence or absence of intact ORFs for *guca* transcripts are given by the colored bars in the dark gray circles. *guca1aa* and *guca1ab* (from inside to outside) are given in light red, *guca1ba* and *guca1bb* are given in light blue and *guca1c* is shown in yellow. **(C)** Evolution of opsins, GCs and GCAPs in major sauropsid and amphibian clades. Major lineages are shown. Opsins are given by circles, GCs by triangles and GCAPs by squares. The most likely ancestral situation at every major branch point is given. Numbers in parenthesis represent the species still displaying this gene content (first number) and the total number of species analyzed (second number). Note that blind species have to the lowest number of combinatorial possibilities for *guca* and *gucy* genes, and that highly visual animals keep a higher number of combinations.

As visual performances among the different sauropsida and amphibian species can vary dependent on their ecological niches and lifestyle adaptation, we compared the integrity of *guca* genes in habitat extremists with more generalist species. In the snake (*serpents*) clade which includes the mainly nocturnal constricting snakes (pythons, boas and anacondas), crepuscular and diurnal vipers and the ground dwelling fossorial blind snakes, lifestyle has some effects on opsin (Gower et al., 2021) gene content. Compared to lizards which are generally tetrachromats, nocturnal and diurnal snakes are usually dichromatic and burrowing blind snakes are either rod or cone monochromats (Gower et al., 2021; Figure 4). The reduced number of cone subtypes is reflected in the amount of functional GCAPs. Highly visual lizards combine their single visual guanylyl cyclase with up to five GCAPs, while neither analyzed serpent has more than three GCAPs and in the case of the blind snake *Anilios bituberculatus* this number is further reduced to only GCAP1 or GCAP2, respectively. However, some lizards and especially the more distantly related *gekkota* clade show with three or four GCAPs, also a reduced gene number. In the case of geckos this might be explained by their nocturnal lifestyle and the absence of rhodopsin and a fourth visual cone pigment (Pinto et al., 2019). However, not all diurnal lizards follow this pattern.

Tetrachromatic birds which represent a sister clade of the previously discussed Lepidosauria (scaled lizards), also have a maximum of 4 GCAPs. This suggests that all visual demands, even for highly visual tetrachromatic animals, can be covered by four GCAPs. Establishing a link between chromaticity and GCAPs in birds is however difficult as visual pigment genes in birds, with the exception of rh1 and rh2, are likely encoded on microchromosomes that often cause genomic sequencing problems (Waters et al., 2021). This might be the reason why tetrachromacy on the genomic level could not be confirmed in a variety of species (Borges et al., 2015). In the chicken case tetrachromacy is anatomically established (Kram et al., 2010) and could be confirmed by the identification of four visual pigment cDNA sequences (Enright et al., 2015; Yamagata et al., 2021), however, on a genomic level the *opsin1sw1* sequence is missing. This suggests that microchromosome sequence evasion is indeed a problem, even in species with high sequence coverage and broad scientific interest. Nevertheless, recent studies imply that owls lack the *opsin1sw1* gene (Hanna et al., 2017; Höglund et al., 2019), suggesting that a nocturnal or crepuscular lifestyle indeed influences vison in birds. Interestingly, we did not see a reduction of GCAPs, as all analyzed owl species still show four GCAPs (Supplementary Table S1).

While owls as nocturnal raptors have large eyes and excellent vison, another family of nocturnal birds, namely New Zealand kiwis, have greatly reduced eyes and in general poor vision (Corfield et al., 2015). Genome sequencing in kiwis indeed suggested that several opsins are either absent or have accumulated deleterious mutations (Le Duc et al., 2015). While the problem with sequencing GC-rich microchromosomes is acknowledged in this report, all expected opsin sequences could be identified. Several mutations in *opsin1sw1* and *rh2* (*opsin1mw*) seem to be present most likely rendering these chromophores non-functional (Le Duc et al., 2015). When we looked at kiwi opsin genes we could confirm alterations in the *rh2* sequences causing a frame shift in the 4^th^ exon (Supplementary Table S1). However, we found no mutations in the partial *opsin1sw1* sequences of *Apteryx mantelli* and *Apteryx haastii*. Due to incompleteness of the *opsin1sw1* sequence in these species, the integrity of this gene remains unclear. In contrast to owls, the inactivation of opsins in kiwis is also reflected in accumulating mutations in the kiwi *guca1c* gene. As these mutations are present throughout the four analyzed kiwi genomes we are confident that this is indeed an occurring non-functionalization. We have also looked at a number of other nocturnal birds such as the kakapo, night heron, nightjar and others, but did not find obvious *guca* gene variation (Supplementary Table S1), suggesting that as for owls preserving four intact GCAPs might be beneficial for nocturnal as well as cathemeral species.

Two other sister clades of birds and reptiles are turtles and crocodiles. As land turtles are slow moving herbivores protected from predators by their stable shells, a good sense of vision seems not mandatory. Nevertheless, tetrachromatic vision still occurs in turtles (Emerling, 2017; Corredor et al., 2022) and we have confirmed the tetrachromacy of *Chrysemys picta* (Supplementary Table S3). A possible explanation may lie in the better ability of tetrachromats to distinguish color hues in fruits, part of the diets of turtles. In contrast to a previous report by Emerling and colleagues, we could only find open reading frames for three opsins in the aquatic turtle *Chelonia mydas* (*rh2*, *opsin1sw1* and *opsin1lw*) and the Pinta giant tortoise *Chelonoidis abingdonii (opsin1sw2, rh2 and opsin1lw)*. Interestingly, some of the previously published sequences have been removed, even if one of the removed sequences is clearly the *opsin1lw* sequence (XM_007052772.1). The other removed sequence (XM_007067421.1) is identical to a newer sequence (XM_037889438.1) both representing the *opsin1sw1* transcript. The transcript XM_007063507.2, which was and still is considered to be *opsin1sw2* is likely a *rh2* paralog (Supplementary Table S3). No *opsin1sw2* transcript or genomic sequence could be identified suggesting that *Chelonia mydas* is trichromatic rather than tetrachromatic. As suggested by Emerling and colleagues we also found the Chinese softshell turtle *Pelodiscus sinensis* to be most likely dichromatic, as *opsin1sw1* and *opsin1sw2* have accumulated various mutations, rendering the genes potentially non-functional (Supplementary Table S3). Interestingly, the amount of functional cone opsins has no immediate effect on the abundance of GCAP genes, as four of these proteins acting downstream in the rod and cone phototransduction pathway are present and most likely functional in all analyzed turtles (Supplementary Table S1).

The situation in the crocodile clade is somewhat different. The mainly nocturnal crocodiles have reduced their opsin gene content to two cone opsins (*opsin1sw2* and *opsin1lw*) and one regular rhodopsin (*rh1*) (Emerling, 2017). This is reflected by two morphological single cone types in caimans and alligators. However, in the case of the salt water crocodile *Crocodylus porosus*, the existence of a third single cone has been proposed (Emerling, 2017), in which the rod rh1 pigment is also used in cone photoreceptors, making these animals functional trichromats. Interestingly, dichromacy seems not to go hand in hand with a reduction of GCAPs as all crocodiles still have four GCAPs (Supplementary Table S1).

### Amphibian visual spectra and number of GCs and GCAPs are highly variable

A previous study by Schott and colleges (Schott et al., 2022b) has shown that *Anura* (frogs and toads) are in general trichromats, expressing *opsin1sw1*, *opsin1sw2* and *opsin1lw*. In contrast to sauropsida which have downstream of phototransduction only one guanylyl cyclase to regulated calcium feedback, amphibians have, similar to eutherians, two cyclases at their disposal (Gesemann and Neuhauss, 2020). In the case of eutherians (placental mammals), neither species expressing both visual cyclase genes has more than three guanylyl cyclase activating proteins, suggesting that all aspects of trichromatic vision, as observed in humans and old world monkeys, can be regulated by a 2/3 (2 guanylyl cyclases and 3 GCAPs) combination (Gesemann and Neuhauss, 2020). Surprisingly, frogs and toads seem to have preserved a more ancestral gene state as they have, similar to lizards and holostei (e.g., gars and bowfins), still five functional GCAPs (Figure 4) enabling 10 different combinatorial possibilities. While frog vision is well developed, there is at first glimpse no reason why frogs and toads should combine two visual guanylyl cyclases with five activating proteins. Frogs and toads in general go through two different developmental stages, hence the combination of two guanylyl cyclases with five activating proteins may very well be lifestyle related. They not only switch from an aquatic habitat to a more terrestrial lifestyle following metamorphosis from tadpoles to frogs, but they also show changes in the expression of opsins as well as other genes of the visual system during this transition (Schott et al., 2022a). This adaptive decoupling in the visual system might go hand in hand with anatomical changes observed in the tadpole versus frog retina. In the case of guanylyl cyclases and their activating proteins, adaptive decoupling might lead to differential expression of these genes during tadpole and adult stages. While some might be expressed in larval stages, other could be active in adults only. To test this hypothesis we analyzed the GCAP situation in the common coquí *Eleutherodactylus* coqui, a frog species that has no free swimming tadpoles (Townsend and Stewart, 1985; Westrick et al., 2022). Coquis lay eggs in palm trees and the larvae remain in the eggs until mature frogs emerge, thereby bypassing the tadpole stage. Indeed, *Eleutherodactylus coqui* was the only anuran species in which we identified only four CGAPs (Figure 4; Supplementary Table S1), suggesting that tadpoles and adult frogs may indeed rely on different GCAPs explaining their increased number in amphibians.

A sister clade to frogs, toads and salamanders are caecilians, blind, limbless worm-like amphibians. Caecilians mostly live underground and their vision is mainly limited to dark–light perception. Previous studies have shown that caecilians are rod monochmomats, lacking cone photoreceptors (Mohun et al., 2010; Mohun and Wilkinson, 2015). This adaptation to underground life is also reflected in the reduction of GCs and GCAPs. While in *Geotrypetes seraphini* the reduction is seen at the level of GCAPs, in *Rhinatrema bivittatum* the reduction occurs at the level of the cyclases (Figure 4) leaving them with either two or three combinatorial possibilities, respectively.

### Lifestyle adaptations in rodents, rabbits and insectivores cause alteration in the calcium feedback system of phototransduction

**Figure 5:**
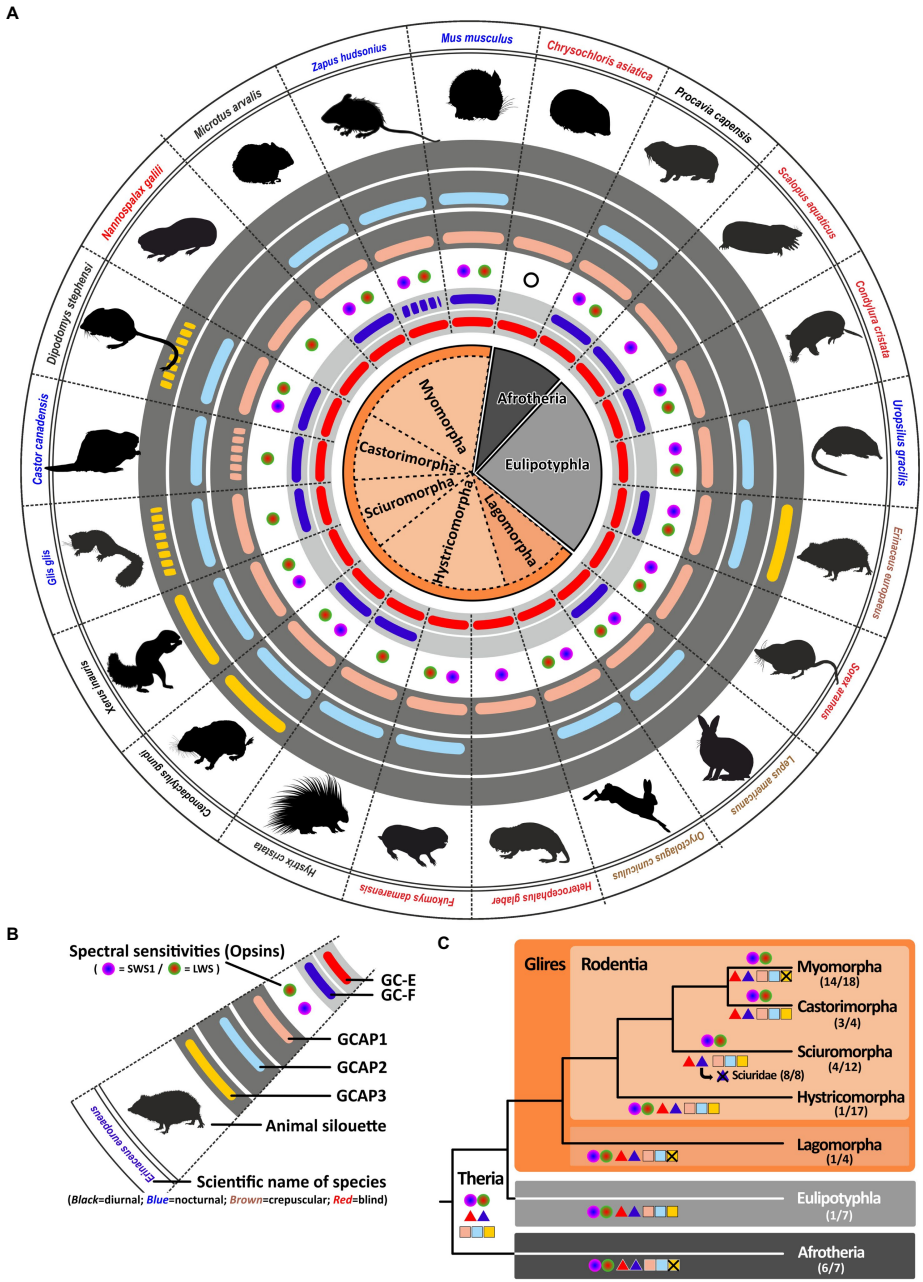
Regressive eye evolution in rodents and insectivores is reflected in the pseudogenization of *gucy* and *guca* genes. **(A)** Expression of opsins, GCs and GCAPs in glires, insectivores and afrotherians. In the center, major rodent and rabbit as well as insectivore and afrotherian orders and suborders are given. Glires are highlighted in brown, insectivores in gray and afrotherians in dark gray. Suborders (rodents and rabbits) are indicated by a lighter shade. **(B)** Explanation of symbols used in **(A)**. Pictograms for each chosen representative are shown and scientific names are given. Names of diurnal species are written in black, crepuscular species in brown, nocturnal in blue and blind or visually impaired species in red. The presence of an open reading frame encoding the GC-E protein is shown as a red bar in innermost light grey circle and *gucy2f* genes without inactivating mutation are illustrated by a dark blue bar adjacent to it. The different types and color sensitivities of photoreceptor single cones are indicated by the colored dots and rod monochromacy is shown by a white circle. The presence or absence of intact ORFs for *guca* transcripts are given by the colored bars in the dark gray circles. *guca1aa* and *guca1ab* (from inside to outside) are given in light red, *guca1ba* and *guca1bb* are given in light blue and *guca1c* is shown in yellow. Sequences that have only a single mutation, that could be based on a sequencing error, are given by broken lines. **(C)** Evolution of opsins, GCs and GCAPs in major glires, insectivores and afrotherian orders. Major linages are shown. Opsins are given by circles, GCs by triangles and GCAPs by squares. The most likely ancestral situation at every major branch point is given. Numbers in parenthesis represent the species still displaying this gene content (first number) and the total number of species analyzed (second number). Note that for some nearly blind species the *gucy* and *guca* gene number has been reduced to one single copy each.

Similar to caecilians, many animals of the rodent, rabbit and insectivore clade are underground living or at least nocturnal animals, with often reduced vision. In general, animals of the *glires* and *insectivore* clade are dichromatic. However, there are reports having shown that some underground specialists have reduced their active opsin complement (Peichl et al., 2000; Emerling and Springer, 2014). The blind mole rat *Nannospalax galili*, the naked mole rat *Heterocephalus glaber*, the cape golden mole *Chrysochloris asiatica* and the Eastern mole *Scalopus aquaticus* are either cone or rod monochromats (Emerling and Springer, 2014; Emerling, 2018); (Figure 5). We have previously shown that reduced visual capabilities are frequently reflected in a reduction of proteins involved in the calcium feedback system of phototransduction, namely guanylyl cyclases (Gesemann and Neuhauss, 2020). However, not all of these animals have completed the inactivation of the second visual GC, as the Eastern mole has still two intact visual GC sequences (Figure 5). There are nocturnal animals such as the porcupine *Hystrix cristata* and the European dormouse *Glis glis* that have both visual GCs but are cone monochomats. Interestingly, all the above mentioned visually impaired mole species rely also on only one GCAP, independent whether they poses one or two visual GCs. In contrast to this, dormice and porcupines have two GCAPs leaving them with four combinatorial possibilities. Remarkably, some visually impaired species such as the star nosed mole *Condylura cristata* and the common shrew *Sorex araneus* remain functional dichromats (Emerling, 2018). Nevertheless, they both only possess the *guca1a* gene and in the case of the shrew also only one *gucy* gene. While vision has in general little importance for rodents, rabbits and insectivores, there are some notable exceptions. Squirrels for instance have excellent vision (van Hooser and Nelson, 2006). Even if they have only one visual GC they possess as almost the only clade of this order three GCAPs. This is hardly surprising, as squirrels are diurnal animals that highly depend on visual input especially for tree-dwelling species.

### Spectral sensitivity as well as nocturnality influences the calcium feedback system of phototransduction in carnivores and *Xenarthra*

**Figure 6:**
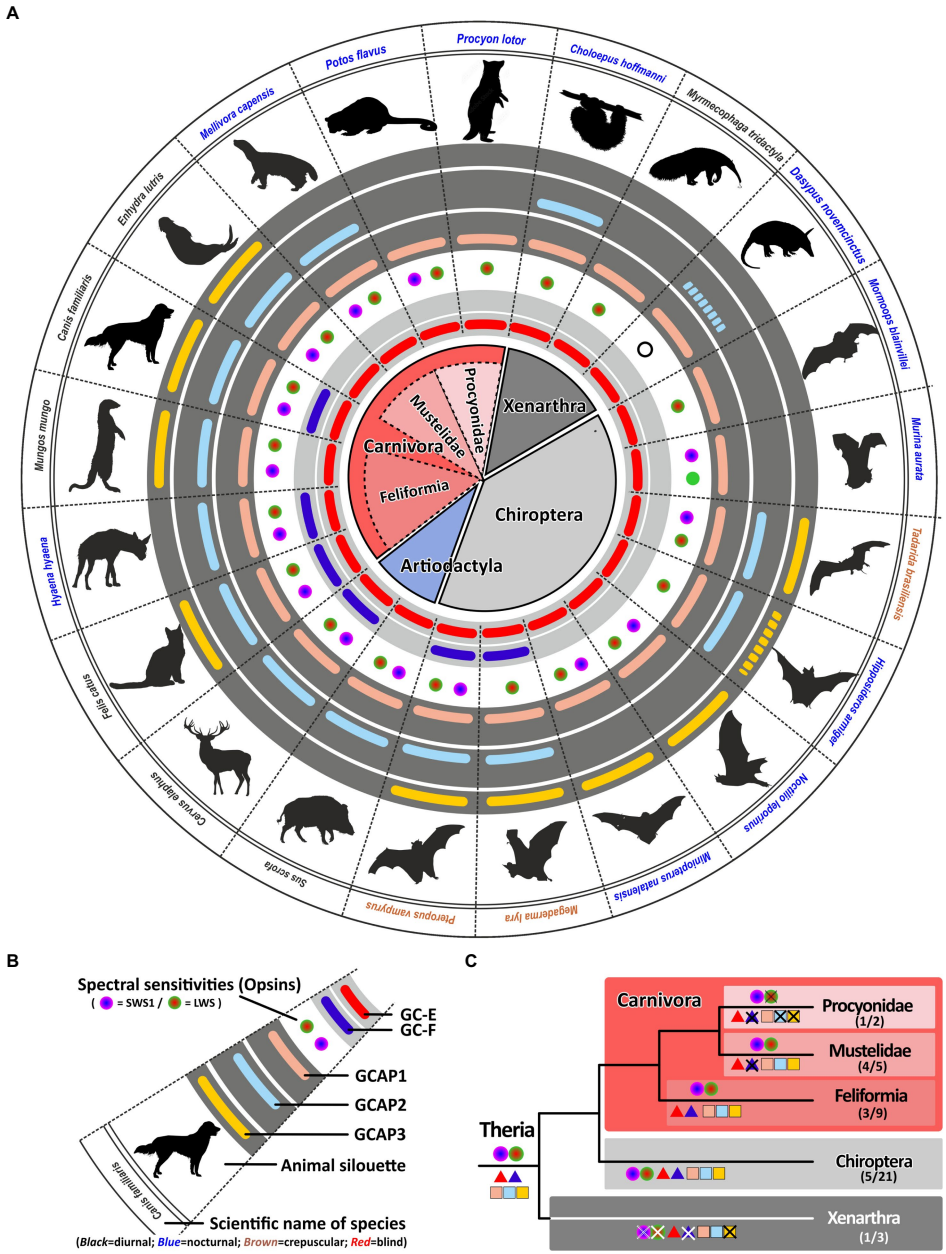
Monochromacy in carnivores, xenarthra and bats reduces the number of *gucy* and *guca* genes with intact ORFs. **(A)** Expression of opsins, GCs and GCAPs in carnivores, even toed ungulates, bats and xenarthras. In the center, major orders and suborders are given. Carnivores are highlighted in red, bats in gray and xenarthras in dark gray. Carnivore suborders are indicated by a lighter shade. **(B)** Explanation of symbols used in **(A)**. Pictograms for each chosen representative are shown and scientific names are given. Names of diurnal species are written in black, crepuscular species in brown and nocturnal species in blue. The presence of an open reading frame for *gucy2e* (*gucy2d* retinal) is shown as a red bar in the innermost light gray circle and *gucy2f* genes without inactivating mutation are illustrated by a dark blue bar adjacent to it. The different types and color sensitivities of photoreceptor single cones are indicated by the colored dots and rod monochromacy is shown by a white circle. The presence or absence of intact ORFs encoding GCAPs is given by the colored bars in the dark gray circles. *guca1a* is given in light red, *guca1b* in light blue and *guca1c* is shown in yellow. Sequences that have only a single mutation, which might be derived from sequencing errors, are given by broken lines. **(C)** Evolution of opsins, GCs and GCAPs in major carnivora, bat and xenarthra orders. Major linages are shown. Opsins are given by circles, GCs by triangles and GCAPs by squares. The most likely ancestral situation at every major branch point is given. Numbers in parenthesis represent the species still displaying this gene content (first number) and the total number of species analyzed (second number).

With more than 280 different species, carnivores represent a large order within the mammalian class (van Valkenburgh and Wayne, 2010). Their members are very diverse including cat-like feliformes as well as dog-like caniformes in which dogs, bears, mustelids, raccoons and seals are combined. While dogs and cat-like species are true carnivores, most other members are rather omnivores having adapted to a broader range of diets (van Valkenburgh and Wayne, 2010). True carnivores are often diurnal, whereas many omnivores are rather nocturnal. All these lifestyle adaptations have also impacted the visual systems of these animals (Bowmaker and Hunt, 2006). In a previous study, we have shown that members of the weasel *(mustelidae)* family, which includes badgers, otters and martens, have lost their second visual guanylyl cyclase (Gesemann and Neuhauss, 2020). Interestingly, this also independently occurred in another carnivora family, namely the mongoose *(Herpestidae)* which combines more than 30 different species. However, while some *mustelidae* and *herpestidae* species are nocturnal and are less dependent on vision such as the honey badgers *Mellivora capensis*, others like the banded mongoose *Mungos mungo* or the meerkat *Suricata suricatta* are highly visual diurnal dichromatic animals (Wilson et al., 2009). Interestingly, this seems to influence the number of active GCAPs in these animals. While the honey badger has only persevered two GCAPs, the banded mongoose and also the sea otter have kept three GCAPs (Figure 6). Such a reduction in GCAPs can also observed in some nocturnal carnivores that have kept two GCs. While *Felidae* (cats) are often diurnal or crepuscular and display 3 GCAPs, members of the nocturnal hyena family lack an intact *guca1c* gene (Figure 6).

The most striking adaptions in phototransduction genes are observed in the *Procyonidae* family. It was reported that some *Procyonidae*, which includes raccoons and kinkajous, are highly specialized, nocturnal, monochromatic animals (Jacobs and Deegan, 1992). When we analyzed the opsin sequences of these species we found an intact open reading frame for the kinkajou *opsin1sw1* transcript (Supplementary Figure S2). The only abnormality that could be seen was at the 3′ splice site following exon3, where the regular splice site consensus at the exon/intron boundary was GC instead of GT. However, we found such a change quite frequently in our gene studies, suggesting that either splicing after GC can occur or that RNA editing can change the GC to a GT in these transcripts. Remarkably, the raccoon *Procyon lotor* as well as the kinkajou *Potos flavus* have reduced the guanylyl cyclase genes and their activating proteins to a minimum in preserving only one member each (Figure 6). This suggests that rod and cone monochromatic or dichromatic vision works well with only one protein of each subtype and that higher combinatorial possibilities are only beneficial for animals having a higher number of cone photoreceptors or are active under diurnal conditions.

We found such a striking reduction of *opsin*, *gucy* and *guca* genes also in the suborder of *Xenarthra*. Among xenarthrans are species like armadillos, sloths and anteaters, that have been shown to have only limited vision (Emerling and Springer, 2015). While armadillos are rod monochromats, sloths and anteaters are rod/cone monochromats. Interestingly, the diurnal anteater *Myrmecophaga tridactyla* shows the highest reduction rate in *gucy* and *guca* genes, leaving only one member of each protein family intact. While the rod monochromatic armadillo *Dasypus novemcinctus* might have inactivated all but one of the *gucy* and *guca* genes (for *guca1b* we however found only one slight modification), in the sloth *Choloepus hoffmanni* we still found an intact *guca1b* gene, suggesting that rods and cones might still use a separate activating protein.

### Gucy and *guca* gene reduction can occur in visually impaired echolocating microbats

Bats are a surprisingly large and diverse mammalian order with more than 1,300 different species (Wilson and Reeder, 2005). With the exception of Antarctica, bats are present on all continents and even on isolated islands. Moreover, bats not only vary greatly in size, ranging from wingspans less than 15 cm in hog-nosed bats to more than 1.7 m in flying foxes, but also in their preferred diet being either frugivores, nectarivores, insectivores or in rare cases also praying on small mammals or using blood as their primary diet (Wilson and Reeder, 2005). In a previous report (Gesemann and Neuhauss, 2020) we have shown that megabats, which still strongly rely on their well-developed, rod dominated, visual system (Wang et al., 2004; Gutierrez et al., 2018), have conserved both visual GCs. In contrast to microbats, with often only rudimentary eyes, have often reduced their GC complement. This might be explained by the well-developed LDC (low duty cycle) or HDC (high duty cycle) echolocation system in microbats that compensates for the degeneration of the visual system (Simões et al., 2018). Such a system is absent in megabats, keeping visual input a viable requirement. The strongest reduction of phototransduction cascade calcium feedback genes is seen in the strictly nocturnal monochromatic ghost-faced bat *Mormoops blainvillei* (Lancaster and Kalko, 1996), which has reduced visual GCs and GCAPs to one variant each. Remarkably, the phylogenetically closely related Parnell’s mustached bat *Pteronotus parnellii* still keeps an open reading frame for *guca1b* (Supplementary Table S1). This might be explained by the fact that in contrast to *Mormoops blainvillei, Pteronotus parnellii* has still dichromatic vision (Gutierrez et al., 2018). A similar situation is observed in the little tube-nosed bat *Murina aurata*. This dichromatic nocturnal species belongs to the family of *Vespertilionidae* (common bats) and has open reading frames for *guca1a* and *guca1b*. This is true for all analyzed *Vespertilionidae* species with the exception of the Natal long-fingered bat *Miniopterus natalensis*, in which the second active GCAP is *guca1c* instead of *guca1b*. The combination of *guca1a* and *guca1c* is not only observed in *Miniopterus natalensis* but also in the greater bulldog bat *Noctilio leporinus* that belongs to the family of *Noctilionidae*. Interestingly, independent of its phylogenetic relation, crepuscular bat species have the highest amount of combinatorial possibilities for guanylyl cyclases and their activating proteins, ranging from three (1×3) in the Mexican free-tailed bat *Tadarida brasiliensis*, to six (2×3) in the large flying fox *Pteropus vampyrus* and the Greater false vampire bat *Megaderma lyra*. While the monochromatic *Megaderma lyra* and dichromatic *Tadarida brasiliensis* use echolocation as well as visual input for hunting, the frugivorus and nectarivoros *Pteropus vampyrus* rely solely on their well developed eyesight for foraging (Figure 6). Overall we have analyzed the GCAP integrity in 21 different bat species and there seems to be a strong correlation between a well developed visual system and the preservation of GCAP genes (Supplementary Table S1), once again suggesting that a dual system of phototransduction offers benefits for highly visual animals.

### Monochromatic aquatic mammals benefit from a redundant Set of *gucy* and *guca* genes

Our previous analysis has shown that regressive evolution of the visual system strongly influences the number of active phototransduction gene ohnologs. Many visually impaired, underground living species have not only reduced their spectral sensitivity but have also reduced ohnologous genes important in the calcium feedback system of phototransduction. Analogously, exclusively nocturnal animals have used a similar adaptation, even if their night vision might still be excellent. However, with one exception, the reduction to a minimal set of one *gucy* on one *guca* gene is only observed in monochromatic animals, either being rod or rod/cone monochromats. To test if monochromacy is indeed stimulating inactivation of GCs and their activating proteins we have analyzed the situation in aquatic mammals that have been shown to be primarily monochromatic (Levenson et al., 2006; Meredith et al., 2013).

**Figure 7:**
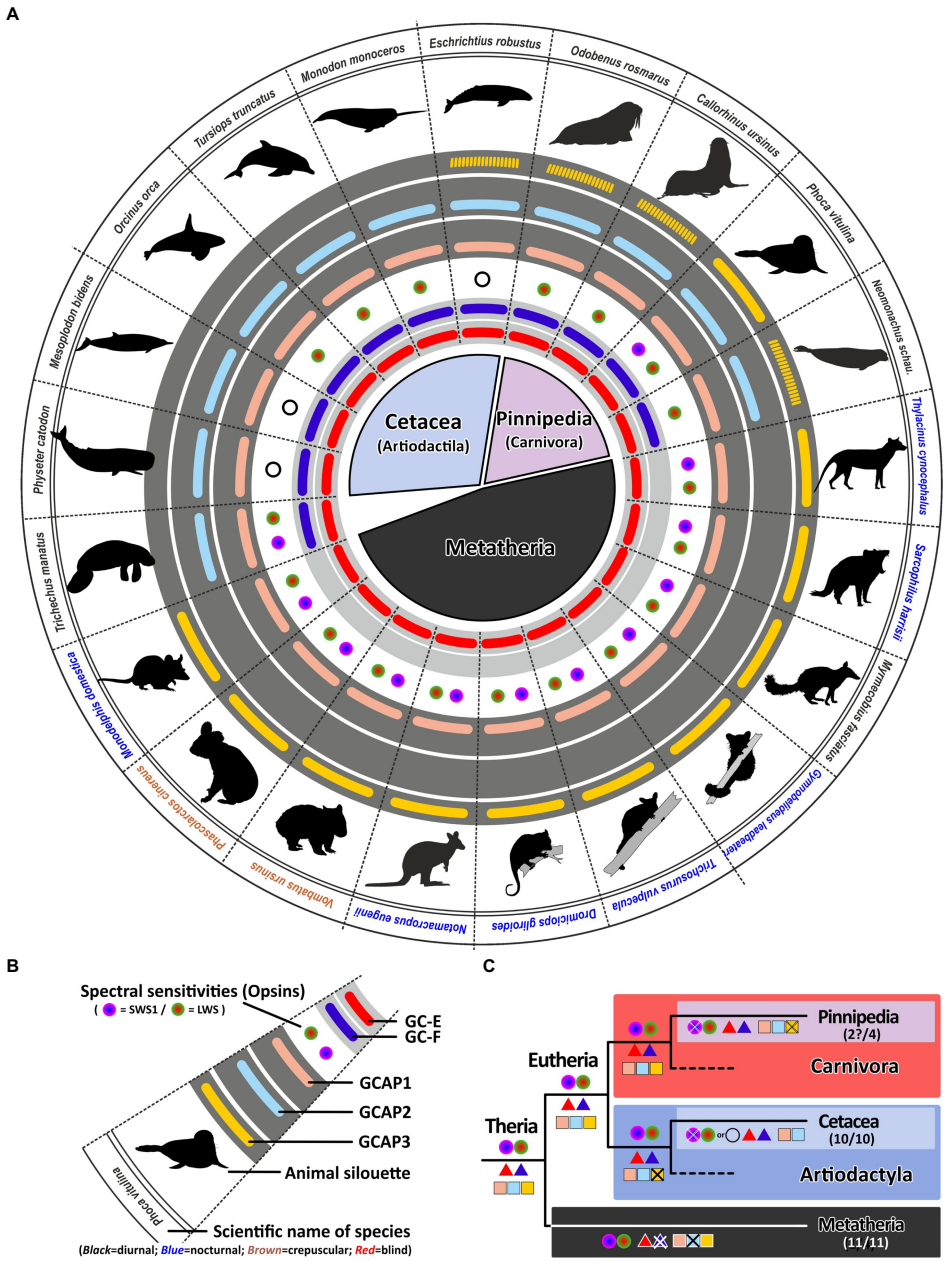
Monochromacy in aquatic mammals is not reflected in a reduction of genes involved in the calcium feedback system of phototransduction. **(A)** Expression of opsins, GCs and GCAPs in aquatic mammals and marsupials. In the center, major orders and suborders are given. *Cetaceans* (whale and dolphins) are highlighted in light blue, pinnipeds in pink and marsupials in black. **(B)** Explanation of symbols used in **(A)**. In the center, a GC-E cladogram of aquatic mammals such as seals (light blue and twilight blue) as well as marsupials (light gray) is given. Pictograms for each chosen representative are shown and scientific names are given. Names of diurnal species are written in black, crepuscular species in brown and nocturnal species in blue. The presence of an open reading frame for transcripts encoding GC-E are shown as a red bar in innermost light gray circle and *gucy2f* genes without inactivating mutation are illustrated by a dark blue bar adjacent to it. The different types and color sensitivities of photoreceptor single cones are indicated by the colored dots and rod monochromacy is shown by a white circle. The presence or absence of intact transcripts coding for GCAPs is given by the colored bars in the dark gray circles. *guca1a* is given in light red, *guca1b* in light blue and *guca1c* is shown in yellow. Sequences that have only a single mutation, which might be derived from sequencing errors, are given by broken lines. **(C)** Evolution of opsins, GCs and GCAPs in major aquatic and metatherian orders. Major linages are shown. Opsins are given by circles, GCs by triangles and GCAPs by squares. The most likely ancestral situation at every major branch point is given. Numbers in parenthesis represent the species still displaying this gene content (first number) and the total number of species analyzed (second number). Note that rod monochromacy in aquatic mammals is not linked to a reduction of *gucy* and *guca* genes and marsupials have reduced their complement to one visual GC and two GCAPs.

Surprisingly, for all analyzed aquatic mammals we found open reading frames for both visual guanylyl cyclase genes (Figure 7), independent of them belonging to the order of whales or dolphins (*Cetaceans*), or to the clade of seals (*Pinnipeds*). Even more unexpectedly, neither species has less than two GCAPs. However, *guca1c*, seems to be under strong purifying selection being absent or pseudogenized in almost all analyzed species. The fact that aquatic mammals have kept two active GCAPs is particularly striking for deep diving whales such as the sperm whale *Physeter catodon*, the gray whale *Eschrichtius robustus* or the Sowerby’s beaked whale *Mesoplodon bidens*, which are rod monochromats (Meredith et al., 2013). At first glimpse no obvious use for two independent sets of phototransduction genes exists. However, rod monochromatic bowhead whales (*Balaena mysticetus*) have preserved cone somata as well as cone bipolar cells, maintaining the possibility of an alternative photoreceptor signaling pathway (Schweikert et al., 2016). Therefore multichannel rod-based signaling enhancing dark-adapted vision could be possible in a variety of aquatic mammals and the fact that guanylyl cyclases and their activating proteins still exist in duplicates makes such a scenario quite likely.

While such adaptations have not been demonstrated in *pinnipeds*, other findings might explain the higher GC/GCAP preservation. Some studies have suggested that at least some seals still can discriminate colors (Oppermann et al., 2016). When we analyzed the opsin sequences of different *pinnipeds*, we surprisingly found an open reading frame for *opsin1sw* in the walrus *Odobenus rosmarus* (Supplementary Table S2; Supplementary Figure S3B). Also for the harbor seal *Phoca vitulina*, a nearly intact coding sequence was identified (Supplement Figure S3C), with only one bp deletion in exon 3. Whether this mutation is real or rather resulting from poor sequencing quality will certainly be determined by future database updates.

In contrast to *Cetaceans* and most *Pinnipeds*, the aquatic West Indian manatee *Trichechus manatus* is still dichromatic (Newman and Robinson, 2006). Remarkably, the same 2/2 combination of *gucy* and *guca* genes is present in sea cows, suggesting that the aquatic lifestyle somehow benefits from a dual system of phototransduction genes independent whether the species is a rod monochromat, a rod/cone monochromat or a dichromat. In contrast to whales, dolphins and seals, manatees are herbivores living in shallow coastal waters being exposed to a broad spectrum of light. Having dichromatic vision may help to locate appropriate food sources under photopic light conditions making the manatee visual system more comparable to that of terrestrial mammals. Therefore, the preservation of a dual system for phototransduction in the case of the manatee might be beneficial.

### Convergent evolution of marsupials and eutherians is not directly reflected in *gucy* and *guca* gene inactivations

Marsupials and eutherians have split some 140 million years ago evolving independently on different continents. While the clade of eutherians consists of more than 6,000 different species, the marsupial clade comprises only around 330 extant species (Deakin and O’Neill, 2020). However, convergent evolution of these two clades has resulted in species displaying similar traits and occupying comparable ecological niches (Mitchell et al., 2014). There are for example, underground living marsupial moles and there was a now extinct dog-like Tasmanian wolf/tiger. Moreover, there are nocturnal as well as diurnal and crepuscular marsupials. As for eutherians it has been shown that marsupials are in general dichromatic, however, in contrast to eutherians which may have one or two active visual GCs, marsupials have only one (Gesemann and Neuhauss, 2020). We have now confirmed this in all sequenced marsupials, never being able to identify a second visual guanylyl cyclase (Figure 7). We found for all marsupials two GCAPs, but counterintuitively these are not *guca1a* and *guca1b*, but *guca1a* and *guca1c*, suggesting that *guca1b* was already lost in an early marsupial ancestor. Unfortunately, there is currently no genomic information for visually impaired marsupials, such as the marsupial mole, leaving the question unanswered whether regressive evolution also occurs for marsupial GCAPs. While we did always see the same opsin*/gucy/guca* situation in all marsupials independent of whether they are diurnal, nocturnal or crepuscular, neither of these species has impaired vision and all are still cone dichromats.

## Conclusion

**Figure 8:**
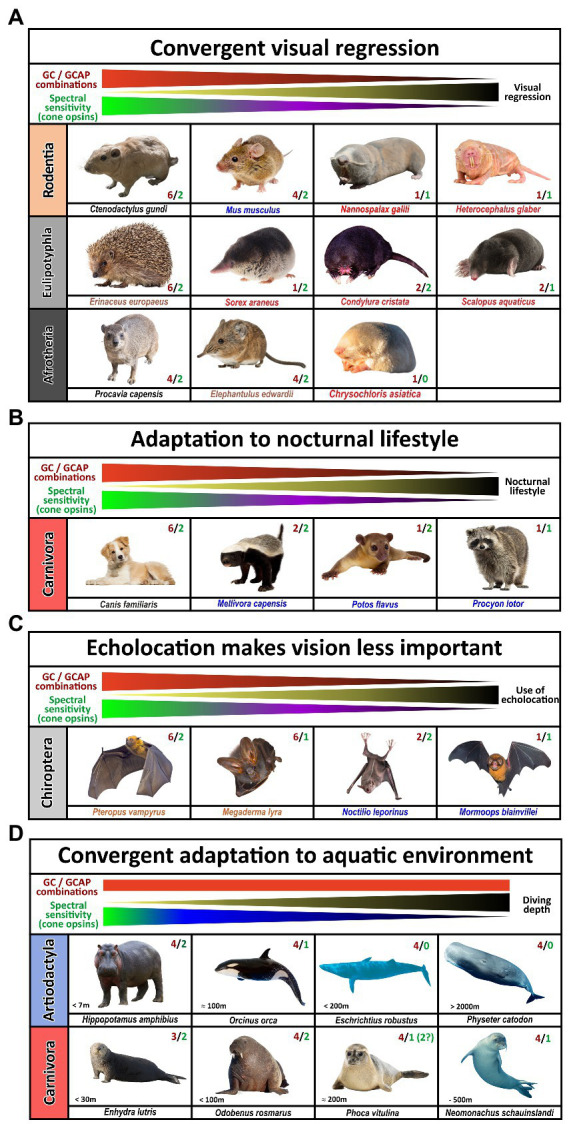
Influence of nocturnal and diurnal lifestyle and subterranean habitats on the evolution of guanylyl cyclase and guanylyl cyclase activating proteins in mammals. The name of the species listed is indicated. Red and green numbers specify the number of functional cone opsins (green) and GC/GCAP combinations (red). **(A)** Convergent visual regression. Visual regression occurred independently in animals of different orders. Species of the rodent, insectivore and afrotheria orders show animals that highly resemble each other and have evolved and adapted to underground life in similar but independent ways. While diurnal or crepuscular species still strongly depend on vision and are functional dichromats with multiple GCs and GCAPs, visually impaired or nearly blind species are often monochromatic and have a greatly reduced complement of GCs and GCAPs. **(B)** Adaptation to nocturnality in carnivores. Several nocturnal carnivores have reduced their opsin as well as their GC and GCAP gene content. While the honey badger *Mellivora capensis* still has dichromatic vision, members of the Procyonidae order are on their way to monochromatic vision. Moreover, Procyonidae have reduced their GC/GCAP content to one member each. **(C)** Echolocation makes vision less important. Nocturnality and echolocation have caused strong adaptation to the visual system of bats. While non-echolocating species such as the frugivorous *Pteropus vampyrus* still rely on vision for foraging, echolocating specialists such as the *Mormoops blainvillei* have greatly reduced their visual dependence. This goes hand in hand with a reduction of functional opsins as well as GCs and GCAP proteins. **(D)** Adaptation to aquatic environments. While aquatic animals often become either rod/cone or rod monochromats, they do not reduce the GC/GCAP complement accordingly. This might be due to the preservation of alternate phototransduction pathways that could enhance dim light vision (for whales and dolphins) or due to the fact that seals are not true monochromats.

**Figure 9:**
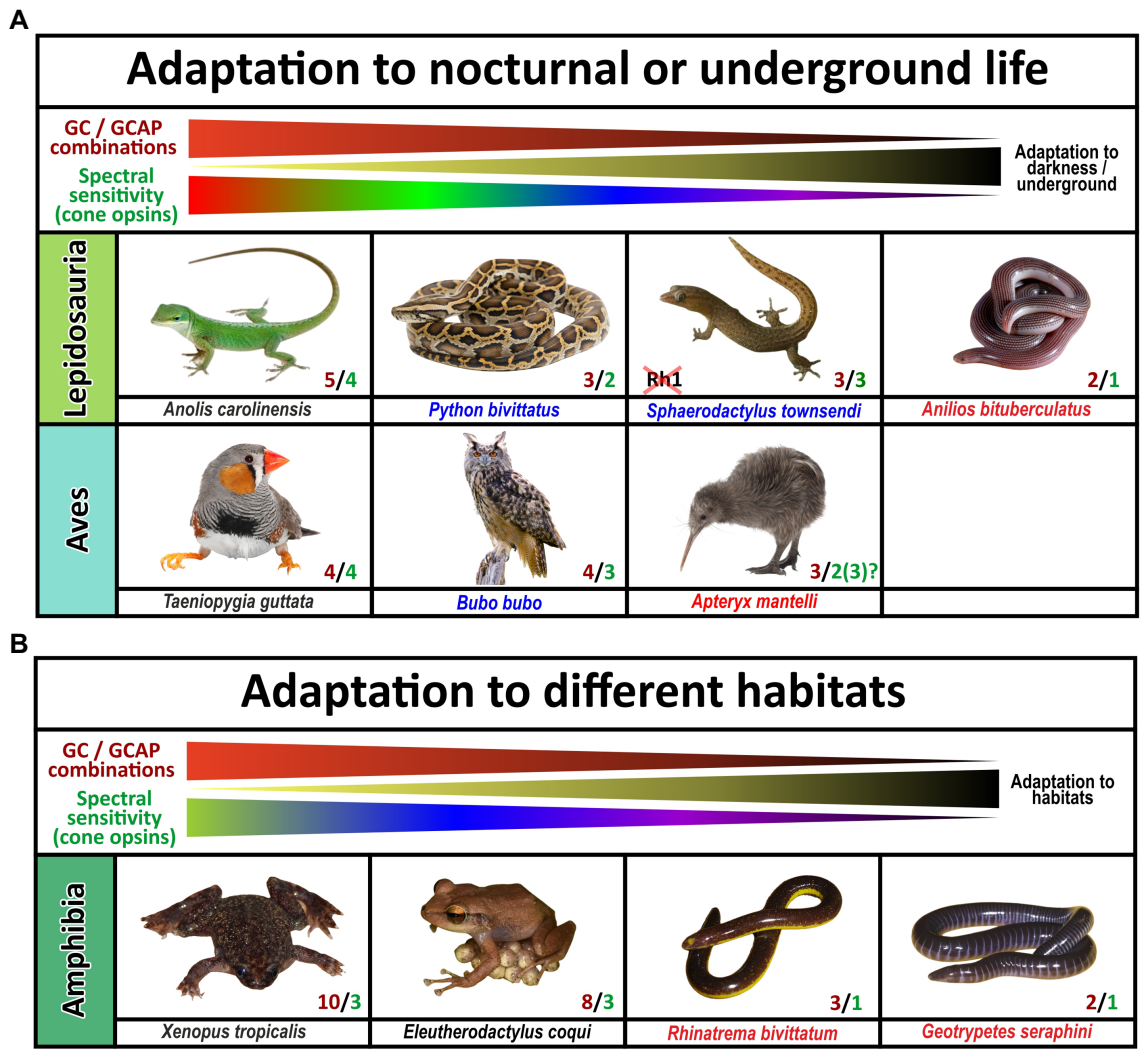
Influence of diurnal, nocturnal, subterranean or changing lifestyles on the evolution of guanylyl cyclase and guanylyl cyclase activating proteins in sauropsids and amphibians. The name of the species listed is indicated. Red and green numbers specify the number of functional cone opsins (green) and GC/GCAP combinations (red). **(A)** Adaptation to nocturnal or underground life in sauropsids. While burrowing, underground living snakes no longer depend on visual input and are cone monochromats, nocturnal predatory species are still di- or trichromatic and diurnal species are often tetrachromats. The reduction of functional visual pigments often correlates with a reduction in GC/GCAP gene content. Note that geckos have inactivated the Rh1 gene usually present in rods, but their cone photoreceptors rather show a rod-like morphology. Also noteworthy is the fact that kiwis as nocturnal birds with poor vision show the beginnings of a reduction of opsins and GCAPs, suggesting that kiwis rather rely on other senses than vision. **(B)** Adaptation to different habitats in amphibians. Frogs and toads show the highest numbers of combinatorial GC/GCAP possibilities, despite having only trichromatic vision. However, as frogs and toads often switch from a larval to an adult form that have different lifestyles and habitats; visual requirements during these phases might be different. These changes might explain the more complex GC/GCAP pattern. Species that do not switch between larval and adult stages or are mainly subterranean show a reduced opsin and GC/GCAP gene content. This is true for the common coqui *Eleutherodactylus coqui* whose offspring do not have a larval stage. Moreover, monochromatic caecilians, which do no longer rely on vision have few possible GC/GCAP combinations.

The visual system of extant tetrapods is a highly adaptive light sensing structure, working from detecting only light/dark differences in fossorial, subterranean living moles towards discriminating a wide range of colors in highly visual lizards, amphibians and primates. While dim light scotopic vision is mediated by rod photoreceptors, photopic color vision is based on different spectral sensitivities of up to four different types of single cones. Visually impaired species have often reduced their photoreceptor subtypes to either rod vision only or rod/cone monochromacy, whereas trichromatic or even tetrachromatic vision can be observed in primates and many sauropsida species. A reduction in spectral sensitivity is also apparent in many nocturnal terrestrial species or aquatic mammals (for summary see Figures 8, 9).

Signal propagation from activated rod or cone photopigments occurs *via* a dual system of phototransduction cascade proteins that differ slightly in rods and cones (Figure 1), with some components being shared, whereas others being exclusively rod or cone specific. In this study we have focused on visual GCs and GCAPs that control the calcium feedback system of phototransduction and are neither rod nor cone specifically expressed. While two *gucy* were active in eutherian and amphibian ancestors, the common ancestor of birds, turtles, crocodiles and lizards has pseudogenized the second visual guanylyl cyclase causing all extent sauropsida species to rely on only one *gucy* gene in cone as well as rod photoreceptors (Supplementary Figure S4). Interestingly, highly visual sauropsida species have compensated the loss of the second visual GC by preserving up to five GCAPs, whereas the mammalian ancestor has inactivated two of these. This leaves eutherians with six combinatorial possibilities (2 *gucy* and 3 *guca* genes) and sauropsids with five (1 *gucy* and 5 *guca* genes). In nearly blind species or species with reduced visual capabilities, we see a strong reduction of these combinatorial possibilities, in the most extreme cases to only one protein each. Such an event has occurred in several clades independently as we found such a situation in rodents, bats, *Procyonidae* (raccoons), *Xenarthra* (anteaters and armadillos) and insectivores (Figure 8). A strong reduction can also be seen in blind worm-like burrowing snakes and underground living limbless caecilians (an order of amphibians), even if the reduction of the gene complement is not or not yet complete (Figure 9). A strong reduction in phototransduction calcium feedback proteins is also seen in marsupials, independent on their lifestyle, where only one cyclase and two activating proteins are found. Interestingly, we found that aquatic mammals, which are almost exclusively rod or rod/cone monochromats, have kept two *gucy* and two *guca* genes. This finding together with the fact that some whales have retained cone somata and cone bipolar cells suggests that keeping an interacting dual system of these enzymes intact might be beneficial to enhance the visual performance.

## Data availability statement

The original contributions presented in the study are included in the article/Supplementary material, further inquiries can be directed to the corresponding author.

## Author contributions

MG: conceptualization, assembled sequences, analysis, and writing. SN: conceptualization and editing. All authors contributed to the article and approved the submitted version.

## Funding

This work was supported by the Swiss National Science Foundation (310030_204648).

## Conflict of interest

The authors declare that the research was conducted in the absence of any commercial or financial relationships that could be construed as a potential conflict of interest.

## Publisher’s note

All claims expressed in this article are solely those of the authors and do not necessarily represent those of their affiliated organizations, or those of the publisher, the editors and the reviewers. Any product that may be evaluated in this article, or claim that may be made by its manufacturer, is not guaranteed or endorsed by the publisher.

## Footnotes

^1^ http://www.ncbi.nlm.nih.gov

^2^ http://www.ensembl.org/index.html

^3^ http://hollywood.mit.edu/GENSCAN.html

^4^ https://ngphylogeny.fr

^5^ https://itol.embl.de

^6^ http://syntenydb.uoregon.edu/synteny_db/

^7^ https://www.shutterstock.com
