# Supplementary material for: Evolution of visual guanylyl cyclases and their activating proteins with respect to clade and species-specific visual system adaptation
Source: Front Mol Neurosci. 2023 Mar 16;16:1131093. doi: 10.3389/fnmol.2023.1131093 (PMC10061024; doi:10.3389/fnmol.2023.1131093)
Supplement: Supplementary file 1 [file Data_Sheet_1.pdf]

	Species	**SWS1 (violet/UV)** / Accession # and localization	**LWS (green/red)** / Accession # and localization
	Homo sapiens	NC_000007.14:128772540-128775781	**(LWS2)**
**Glires (Rodents & Rabbits)**	*Mus musculus*	NC_000072.7:29377941-29380414	NC_000086.8:73171113-73194262
	Zapus hudsonius	PVHP010009510.1:2186-5561	PVHP010021328.1:7258-20836
	Microtus arvalis	VIIT010001838.1:74818-77487	VIIT010017770.1:16499-31827
	Nannospalax galili	NW_008349073.1:1759169-1762514 **(at 1761879 1bp insert)**	NW_008334861.1:3630924-3644873
	Dipodomys stephensi	PVHN010002806.1:20164-22723	PVHN010004454.1:21339-37206
	Castor canadensis	NW_017869518.1:630041-632814 **(at 632667 1bp deletion)**	NW_017876104.1:30422-44755
	Glis glis	PVJS01027489.1:14419-17268 **(pseudogene / multiple mut.)**	PVJS01098558.1 (2504..ex1) / PVJS01086560.1 (1161..ex4&5)
	Xerus inauris	PVHX01008923.1:10388-13028	PVHX01030306.1:8953-13726 (Ex.1&2) / PVHX01035654.1:4433-11177 (Ex.3-6)
	Ctenodactylus gundi	PVKB01000679.1:59848-62377	PVKB01003595.1:64940-75331
	Hystrix cristata	PVJO010024223.1:23150-24343 (Ex1-3) &<br>PVJO010015021.1:438-1250 (Ex4&5) **pseudogene multiple mut.!!**	PVJO010009464.1:7207-15305
	Fukomys damarensis	NW_022900916.1:38272856-38276049	NW_022900951.1:127571802-127578074
	Heterocephalus glaber	NW_004624783.1:7412825-7416095	**pseudogene (fragments only…)**
	Oryctolagus cuniculus	NC_067380.1:157741014-157743690	NW_026259253.1:293170-303382
	Lepus americanus	PVJM010001051.1:36077-39155	PVJM010000542.1:69108-80534
**Eulipotyphla**	*Sorex araneus*	NW_004545887.1:18938625-18942189	NW_004545877.1:6991459-6999901
	Erinaceus europaeus	NW_006804217.1:2326524-2335430	NW_006804684.1:58940-66508
	Uropsilus gracilis	PVHY01012114.1:30393-34614	PVHY01031338.1:4268-12610
	Condylura cristata	NW_004567106.1:59052297-59055223	NW_004567158.1:527473-535412
	Scalopus aquaticus	PVIJ01001752.1:142050-145267	**PVIJ01013172.1:34007-42240 (several base pair deletions)**
**Afrotheria**	*Loxodonta africana*	NW_003573425.1:98284584-98287947	NW_003573540.1:1873034-1884492 (last exon missing)
	Procavia capensis	PVIO03000025.1:21145719-21149473	PVIO03000001.1:5715443-5727846
	Chrysochloris asiatica	**NW_006408559.1:1233832-1237111 (2bp deletion in exon4)**	**NW_006408963.1:1085265-1097669 (several bp deletions)**
	Trichechus manatus	NW_004444033.1:3456032-3459358	NW_004444245.1:839432-853960
**Carnivora**	*Procyon lotor*	**JAABKC010000702.1:387180-383748 (multiple deletions / no orf)**	JAABKC010001793.1:38900-49850
	Potos flavus	JAABKN010000032.1:8404891-8408264	JAABKN010001291.1:41334-52218
	Mellivora capensis	PISX010001485.1:183861-187410	PISX010007790.1:54150-66496
	Enhydra lutris	NW_019154111.1:13651559-13654929	NW_019154180.1:3471621-3483604
	Canis familiaris	NC_051818.1:7631487-7634386	NC_051843.1:125116233-125129103
	Mungos mungo	PISW01000023.1:224551-227692	PISW01012278.1:19607-32167
	Hyaena hyaena	NW_024080640.1:2121032-2124159	NW_024081437.1:830132-842692
	Felis catus	NC_058369.1:145093279-145096451	NC_058386.1:126133374-126145677
	Neomonachus schau.	**NC_058414.1:82676041-82679630 (11bp deletion in exon4)**	NC_058419.1:1421015-1433420
	Phoca vitulina	NW_022589707.1:16502998-16506628 (1bp deletion in exon3)	NW_022589802.1:342247-355681
	Callorhinus ursinus	**NW_020323812.1:1401962-1405383 (4bp insertion in exon1)**	NW_020313389.1:3559856-3572354
	Odobenus rosmarus	NW_004450341.1:4706489-4709872	NW_004450679.1:831355-844333
	Cervus elaphus	NC_057832.1:98985640-98988632	NC_057848.1:27250181-27262677
**Artiodactyla**	*Sus scrofa*	NC_010460.4:19836468-19839425	NC_010461.5:124816040-124828115
	Bos taurus	NC_037331.1:92745939-92748919	NC_037357.1:37321404-37333520
	Eschrichtius robustus	**NIPP01001646.1:210872-213914 (frame shift mutations in exon 1&2)**	**NIPP01003433.1:4293-15108 (Mut. In ATG startcodon; 1bp del. In Ex2)**
	Monodon monoceros	NW_021703776.1:81863093-81866062 (two in frame deletions)	NW_021703779.1:127331851-127342659
	Tursiops truncatus	**NC_047042.1:82181626-82184656 (frame shift mut. in ex1; 1bp del.)**	NC_047055.1:1351892-1362379
	Orcinus orca	NC_064567.1:23273457-23276516 (2bp del. In ex4)	NC_064580.1:131110680-131120460
	Mesoplodon bidens	**PVJJ010003519.1:5699-8745 (frame shift mut. in ex1; 1bp del.)**	**PVJJ010000096.1:20284-31169 (frame shift mut. in ex2)**
	Physeter catodon	**NC_041218.1:23771083-23780940 (1bp insertion in ex1)**	**NC_041234.1:120915389-120925031 (frame shift mut in ex2; no reg. stop)**
Perissodactyla	*Equus caballus*	NC_009147.3:83775344-83778439	NC_009175.3:126636000-126647085
**Chioptera**	*Pteropus vampyrus*	NW_011888814.1:8441705-8446056	NW_011889056.1:785577-796199
	Megaderma lyra	**PVJL010004670.1:26941-29820 (deletions in exons 1 (1bp); exon 4 (2x 1bp)**	PVJL010004545.1:41062-51992
	Miniopterus natalensis	NW_015504548.1:6313323-6317201	NW_015504600.1:894423-907982
	Noctilio leporinus	PVIW01007103.1:62330-66170	PVIW01010093.1:11874-23388
	Hipposideros armiger	**NW_017731447.1:3377875-3379945 (partial; multiple bp deletions)**	NW_017731769.1:1449930-1463456
	Tadarida brasiliensis	PVIG010001018.1:3497-6512	PVIG010016147.1:3751-15623
	Murina aurata	PVJC01022415.1;12046-15305	PVJC01075781.1:473-4349 (Ex 2&3)<br>PVJC01043808.1:11398-13109 (Ex 5&6)
	Mormoops blainvillei	**PVJD01003679.1:10890-14301 (ins/del Ex1; 13bp del Ex2)**	PVJD01010105.1:25429-36824
**Xenarthra**	*Dasypus novemcinctus*	**NW_004481312.1:74175-78134 / pseudogene (multiple ins/del)**	**NW_004501067.1:457593-466680 / pseudogene (4times 1bp deletion)**
	Myrmecophaga tridactyla	**Fragments on PVIY010000962.1:154603-155853 / pseudogene**	PVIY010020782.1:109-11424
	Choloepus hoffmanni	**KN178083.1:38481-40355 / Pseudogene (multiple ins/del)**	KN194897.1:160018-172003
**Metatheria**	*Monodelphis domestica*	NC_008808.1:188614210-188617858	NC_008809.1:14659882-14669206 (partial, Ex 2-5)
	Phascolarctos cinereus	NW_018344048.1:7194442-7199064	NW_018344262.1:318967-335015
	Vombatus ursinus	NW_020954617.1:5489277-5493867	NW_020954802.1:1018456-1033450
	Notamacropus eugenii	GL127888.1:127616-131669	GL058643.1:30362-45554
	Dromiciops gliroides	NC_057865.1:117787677-117792205	NC_057867.1:31217516-31233371
	Trichosurus vulpecula	NC_050577.1:119331316-119332985	NW_023494377.1:8242220-8258742
	Gymnobelideus leadbeateri	WOXC01010363.1:68508-72429	WOXC01003271.1:161373-177871
	Myrmecobius fasciatus	JAJPUD010004067.1:7870-11653	JAJPUD010002526.1:154957-167014
	Sarcophilus harrisii	NC_045430.1:176946589-176950720	NC_045432.1:12415467-12426848
	Thylacinus cynocephalus	CM040578.1:181394719-181398428	CM040580.1:12045500-12054193 (Ex 1-5)<br>CM040574.1:530451767-530451877 (Ex 6)

Green: Sense orientation / Brown: Antisense orientation / Red: Likely pseudogenized
