# Supplementary material for: Evolution of visual guanylyl cyclases and their activating proteins with respect to clade and species-specific visual system adaptation
Source: Front Mol Neurosci. 2023 Mar 16;16:1131093. doi: 10.3389/fnmol.2023.1131093 (PMC10061024; doi:10.3389/fnmol.2023.1131093)
Supplement: Supplementary file 2 [file Data_Sheet_2.PDF]

		Species	Gene name	Exon1 from ATG	Intron 1	Exon 2	Intron 2	Exon 3	Intron 3	Exon 4 (till stop)	Accession Number (Gene sequence including introns)
Primates	Haplorrhini	***Homo sapiens* (Human)**	Guca1A_hsa	201	4465	150	372	94	347	161	NC_000006.12: 42173614-42179403
			Guca1B_hsa	207	5882	150	2784	118	737	128	NC_000006.12: 42184815-42194820
			Guca1C_hsa	204	32973	150	4221	88	7917	188	NC_000003.12: 108908022-108953762
		Macaca mulatta (Rhesus macaque)	Guca1A_mmul	201	3174	150	371	94	334	164	NC_041757.1: 127180188-127184675
			Guca1B_mmul	207	6249	150	3063	118	1369	128	NC_041757.1: 127163504-127174787
			Guca1C_mmul	204	31835	150	4467	88	9057	194	NC_041755.1: 167237252-167283246
		Chlorocebus sabaeus (Green monkey)	Guca1A_csa	201	3503	150	370	94	334	164	NW_023666044.1: 42196376-42201191
			Guca1B_csa	207	6250	150	2837	118	1063	128	NW_023666044.1: 42206323-42217075
			Guca1C_csa	204	32687	150	4591	88	9148	194	NW_023666041.1: 94581406-94628467
		Colobus angolensis (Angola colobus)	Guca1A_can	201	5027	150	371	94	323	164	NW_012111475.1: 400302-406631
			Guca1B_can	207	6185 (Ns)	150	2850 (Ns)	118	1051	128	NW_012111475.1: 384155-394843
			Guca1C_can	204	33430 (Ns)	150	4458	88	9087	194	NW_012123820.1: 2080466-2128076
		Cebus imitator (Panamanian white-faced capuchin)	Guca1A_cim	201	5812 (Ns)	150	364	94	335	164	NW_016107512.1: 918629-925748
			Guca1B_cim	207	??	??	??	118	719	128	NW_016107512.1: 930727-945253
			Guca1C_cim	204	23918 (Ns)	150	4635	88	7506	185	NW_016107754.1: 907952-944637
		Ateles hybridus (Brown spider monkey)	Guca1A_ahy	201	5342	150	366	94	336	164 (+1)	CAJZLT010000016.1: 11337229-11343880
			Guca1B_ahy	207	6911	150	5672	118	714	128	CAJZLT010000016.1: 11348793-11362692
			Guca1C_ahy	207	23878	150	4672	88	6903	194	CAJZLT010000048.1: 17504609-17540700
		Carlito syrichta (Philippine tarsier)	Guca1A_csy	201	??	??	??	??	??	164	NW_007249851.1: 663492-672347
			Guca1B_csy	207	8224 (Ns)	150	4065	121	629	128	NW_007249851.1: 644912-658435
			Guca1C_csy	204	24829	150	2875	88	9127	194	NW_007252362.1: 357179-394645
	Strepsirrhini	*Otolemur garnettii* (Northern greater galago)	Guca1A_oga	201	7295 (Ns)	150	357	94	294	164	NW_003852426.1: 12764166-12772720
			Guca1B_oga	207	7550	150	1631	121	716	128	NW_003852426.1: 12776415-12786917
			Guca1C_oga	204	25025 (Ns)	150	4472	88	6616	185	NW_003852526.1: 4558744-4595483
		Mirza coquereli (Coquerel's giant mouse lemur)	Guca1A_mco	201	5451	150	343	94	327	164	PVHQ01029825.1: 3348-10077
			Guca1B_mco	207	??	150	??	121	1076	128	PVHQ01041187.1 (Exon 1) / PVHQ01090256.1 (Exon 2)<br>PVHQ01061161.1 (Exon 3&4)
			Guca1C_mco	204	19600	150	4493	88	6652	194	PVHQ01003786.1: 39027-70407
		Daubentonia madagascariensis (Aye-aye)	Guca1A_dma	201	5366	150	371	94	319	155	JAKDFM010000090.1: 1985103-1991759
			Guca1B_dma	207	5187	150	1459	121	741	128	JAKDFM010000090.1: 1973821-1981812
			Guca1C_dma	204 (+1)	22289	150	4584	88	6574	194	JAKDFM010001104.1: 7462353-7496392
		Tupaia belangeri (Northern treeshrew)	Guca1A_tbe	201	5442	150	282	94	1189 (Ns)	167	NW_006159716.1: 1981152-1988676
			Guca1B_tbe	213	9447 (Ns)	150	2502	121	640	128	NW_006159716.1: 1963274-1976475
			Guca1C_tbe	204	22602 (Ns)	150	1400	88	10704	155	NW_006203439.1: 392125-427427
		Galeopterus variegatus (Sunda flying lemur)	Guca1A_gva	201	5146	150	343	94	304	161	NW_007726563.1: 188198-194596
			Guca1B_gva	207	8394 (Ns)	150	2147 (Ns)	121	680	128	NW_007726563.1: 173085-184911
			Guca1C_gva	204	23777 (Ns)	150	8494	88	9961 (Ns)	194 (-1) /137 ?	NW_007726975.1: 323765-366575
		Pedetes capensis (South African springhare)	Guca1A_pca	201	4961	150	385	94	300	173	VMDO01000105.1: 377228-383491
			Guca1B_pca	207	6389	150	3613	121	959	128	VMDO01000105.1: 361976-373542
			Guca1C_pca	204 (1 Stop / No splice cons)	??	??	??	88 ? (Stops / No splice cons)		188 (2 Stops)	VMDO01009119.1: 34167-63262
	Castorimorpha	***Castor canadensis* (North American beaver)**	Guca1A_cca	186 ? Many mistakes / 1 stop	4800	150	345	94	294	164	NW_017869957.1: 3217585-3218633
			Guca1B_cca	207	4853	144 (-1)	2232	121	971	128	NW_017869957.1: 3204984-3213640
			Guca1C_cca	204 ? No atg / 2 stops	29349	150 (+1) / 1 stop	3808	88 ? No splice consensus	5932	185 / 1 stop	NW_017877369.1: 23493-63207
		Dipodomys ordii (Ord's kangaroo rat)	Guca1A_dor	201	2906	150	1305	94	284	170	NW_012267260.1: 8639658-8644767
			Guca1B_dor	207	1821	150	2366	121	1225	128	NW_012267260.1: 8629016-8635033
			Guca1C_dor	204	9594	150	7769	88	11233 (Ns)	179 (-1) / 278	NW_012267327.1: 3688007-3717224
		***Dipodomys stephensi* (Stephens's kangaroo rat)**	Guca1A_dst	201	3086	150	1312	94	263	170	PVHN010013373.1: 929-6205
			Guca1B_dst	207	1840	150	2269	121	??	128	PVHN010010600.1: 46046-50633 (Exons 1-3) / PVHN010062303.1
			Guca1C_dst	204	??	150	7146	88	??	179 (-1) / 236	PVHN010011244.1: 7190-7394 (Exon 1)
		Perognathus longimembris (Little pocket mouse)	Guca1A_plo	201	2665	150	515	94	440	170	NC_063166.1: 7644240-7648474
			Guca1B_plo	207	1375	150	1431	121	919	128	NC_063166.1: 7636865-7641195
			Guca1C_plo	204	10386	150	6991	88	9628	182 (-1) / 290	NC_063165.1: 38046840-38074576
		***Hystrix cristata* (Crested porcupine)**	Guca1A_hcr	201	3265	150	190	94	208	158	PVJO010006079.1: 75658-79923
			Guca1B_hcr	204	1249	150	1360	121	873	128	PVJO010006079.1: 69026-73110
			Guca1C_hcr	-	-	-	-	88 ?? (no splice cons)	5123	188	PVJO010026174.1: 5926-11324 (Exons 3&4)
		**Fukomys damarensis (Damaraland mole-rat)**	Guca1A_fda	201	3492	150	184	94	207	158	NW_022900932.1: 8290118-8294603
			Guca1B_fda	204	1428	150	128	121		128	NW_022900932.1: 8297277-8300312
			Guca1C_fda	-	-	-	-	-	-	-	
		**Heterocephalus glaber (Naked mole-rat)**	Guca1A_hgl	201	3479	150	181	94	245	158	NW_004624754.1: 17089148-17093655
			Guca1B_hgl	207 (+1)	1642	150	161	109 (+1)	1093	128	NW_004624754.1: 17083523-17087010
			Guca1C_hgl	-	-	-	-	-	-	-	
		Capromys pilorides (Desmarest's hutia)	Guca1A_cpi	201	??	150	168	94	257	158	PVKN010033826.1: 3058-3262 (Exon 1)
			Guca1B_cpi	204	??	150	1305	121	??	128	PVKN010435417.1 (Exon 1) / PVKN010259833.1 (Exons 2&3)
			Guca1C_cpi	-	-	-	-	-	-	-	
		Cavia porcellus (Guinea pig)	Guca1A_cpo	201	3323	150	130	94	226	158	NT_176404.1: 32031028-32035313
			Guca1B_cpo	204	1800	150	173	121	1153	128	NT_176404.1: 32023848-32027576
			Guca1C_cpo	-	-	-	-	-	-	-	

	Species	Gene name	Exon1 from ATG	Intron 1	Exon 2	Intron 2	Exon 3	Intron 3	Exon 4 (till stop)	Accession Number (Gene sequence including introns)
Glires / Hystricomorpha	*Chinchilla lanigera* (Long-tailed chinchilla)	Guca1A_cla	201	4809 (Ns)	150	168	94	257	158	NW_004955437.1: 8376730-8382566
		Guca1B_cla	204	1937	150	1153	121	973	128	NW_004955437.1: 8385887-8390552
		Guca1C_cla	-	-	-	-	-	-	-	
	Ctenodactylus gundi **(Common gundi)**	Guca1A_cgu	201	6070	150	483	94	441	170	PVKB01004176.1: 34310-41918
		Guca1B_cgu	207	1326	150	1711	109	823	128	PVKB01004176.1: 46084-50537
		Guca1C_cgu	204	21255	150	3984	88	5951	188	PVKB01001082.1: 375389-407208
	Ctenomys sociabilis (Social tuco-tuco)	Guca1A_cso	201	??	150	168	94	229	158	PVKA01061277.1 (Exon 1) / PVKA01049973.1 (Exons 2-4)
		Guca1B_cso	204	1945	150	1011	121	769	128	PVKA01049973.1: 7219-11546
		Guca1C_cso	-	-	-	-	-	-	-	
	Cuniculus paca (Lowland paca)	Guca1A_cpa	201	??	150	158	94	239	158	RJWT010282389.1 (Exon 1) / RJWT010232659.1 (Exons 2-4)
		Guca1B_cpa	??	??	150	??	??	??	128	RJWT011074176.1 (Exon 2) / RJWT011700930.1 (Exon 4)
		Guca1C_cpa	??	??	??	??	??	??	??	
	Dasyprocta punctata (Central American agouti)	Guca1A_dpu	201	??	150	176	94	216	158	RJWM01070842.1 (Exon 1) / RJWM01062363.1 (Exons 2-4)
		Guca1B_dpu	204	??	150	197	121	??	??	RJWM01117525.1 (Exon 1) / JWM01223392.1 (Exons 2&3)
		Guca1C_dpu	-	-	-	-	-	-	-	
	Dinomys branickii (Pacarana)	Guca1A_dbr	201	4898	150	168	94	202	158	PVLD010029032.1: 4358-10228
		Guca1B_dbr	204	1480	150	??	??	??	??	PVLD010047072.1 (Exons 1&2)
		Guca1C_dbr	-	-	-	-	-	-	-	
	Erethizon dorsatum (North American porcupine)	Guca1A_edo	201	4159 (Ns)	150	168	94	296	158	ML655366.1: 17671037-17676262
		Guca1B_edo	204	1663	150	926	121	789	128	ML655366.1: 17663469-17667449
		Guca1C_edo	no open reading frame	-	-	-	-	-	-	
	Hydrochoerus hydrochaeris (Capybara)	Guca1A_hhy	201	3143	150	3421	94	??	??	PVLA01016117.1 (Exons 1-3)
		Guca1B_hhy	204	1772	150	128	121	733	128	PVLA01000375.1: 584639-587874
		Guca1C_hhy	no open reading frame	-	-	-	-	-	-	
	Myocastor coypus (Nutria)	Guca1A_mco	201	??	150	160	94	248	158	PVJA010052556.1 (Exon 1) / PVJA010046751.1 (Exons 2-4)
		Guca1B_mco	204	2603	150	2110	121	662	128	PVJA010046751.1: 698-6675
		Guca1C_mco	no open reading frame	-	-	-	-	-	-	
	Octodon degus (Common degu)	Guca1A_ode	201	5251 (Ns)	150	167	94	247	158	NW_004524581.1: 23813433-23819701
		Guca1B_ode	204	3051	150	985	121	742	128	NW_004524581.1: 23803890-23809270
		Guca1C_ode	no open reading frame	-	-	-	-	-	-	
	Petromus typicus (Dassie rat)	Guca1A_pty	201	3879	150	193	94	187	158	PVIR01013651.1: 26958-31819
		Guca1B_pty	204	763	150	1130	121	644	128	PVIR01013651.1: 34743-37882
		Guca1C_pty	no open reading frame	-	-	-	-	-	-	
	Thryonomys swinderianus (Greater cane rat)	Guca1A_tsw	201	??	150	186	94	221	158	PVIC010067844.1: 72-279 (Exon 1)
		Guca1B_tsw	204	757	150	1235	121	??	128	PVIC010046412.1: 4743-8353
		Guca1C_tsw	-	-	-	-	-	-	-	
yomorpha	***Mus musculus*** **(House mouse)**	Guca1A_mmu	201	4521	150	346	94	287	164	NC_000083.7: 47705582-47711344
		Guca1B_mmu	207	3413	150	1721	121	773	128	NC_000083.7: 47696415-47702927
		Guca1C_mmu	-	-	-	-	-	-	-	
	Rattus norvegicus (Brown rat)	Guca1A_rno	201	7605	150	839	94	286	164	NC_051344.1: 13589126-13598464
		Guca1B_rno	207	2605	150	1868	121	1687	128	NC_051344.1: 13600596-13607361
		Guca1C_rno	-	-	-	-	-	-	-	
	Apodemus sylvaticus (Wood mouse)	Guca1A_asy	201	6439 (Ns)	150	785	94	273	164	NC_067480.1: 80020685-80028021
		Guca1B_asy	207	??	150	??	121	1038 (Ns)	128	NC_067480.1: 80030011-80038401
		Guca1C_asy	-	-	-	-	-	-	-	
	Arvicanthis niloticus (African grass rat)	Guca1A_ani	201	5304	150	908	94	280	164	NC_047674.1: 55489686-55496888
		Guca1B_ani	207	3986	150	1811	121	1020	128	NC_047674.1: 55479965-55487387
		Guca1C_ani	-	-	-	-	-	-	-	
	Grammomys surdaster (Woodland thicket rat)	Guca1A_gsu	201	5729	150	788	94	279	170	NW_021604617.1: 8051656-8059066
		Guca1B_gsu	207	4082	150	1806	121	790	128	NW_021604617.1: 8061152-8068435
		Guca1C_gsu	-	-	-	-	-	-	-	
	Lophiomys imhausi (Maned rat)	Guca1A_lim	201	8539	150	595	94	294	170	CAJQZJ010001969.1: 1923147-1933189
		Guca1B_lim	207	4056	150	2039	121	1419	128	CAJQZJ010001969.1: 1912935-1921054
		Guca1C_lim	-	-	-	-	-	-	-	
	Acomys percivali (Percival's spiny mouse)	Guca1A_ape	201	8138 (Ns)	150	859 (Ns)	94	277	164	OU015751.1: 49356018-49365901
		Guca1B_ape	207	4511	150	2065	121	1212	128	OU015751.1: 49344798-49353191
		Guca1C_ape	-	-	-	-	-	-	-	
	Rhombomys opimus (Great gerbil)	Guca1A_rop	201	4224	150	824	94	143	164	REGO01000079.1: 976252-982051
		Guca1B_rop	207	3314	150	??	??	??	128	REGO01000079.1: 984090-990633
		Guca1C_rop	-	-	-	-	-	-	-	
	Peromyscus californicus (California deermouse)	Guca1A_pcal	201	7894	150	1147	94	253	164	NW_026294491.1: 62400377-62410279
		Guca1B_pcal	207 ?	3680	150	2043	121	1117	128	NW_026294491.1: 62390603-62398048
		Guca1C_pcal	-	-	-	-	-	-	-	
	Onychomys torridus (Southern grasshopper mouse)	Guca1A_oto	201	7881	150	834	94	210	164	NC_050460.1: 6804916-6814449
		Guca1B_oto	207	3601	150	1788	121	728	128	NC_050460.1: 6816581-6823303
		Guca1C_oto	-	-	-	-	-	-	-	

Clade	Species	Gene name	Exon1 from ATG	Intron 1	Exon 2	Intron 2	Exon 3	Intron 3	Exon 4 (till stop)	Accession Number (Gene sequence including introns)
M	*Neotoma lepida*	Guca1A_nle	201	7589 (Ns)	150	756 (Ns)	94	240	164	LZPO01089559.1: 388451-397644
	(Desert woodrat)	Guca1B_nle	207	2706	150	2236 (Ns)	121	1155 (Ns)	128	LZPO01089559.1: 379644-386346
		Guca1C_nle	-	-	-	-	-	-	-	
	Microtus montanus	Guca1A_mmo	201	6303 (Ns)	150	904 (Ns)	94	269	164	JAEPQX010002215.1: 321415-329499
	(Montane vole)	Guca1B_mmo	Bad sequence							
		Guca1C_mmo								
	Microtus arvalis	Guca1A_mar	201	8524	150	841	94	269	164	VIIT010000072.1: 363955-374197
	**(Common vole)**	Guca1B_mar	207	4381	150	1632	121	1012	128	VIIT010000072.1: 376333-383963
		Guca1C_mar	-	-	-	-	-	-	-	
	Ellobius lutescens	Guca1A_elu	201	8945 (Ns)	150	737	94	269	164	LOEQ01001064.1: 202810-213369
	(Transcaucasian mole vole)	Guca1B_elu	207	2913 (Ns)	150	1630	121	990	128	LOEQ01001064.1: 215445-221583
		Guca1C_elu	-	-	-	-	-	-	-	
	Mesocricetus auratus	Guca1A_mau	201	6131	150	1222	94	276	164	NW_024429202.1: 6511955-6520197
	(Golden hamster)	Guca1B_mau	207	3372	150	1991	121	858	128	NW_024429202.1: 6522568-6529394
		Guca1C_mau	-	-	-	-	-	-	-	
	Cricetomys gambianus	Guca1A_cga	201	7237	150	822	94	227	161	PVKD010004181.1: 120818-129709
	(Gambian pouched rat)	Guca1B_cga	207	4001	150	1958	121	1017	128	PVKD010004181.1: 111397-118978
		Guca1C_cga	-	-	-	-	-	-	-	
	Typhlomys cinereus	Guca1A_tci	201	6319	150	1478	94	287	158	JALPQD010000199.1: 3099916-3108602
	(Chinese pygmy dormouse)	Guca1B_tci	207	2470	150	2424	121	724	128	JALPQD010000199.1: 3112372-3118595
		Guca1C_tci	-	-	-	-	-	-	-	
	Nannospalax galili	Guca1A_nga	201	7082	150	669	94	304	164	NW_008331144.1: 955955-964618
	**(Blind mole-rat)**	Guca1B_nga	207	??	??	??	121 (1 Stop / +1)	??	128	NW_008331144.1: 967846-973600
		Guca1C_nga	-	-	-	-	-	-	-	
	Zapus hudsonius	Guca1A_zhu	201	4401	150	619	94	654	158	PVHP010024244.1: 4551-10827
	**(Meadow jumping mouse)**	Guca1B_zhu	207	8459	150	1450	115	397	128	PVHP010024244.1: 15668-26573
		Guca1C_zhu	-	-	-	-	-	-	-	
	Aplodontia rufa	Guca1A_aru	201	5592 (Ns)	150	561	94	472	164	PVKS010023312.1: 20660-27893
	(Mountain beaver)	Guca1B_aru	207	4976	150	2269	115	1052	128	PVKS010023312.1: 8099-16995
		Guca1C_aru	204	??	150	11681	88	5768	155	PVKS010011093.1: 52046-52250 (Exon 1)
	Glis glis	Guca1A_ggl	201	6043	150	311	94	694	170	PVJS01026370.1: 2603-10265
	**(European edible dormouse)**	Guca1B_ggl	207	6000	150	1445	121	960	128	PVJS01026370.1: 14348-23358
		Guca1C_ggl	204	??	150	9647	88	??	188 (-1)	PVJS01029141.1: 2017-11904 (Exons 2&3) / PVJS01051467.1 (Exon 4)
	Muscardinus avellanarius	Guca1A_mav	201	??	150	227	94	639	146 ?	PVJB01068181.1 (Exon 1)
	(Hazel dormouse)	Guca1B_mav	207	2897	150	913	121	578 (Ns)	128	PVJB01009887.1: 56000-60993
		Guca1C_mav	204	17992	150	6740	88	9307 (Ns)	188	PVJB01007574.1: 12049-46717
	Marmota marmota	Guca1A_mma	201	5589	150	317	94	488	164	NW_025999982.1: 9857809-9864811
	(Alpine marmot)	Guca1B_mma	207	5609	150	1402	121	709	128	NW_025999982.1: 9867922-9876247
		Guca1C_mma	204	17662	150	3645	88	6859	194 (-1) / 248	NW_025999968.1: 9991562-10020417
	Marmota monax	Guca1A_mmo	201	5580	150	317	94	487	164	NW_025722598.1: 360743-367735
	(Groundhog)	Guca1B_mmo	207	??	??	??	??	??	??	NW_025722598.1: 353577-353577 (Exon 1)
		Guca1C_mmo	204	21067	150	3654	88	6853	248	NW_025723719.1: 2348939-2381202
**Sciuromorpha**	*Cynomys gunnisoni*	Guca1A_cgu	201	8786	150	350	94	486	164	WBRW01011883.1: 453174-463404
	(Gunnison's prairie dog)	Guca1B_cgu	207	5445	150	1356	121	692	128	WBRW01011883.1: 441799-449897
		Guca1C_cgu	204	16053	150	3695	88	6991	155	WBRW01002940.1: 119366-146701
	Ictidomys tridecemlineatus	Guca1A_itr	201	8545 (Ns)	150	347	94	486	164	NW_024404946.1: 46259130-46269116
	(Thirteen-lined ground	Guca1B_itr	207	5464	150	1370	121	698	128	NW_024404946.1: 46272385-46280522
	squirrel)	Guca1C_itr	204	17254	150	3691	88	7009	167	NW_024405602.1: 140289570-140318132
	Urocitellus parryii	Guca1A_upa	201	8539 (Ns)	150	345	94	486	164	NW_020539913.1: 8575462-8585440
	(Arctic ground squirrel)	Guca1B_upa	207	5475	150	1375	121	697	128	NW_020539913.1: 8589580-8597732
		Guca1C_upa	204	17374	150	3704	88	7185	167	NW_020539893.1: 2545161-2574032
	Xerus inauris	Guca1A_xin	201	5965	150	593	94	535	161	PVHX01008096.1: 75639-83338
	**(Cape ground squirrel)**	Guca1B_xin	207	4042	150	1481	121	680	128	PVHX01008096.1: 64736-71544
		Guca1C_xin	204	17914	150	3604	88	6400	155	PVHX01000688.1: 226290-254804
	Glaucomys volans	Guca1A_gvo	201	10960	150	346	94	450	161	JAJEJO010001729.1: 5262-17623
	(Southern flying squirrel)	Guca1B_gvo	207	??	150	??	121	823	128	JAJEJO010002722.1: 810-1017 (Exon 1)
		Guca1C_gvo	204	17920	150	3975	88	6646	155	JAJEJO010000993.1: 85761-114898
	Sciurus vulgaris	Guca1A_svu	201	3249	150	322	94	460	164	LR738617.1: 115530833-115535472
	(Red squirrel)	Guca1B_svu	207	5637	150	1426	121	721	128	LR738617.1: 115518507-115526896
		Guca1C_svu	204	17478	150	3602	88	11411	155	LR738620.1: 59631718-59664805
	Oryctolagus cuniculus	Guca1A_ocu	201	4951	150	614	94	259	164	NC_067385.1: 124035750-124042179
	**(European rabbit)**	Guca1B_ocu	207	4914	150	2825	121	??	??	NC_067385.1: 124022775-124030990
		Guca1C_ocu	204 (+1 / Stops)	23419 (Ns)	153 (+1 / Stops)	12732	88	7575	155	NC_067387.1: 111641894-111686207
**orpha**	***Lepus americanus***	Guca1A_lam	201	??	150	??	94	??	164	PVJM010408828.1 (Exon 1) / PVJM010192966.1 (Exon 2)
	**(Snowshoe hare)**	Guca1B_lam	207	5303	150	2832	121	592	128	PVJM010071266.1: 386-9718
		Guca1C_lam	203 (+1 / Stop)	??	151 (+2 / Stops)	??	88 (no splice acceptor)	7754	155	PVJM010031176.1 (Exon 1) / PVJM010153432.1 (Exon 2)

		Species	Gene name	Exon1 from ATG	Intron 1	Exon 2	Intron 2	Exon 3	Intron 3	Exon 4 (till stop)	Accession Number (Gene sequence including introns)
	Lagon	*Sylvilagus bachmani* (Brush rabbit)	Guca1A_sba	201	5315	150	613	94	286	164	CM027390.1: 110468225-110475047
			Guca1B_sba	207	4603	150	2832	121	595	128	CM027390.1: 110455594-110464229
			Guca1C_sba	No open reading frame	-	-	-	-	-	-	
		Ochotona princeps (American pika)	Guca1A_opr	201	3390	150	835	94	226	161	NC_050547.1: 39595612-39600668
			Guca1B_opr	207	4556	150	775	121	728	128	NC_050547.1: 39606537-39613198
			Guca1C_opr	No open reading frame	-	-	-	-	-	-	
Carnivora	Caniformia	*Ursus arctos* (Brown bear)	Guca1A_uar	201	4684	150	325	94	273	170	NW_025929584.1: 33721634-33727530
			Guca1B_uar	207	6807	150	1589	118	576	128	NW_025929584.1: 33709509-33719083
			Guca1C_uar	204	16339	150	4376	88	8169	170	NW_025929905.1: 40605925-40635420
		***Canis lupus* (Wolf)**	Guca1A_clu	201	5543	150	529	94	265	173	HG994392.1: 12027857-12034811
			Guca1B_clu	207	5874	150	1209	121	718	128	HG994392.1: 12037458-12045864
			Guca1C_clu	204	19829	150	4144	88	9104	170	HG994417.1: 15363129-15396817
		Vulpes vulpes (Red fox)	Guca1A_vvu	201	5370	150	622	94	264	173	NW_020356442.1: 8493719-8500592
			Guca1B_vvu	207	7421	150	1206	121	730	128	NW_020356442.1: 8503236-8513198
			Guca1C_vvu	204	20232 (Ns)	150	4148	88	9238	170	NW_020356441.1: 14337114-14371343
		Ailurus fulgens (Red panda)	Guca1A_afu	201	4777	150	324	94	276	173	LNAC01000286.1: 1132467-1138461
			Guca1B_afu	207	6247	150	1599	121	675	128	LNAC01000286.1: 1141156-1150282
			Guca1C_afu	204	16319	150	4423	88	9131	170	LNAC01000146.1: 1503740-1534224
		Spilogale interrupta (Spotted skunk)	Guca1A_sin	201	4687	150	336	94	247	170	JAKZGT010000236.1: 7709567-7715451
			Guca1B_sin	207	6443	150	1607	121	679	128	JAKZGT010000236.1: 7697493-7706827
			Guca1C_sin	204 (+1)	17759	150	3196	88	8795	170 (div. stops)	JAKZGT010000399.1: 6041246-6071605
		***Mellivora capensis* (Honey badger)**	Guca1A_mca	201	5000	150	362	94	264	161	PISX010001747.1: 148619-154850
			Guca1B_mca	207	6736	150	1578	121	673	128	PISX010001747.1: 157532-167124
			Guca1C_mca	207 (-2)	14846	150 (1 stop)	4590	88	6378	119 ?	PISX010010922.1: 6117-32496
		Meles meles (European badger)	Guca1A_mme	201	4931	150	562	94	264	170	NC_060070.1: 90662066-90668437
			Guca1B_mme	207	6240	150	1538	121	681	128	NC_060070.1: 90671101-90680165
			Guca1C_mme	204	15605	150	4433	88	8796	170	NC_060069.1: 106562392-106591837
		Gulo gulo (Wolverine)	Guca1A_ggu	201	4812 (Ns)	150	??	??	??	170	CYRY02002592.1: 64713-70772
			Guca1B_ggu	207	6353	150	1596 (Ns)	121 ??	679	128	CYRY02002592.1: 73435-82668
			Guca1C_ggu	204	14460 (Ns)	150	4343 (Ns)	88	7375	170	CYRY02015714.1: 57747-84536
		***Lutra lutra* (Eurasian otter)**	Guca1A_llu	201	5004	150	337	94	264	170	NC_062283.1: 63486324-63492543
			Guca1B_llu	207	6513	150	1542	121		128	NC_062283.1: 63474346-63483668
			Guca1C_llu	204	15313	150	4623	88	8538	170	NC_062278.1: 57131517-57160602
		Mustela nigripes (Black-footed ferret)	Guca1A_mni	201	4782	150	350	94	285	170	CM039290.1: 61412581-61418612
			Guca1B_mni	207	6759	150	1543	121	677	128	CM039290.1: 61400296-61409880
			Guca1C_mni	204	14824	150	5186	88	8857	170	CM039287.1: 163463946-163434468
		**Odobenus rosmarus divergens (Walrus)**	Guca1A_oro	201	4802	150	326	94	310	170	NW_004450620.1: 675096-681148
			Guca1B_oro	207	5870	150	1554	121	709	128	NW_004450620.1: 683627-692365
			Guca1C_oro	204	20570 (Ns)	150 (-1)	4215	88	8687	170	NW_004450265.1: 3273783-3307867
		***Callorhinus ursinus* (Northern fur seal)**	Guca1A_cur	201	4862	150	342	94	302	170	NW_020312965.1: 27392735-27398855
			Guca1B_cur	207	5910	150	1557	121	713	128	NW_020312965.1: 27381489-27390274
			Guca1C_cur	204	20779	150 (-1)	4210	88	8650	170	NW_020312785.1: 46535603-46569854
		***Phoca vitulina* (Harbor seal)**	Guca1A_pvi	201	4806	150	329	94	302	173	NW_022589714.1: 40753236-40759290
			Guca1B_pvi	207	5859	150	1580	121	705	128	NW_022589714.1: 40741795-40750544
			Guca1C_pvi	204	18909	150	4198	88	8193	170	NW_022589711.1: 32578054-32609965
		***Neomonachus schauinslandi* (Hawaiian monk seal)**	Guca1A_nsc	201	4812	150	333	94	286	173	NC_058410.1: 107877868-107883916
			Guca1B_nsc	207	5921	150	1545	121	700	128	NC_058410.1: 107866629-107875400
			Guca1C_nsc	204	19341	150 (-1)	4401	88	8201	170	NC_058403.1: 54115402-54147959
		***Potos flavus* (Kinkajou)**	Guca1A_pfl	201	5246	150	339	94	314 (Ns)	170	JAABKN010000183.1: 1015954-1022467
			Guca1B_pfl	204 (+2/-1)	6372	159	1581	121 (+1)	688	125 (+1)	JAABKN010000183.1: 1003922-1013168
			Guca1C_pfl	204	15779 (Ns)	150 (+1 / 1 stop)	4379	88	8266	170 (1 stop)	JAABKN010000742.1: 756163-785197
		***Procyon lotor* (Raccoon)**	Guca1A_plo	201	5239	150	336	94	265	170	JAABKC010001054.1: 1009499-1015953
			Guca1B_plo	204 (+1)	??	??	??	??	??	??	JAABKC010001054.1: 1000001-1000203 (Exon 1)
			Guca1C_plo	204	14668	150 (1 stop)	4332 (Ns)	88	8337	170	JAABKC010000202.1: 351425-379373
	ia	*Panthera tigris* (Tiger)	Guca1A_pti	201	5283	150	364	94	277	170	NC_056664.1: 40174279-40180817
			Guca1B_pti	207	6883	150	1534	121		128	NC_056664.1: 40183597-40193366
			Guca1C_pti	204	18791	150	4191	88	7628	170	NC_056668.1: 55282952-55314173
		***Felis catus* (Cat)**	Guca1A_fca	201	5224	150	366	94	277	170	NC_058372.1: 40242551-40249032
			Guca1B_fca	207	6720	150	1766	121	746	128	NC_058372.1: 40251568-40261405
			Guca1C_fca	204	19244	150	4406	88	7874	170	NC_058376.1: 55744082-55776217
		Puma concolor (Cougar)	Guca1A_pco	201	??	??	??	??	??	??	NW_020339445.1: 110706480-110706680 (Exon 1)
			Guca1B_pco	207	7204	150	??	121	??	??	NW_020339445.1: 110687341-110696746 (Exons 1-3)
			Guca1C_pco	204	19126	150	4175	88	7612	170	NW_020338336.1: 56691712-56723236
		Cryptoprocta ferox (Fossa)	Guca1A_cfe	201	5254	150	362	94	182 (Ns)	176	PJEU01000406.1: 149037-155455
			Guca1B_cfe	207	7062	150	1488	121	685	128	PJEU01000406.1: 157976-167816
			Guca1C_cfe	207	18932	150	4167	88	7427	167	PJEU01002126.1: 213335-244472

Order	Suborder	Species	Gene name	Exon1 from ATG	Intron 1	Exon 2	Intron 2	Exon 3	Intron 3	Exon 4 (till stop)	Accession Number (Gene sequence including introns)
	Feliform	***Mungos mungo***	Guca1A_mmun	201	5276	150	355	94	266	176	PISW01000220.1: 52461-58978
		**(Banded mongoose)**	Guca1B_mmun	207	6415	150	1431	121	637	128	PISW01000220.1: 61495-70583
			Guca1C_mmun	204	17273	150	4380	88	7511	170	PISW01010445.1: 1346-31121
		Proteles cristata	Guca1A_pcri	201	5466	150	377	94	274	176	JAESUZ010002235.1: 419837-426574
		(Aardwolf)	Guca1B_pcri	207	8674	150	1629	121	688	128	JAESUZ010002235.1: 405490-417086
			Guca1C_pcri	204 (+1)	21601	150 (-1 / 1 stop)	4440	88 (+2)	9020	137 ? (-1)	JAESUZ010004548.1: 501463-537101
		Hyaena hyaena	Guca1A_hhy	201	5506	150	363	94	276	176	NW_024080847.1: 687038-693803
		**(Striped hyena)**	Guca1B_hhy	207	8144	150	1666	121	688	128	NW_024080847.1: 673174-684277
			Guca1C_hhy	204 (+1)	19557	150 (+1 / 1 stop)	4150	88 (+2)	8714	185 (-3)	NW_024080883.1: 528608-561654
		Crocuta crocuta	Guca1A_ccr	201	5259	150	364	94	276	176	VOAJ01003278.1: 6437813-6444332
		(Spotted hyena)	Guca1B_ccr	207	7513	150	1738	121	685	128	VOAJ01003278.1: 6424906-6435447
			Guca1C_ccr	204 (+1)	20069 (Ns)	153 (1 stop)	4145	88 (+2)	12699 (Ns)	?? (many stops)	VOAJ01004433.1: 6404628-6437889
		Paradoxurus hermaphroditus	Guca1A_phe	201	5255	150	399	94	290	170	PITB01010312.1: 4628-11186
		(Asian palm civet)	Guca1B_phe	207	??	150	864	121	659	128	PITB01014114.1: 3125-3332 (Exon 1)
			Guca1C_phe	207 (-1)	14629	150	4136	88	7602	119 ?	PITB01016917.1: 10620-37551
Pholidota		*Manis pentadactyla*	Guca1A_mpe	201	3485	150	256	94	324	173	NW_023454901.1:4220912-4225594
		(Chinese pangolin)	Guca1B_mpe	207	4776	150	1365	121	603	128	NW_023454901.1:4228085-4235434
			Guca1C_mpe	no open reading frame...							
		Phataginus tricuspis	Guca1A_ptr	201	4545	150	249	94	323	173	SOZM010006791.1:35400-41134
		(Tree pangolin)	Guca1B_ptr	207	4766	150	1396	121	607	128	SOZM010006791.1:25570-32944
			Guca1C_ptr	no open reading frame...							
Perissodactyla		***Equus caballus***	Guca1A_eca	201	4596	150	390	94	299	191	NC_009163.3:42298716-42304636
		**(Horse)**	Guca1B_eca	207	4976	150	1345	121	673	128	NC_009163.3:42307965-42315564
			Guca1C_eca	204	16776	150	4438	88	7078	185	NC_009162.3:50796058-50824976
		Ceratotherium simum	Guca1A_csi	201	5026 (Ns)	150	366	94	304	191	NW_004454168.1:23495621-23501952
		(White rhinoceros)	Guca1B_csi	207	5637 (Ns)	150	1660	121	658	128	NW_004454168.1:23483385-23491946
			Guca1C_csi	204	19598 (Ns)	150	4300	88	7388	170	NW_004454216.1:11481956-11513853
		Tapirus terrestris	Guca1A_tte	201	4823	150	394	94	277	191	PVID01007817.1:66902-73031
		(South American tapir)	Guca1B_tte	207	5245	150	1277	121	659	128	PVID01007817.1:76377-84163
			Guca1C_tte	204	18084	150	4534	88	6928	170	PVID01000857.1:209969-240126
Artiodactyla	Suina	***Sus scrofa***	Guca1A_ssc	201	5718	150	353	94	276	173	NC_010449.5: 37289156-37296120
		**(Wild boar)**	Guca1B_ssc	216 ?	8396	150	837	121	1302	128	NC_010449.5: 37299020-37310170
			Guca1C_ssc	no open reading frame							
	Tylopoda	*Camelus dromedarius*	Guca1A_cdr	201	3469	150	357	94	293	173	NC_044530.1: 12711179-12715915
		(Dromedary)	Guca1B_cdr	204	5669	150	1409	121	825	128	NC_044530.1: 12699922-12708430
			Guca1C_cdr	204 (+1 / 2 stops)	...	no open reading frame					NC_044511.1: 75786992-75806429 (Exons 1&2)
		Vicugna pacos	Guca1A_vpa	201	3470	150	361	94	293	173	NW_021964198.1: 7155560-7160301
		(Alpaca)	Guca1B_vpa	204	5868	150	1414	121	811	128	NW_021964198.1: 7163064-7171759
			Guca1C_vpa	204 (+1/-1 / 2 stops)	19591	153 (no splice consens. / 2 stops)	....	no open reading frame			NW_021972009.1: 2357379-2377326 (Exons 1&2)
	Ruminantia	*Tragulus javanicus*	Guca1A_tja	201	5300	150	360	94	313	173	PVHZ021082433.1: 9811064-9817654
		(Java mouse-deer)	Guca1B_tja	207	8014	150	1318	121	836	128	PVHZ021082433.1: 9820719-9831492
			Guca1C_tja	204 (+1 / 3 stop / No ATG)	16232	156 (+5 / 1 stop)	...	no open reading frame			PVHZ021082394.1: 50647435-50664020 (Exons 1&2)
		Antilocapra americana	Guca1A_aam	201	5386	150	362	94	264	173	ML661332.1: 9060927-9067556
		(Pronghorn)	Guca1B_aam	207	6807	150	1255	121	870	128	ML661332.1: 9048716-9058253
			Guca1C_aam	198 (+1/-1 / 2 stops)	21727	150 (+2 / 1 stop)	...	no open reading frame			ML664163.1: 458747-480821 (Exons 1&2)
		Bos taurus	Guca1A_bta	201	5358	150	361	94	315	173	NC_037350.1: 15892199-15898850
		**(Cattle)**	Guca1B_bta	216	7457	150	1355	121	866	128	NC_037350.1: 15901482-15911774
			Guca1C_bta	198	...	no open reading frame					NC_037328.1: 53691631-53715740 (Exons 1&2)
		Tragelaphus imberbis	Guca1A_tim	201	5621	150	358	94	3400	182	SJYM01002188.1: 1071897-1081902
		(Lesser kudu)	Guca1B_tim	216	9750	150	1348	121	910	128	SJYM01002188.1: 1053933-1066555
			Guca1C_tim	198 (+1)	32095 (Ns)	147 (-1/+1 / 1 stop	...	no open reading frame			SJYM01000077.1: 2475194-2507632 (Exons 1&2)
		Ovis aries	Guca1A_oar	201	5657	150	349	94	315	173	NC_056073.1: 15927783-15934721
		(Sheep)	Guca1B_oar	207	6769	150	1584	121	920	128	NC_056073.1: 15937392-15947270
			Guca1C_oar	198 (+1/-1)	23761	148 (-1/+2 / 2 stops)	...	no open reading frame			NC_056054.1: 174713584-174737689 (Exons 1&2)
		Capra sibirica	Guca1A_csi	201	5634	150	350	94	325	173	NIYN02029889.1: 15987267-15994193
		(Siberian ibex)	Guca1B_csi	207	6781	150	1612	121	920	128	NIYN02029889.1: 15996853-16006771
			Guca1C_csi	198 (+1/-1)	23834	148 (-1/+2 / 2 stops)	...	no open reading frame			NIYN02070920.1: 12581710-12605888 (Exons 1&2)
		Ovibos moschatus	Guca1A_omo	201	5666	150	350	94	315	173	JAJUWZ010000067.1: 8798200-8805148
		(Muskox)	Guca1B_omo	207	6873	150	1609	121	919	128	JAJUWZ010000067.1: 8785538-8795544
			Guca1C_omo	198 (+1/-1)	23811	151 (+3 / 2 stops)	...	no open reading frame			JAJUWZ010000051.1: 31383084-31407240 (Exons 1&2)
		Nanger granti	Guca1A_ngr	201	5234	150	360	94		164	SJYD01050917.1: 512641-519174
		(Grant's gazelle)	Guca1B_ngr	207	6986	150	1639	121	916	128	SJYD01050917.1: 499858-510004
			Guca1C_ngr	198 (+1/-1)	23811	148 (+1/-2 / 3 stops / no splice cons.)	...	no open reading frame			SJYD01047891.1: 79901-107241 (Exons 1&2)
		Orcinus orca	Guca1A_oor	201	4725	150	360	94	316	173	NC_064568.1: 73236928-73242946
		**(Orca)**	Guca1B_oor	207	6594	150	1393	121	705	128	NC_064568.1: 73246302-73255599
			Guca1C_oor	207 (+3 / 2 stops)	18945	135 (+2)	4668	88	6445	326 ?	NC_064563.1: 93524499-93555307
		Tursiops truncatus	Guca1A_ttr	201	4750	150	407	94	316	173	NC_047043.1: 32395671-32401761
		**(Common bottlenose dolphin)**	Guca1B_ttr	207	6583	150	1388	121	707	128	NC_047043.1: 32383032-32392315
			Guca1C_ttr	204 (1 stop)	20621	147 (+1)	4680	88 (bad splice cons.)	4680	326 (stops)	NC_047037.1: 88446520-88479033

		Species	Gene name	Exon1 from ATG	Intron 1	Exon 2	Intron 2	Exon 3	Intron 3	Exon 4 (till stop)	Accession Number (Gene sequence including introns)
	Cetacea	***Monodon monoceros* (Narwhal)**	Guca1A_mmo	201	4731	150	370	94	316	173	NW_021703780.1: 32983455-32989489
			Guca1B_mmo	207	6586	150	1387	121	707	128	NW_021703780.1: 32970830-32980115
			Guca1C_mmo	207 (+2)	20373	135 (+2)	4673	88	6451	317 (1 stop)	NW_021703767.1: 55683701-55715940
		Kogia breviceps (Pygmy sperm whale)	Guca1A_kbr	201	??	150	366	94	315	173	RJWL010044097.1: 12207-12408 (Exon1)
			Guca1B_kbr	201	6194	150	1370	121	708	128	RJWL010013753.1: 36969-43519 (Exon 1&2)
			Guca1C_kbr	207 (+3 / 1 stop)	…	no open reading frame					RJWL010011254.1: 38075-38278 (Exon1)
		***Physeter catodon* (Sperm whale)**	Guca1A_pcat	201	4742	150	361	94	315	173	NC_041231.1: 76073143-76079178
			Guca1B_pcat	207	6001	150	1376	121	708	128	NC_041231.1: 76083779-76092469
			Guca1C_pcat	no open reading frame							
		***Mesoplodon bidens* (Sowerby's beaked whale)**	Guca1A_mbi	201	4295	150	364	94	323	173	PVJJ010002278.1: 9774-15373
			Guca1B_mbi	207	6547	150	1390	121	705	128	PVJJ010002278.1: 18650-27897
			Guca1C_mbi	201 (+1)	20071	129 ?	4762	82 (-1 / stops)	no further orf		PVJJ010007457.1: 13039-38283 (Exons 1-3)
		Inia geoffrensis (Amazon river dolphin)	Guca1A_ige	201	??	150	364	94	317	173	RJWO010000070.1: 1048-1249 (Exon1)
			Guca1B_ige	207	6580	150	1382	121	708	128	RJWO010003078.1: 8209-17484
			Guca1C_ige	207 (+3)	20725	144	4643	88	7141	317 (1 stop)	RJWO010010713.1: 4474-37735
		Balaenoptera musculus (Blue whale)	Guca1A_bmu	201	4758	150	361	94	316	173	NC_045795.1: 33058821-33064873
			Guca1B_bmu	207	5992	150	1381	121	708	128	NC_045795.1: 33046787-33055473
			Guca1C_bmu	204	19554	144	4471	88 (-1)	6645	317 ??	NC_045788.1: 89028371-89059797
		Eubalaena japonica (North Pacific right whale)	Guca1A_eja	201	4740	150	361	94	316	173	RJWP010052151.1: 1933-7967
			Guca1B_eja	204	5989	150	1386	121	708	128	RJWP010015874.1: 37593-43919 (Exon 1-2)
			Guca1C_eja	204	??	150 (+1)	4685	88	6653	317	RJWP010015799.1: 28121-28325 (Exon 1)
		***Eschrichtius robustus* (Gray whale)**	Guca1A_ero	201	4764	150	364	94	316	173	NIPP01000056.1: 880936-886997
			Guca1B_ero	207	6091 (Ns)	150	1387	121	709	128	NIPP01000056.1: 890456-899249
			Guca1C_ero	204	38356 (Ns)	132	4481	88 (-1)	6658	164	NIPP01003746.1: 20507-70590
		***Hippopotamus amphibius* (Hippopotamus)**	Guca1A_ham	201	4704	150	368	94	304	173	CM041176.1: 79193591-79199584
			Guca1B_ham	207	7899	150	1411	121	722	128	CM041176.1: 79202489-79213126
			Guca1C_ham	204 (+1)	19918	148 (-1)	4627	89 (+2)	8599	145	CM041177.1: 50298768-50332497
Chioptera	Microchiroptera	*Rhinolophus sinicus* (Chinese rufous horseshoe bat)	Guca1A_rsi	201	??	150	372	94	??	164	NW_017739058.1: 511613-518234 (Exons 1&4)
			Guca1B_rsi	207	6849	150 (minor class splice)	1181	121	715	128	NW_017739058.1: 521216-530566
			Guca1C_rsi	204	14750	150	3766	88	6227	155	NW_017739110.1: 5445089-5470428
		***Hipposideros armiger* (Great roundleaf bat)**	Guca1A_har	201	4537	150	363	94	241	164	NW_017731422.1: 4025828-4031577
			Guca1B_har	207	7123	150	1393	121	714	128	NW_017731422.1: 4011966-4021801
			Guca1C_har	204	??	??	??	88	10121	155	NW_017731504.1: 2227671-2265051
		Craseonycteris thonglongyai (Kitti's hog-nosed bat)	Guca1A_cth	201	5076	150	232	94	188	164	PVKE010021301.1: 9237-15341
			Guca1B_cth	partial	??	??	??	??	??	??	
			Guca1C_cth	204 (-1)	??	156	??	??	??	155	PVKE010015790.1 (Exon1) / PVKE010050306.1 (Exon2)
		***Megaderma lyra* (Greater false vampire bat)**	Guca1A_mly	201	4497	150	347	94	231	164	PVJL010009441.1: 51458-57141
			Guca1B_mly	207	7909	150	1361	121	680	128	PVJL010000346.1: 3656-14211
			Guca1C_mly	204	23861	150	2509	88		155	PVJL010006601.1: 53663-80475 (Exons 1-3)
		Phyllostomus discolor (Pale spear-nosed bat)	Guca1A_pdi	201	4773	150	413	94	251	164	NC_040906.2: 115156889-115162934
			Guca1B_pdi	207	6543	150	1370	121	606	128	NC_040906.2: 115165925-115175049
			Guca1C_pdi	204	20594	150	6140	88	9756	155	NC_040904.2: 88919122-88956208
		Desmodus rotundus (Common vampire bat)	Guca1A_dro	201	4493	150	360	94	181	164	NW_020091182.1: 11706134-11711776
			Guca1B_dro	207	6438	150	1043	121	616	128	NW_020091182.1: 11694986-11703688
			Guca1C_dro	204 (+1)	18936	150	6134	88 (+1)	8218	155	NW_020093695.1: 6391515-6425397
		***Mormoops blainvillei* (Antillean ghost-faced bat)**	Guca1A_mbl	201	4564	150	414	94	244	164	PVJD01013774.1: 20461-26291
			Guca1B_mbl	**207 (2 stops)**		**144 ? (stops)**	1613	112	622	128	PVJD01082106.1 (Exon1) / PVJD01024971.1 (Exons 2-4)
			Guca1C_mbl	**204 (-1 / no splice cons.)**	??	**150 (+1 / -1)**	7092	**88 (1 stop)**	??	**221 ??**	PVJD01008113.1 (Exon1) / PVJD01016503.1 (Exon 2&3)
		Pteronotus parnellii (Parnell's mustached bat)	Guca1A_ppa	201	4548	150	394	94	249	164	JAIWKQ010000155.1:11860001-11875000
			Guca1B_ppa	**207 (1 stop)**	6531	147	1419	97	625	128	JAIWKQ010000155.1:11875001-11890000
			Guca1C_ppa	204	18870	150	4581	88	12464	155	JAIWKQ010000244.1:5790001-5840000
		***Noctilio leporinus* (Greater bulldog bat)**	Guca1A_nle	201	5413	150	426	94	248	164	PVIW01007599.1: 10796-17491
			Guca1B_nle	**204 (-1)**	??	150	1567	**121 (1 stop)**	615	128	PVIW01004136.1: 137832-138037 (Exon1)
			Guca1C_nle	204	??	150	4679	88	??	155	PVIW01005801.1: 10114-10318 (Exon1)
		Miniopterus natalensis (Natal long-fingered bat)	Guca1A_mna	201	4669	150	824 (Ns)	94	237	158	NW_015504526.1: 6664227-6670559
			Guca1B_mna	no open reading frame							
			Guca1C_mna	204	15165 (Ns)	150	1229	88	4830	155	NW_015504522.1: 46450-68270
		Pipistrellus pipistrellus (Common pipistrelle)	Guca1A_ppi	201	4562	150	296	94	245	164	LR862362.1: 9746618-9752329
			Guca1B_ppi	207	5513	150	2494	121	854	121	LR862362.1: 9755865-9765331
			Guca1C_ppi	204	20379	**165 ? (+2 / 2 stops /-1 / no spice cons.)**	4512	88	10034	**200 ? (+1 / -1)**	LR862357.1: 64301996-64337574
		***Murina aurata* (Little tube-nosed bat)**	Guca1A_mau	201	4005	150	290	94	243	164	PVJC01000325.1: 28467-33613
			Guca1B_mau	207	5763	150	677	121	564	128	PVJC01000325.1: 37022-44631
			Guca1C_mau	**192 ? (+2)**	18844	**150 (+1 / 1stop / -1)**	2503	**88 (+2)**	10326	155	PVJC01006111.1: 8470-40723
		Eptesicus fuscus (Big brown bat)	Guca1A_efu	201	4115	150	297	94	243	164	NW_007370859.1: 671214-676477
			Guca1B_efu	207	5892 (Ns)	150	2156	121	576	128	NW_007370859.1: 658380-667609
			Guca1C_efu	204	20853 (Ns)	**150 (no splice cons.)**	6242	**88 (+1)**	12379 (Ns)	**155 (1 stop)**	NW_007370689.1: 1003824-1043893

	Species	Gene name	Exon1 from ATG	Intron 1	Exon 2	Intron 2	Exon 3	Intron 3	Exon 4 (till stop)	Accession Number (Gene sequence including introns)
	Lasiurus borealis (Eastern red bat)	Guca1A_lbo	201	3720	150	303	94	240	164	PVJN01000076.1: 29528-34399
		Guca1B_lbo	207	6135	150	2195	121	605	128	PVJN01000076.1: 38273-47813
		Guca1C_lbo	**204 (+1)**	??	150	??	??	??	**143 ? (+2 / 1stop)**	PVJN01041198.1 (Exon1) / PVJN01016033.1 (Exon 2&4)
	Myotis brandtii (Brandt's bat)	Guca1A_mbr	201	4345	150	247	94	242	164	NW_005371832.1: 680422-685865
		Guca1B_mbr	207	6850	150	911	121	619	128	NW_005371832.1: 667900-676885
		Guca1C_mbr	**204 (+1)**	22327 (Ns)	**150 (+1)**	5401	**88 (-1)**	10465 (Ns)	**194 ??**	NW_005353584.1: 1171379-1210205
	Myotis myotis (Greater mouse-eared bat)	Guca1A_mmy	201	4261	150	276	94	243	164	NW_023416379.1: 86631530-86636918
		Guca1B_mmy	207	7914	150	719	121	883	128	NW_023416379.1: 86617564-86627685
		Guca1C_mmy	204	21246	**150 (+1)**	6280	**88 (-1)**	??	??	NW_023416324.1: 146893433-146921400 (Exons1-3)
	Tadarida brasiliensis **(Mexican free-tailed bat)**	Guca1A_tbr	201	??	150	??	94	??	164	PVIG010073698.1 (Exon1) / PVIG010443861.1 (Exon2)
		Guca1B_tbr	207	5705	150	1328	121	898	128	PVIG010009719.1: 1234-9770
		Guca1C_tbr	204	??	150	??	88	??	215 ??	PVIG010015440.1 (Exon1) / PVIG010269000.1 (Exon2)
**Megachiroptera**	*Eonycteris spelaea* (Cave nectar bat)	Guca1A_esp	201	4513	150	397	94	247	161	PUFA01000069.1: 6706316-6712078
		Guca1B_esp	207	5909	150	1436	121	675	128	PUFA01000069.1: 6715443-6724068
		Guca1C_esp	204	17512	150	4024	88	7432	155	PUFA01000235.1: 1694136-1723700
	Macroglossus sobrinus (Long-tongued fruit bat)	Guca1A_mso	201	4551	150	397	94	248	161	PVKZ01000438.1: 644317-650118
		Guca1B_mso	207	5936	150	1407	121	676	128	PVKZ01000438.1: 653506-662130
		Guca1C_mso	201	17419	**153 (+2 / 1 stop) No splice consens.**	4028	88	4552	155	PVKZ01001860.1: 57854-84447
	Pteropus vampyrus **(Large flying fox)**	Guca1A_pva	201	6918 (Ns)	150	408	94	248	161	NW_011888799.1: 5382032-5394134
		Guca1B_pva	207	5893	150	1435	121	683	128	NW_011888799.1: 5397473-5406179
		Guca1C_pva	204	18902 (Ns)	150	4037	88	7037	155	NW_011888782.1: 35202311-35232883
	Rousettus aegyptiacus (Egyptian fruit bat)	Guca1A_rae	201	4568	150	399	94	244	161	NW_023416284.1: 98947745-98953561
		Guca1B_rae	207	5900	150	1502	121	657	128	NW_023416284.1: 98956937-98965601
		Guca1C_rae	204	17472	150	4029	88	6655	128	NW_023416307.1: 63893885-63922637
**Eulipotyphla**	***Sorex araneus*** **(Common shrew)**	Guca1A_sar	201	4344	150	847	94	696	164	NW_004545873.1:843265-849760
		Guca1B_sar	-	-	-	-	-	-	-	
		Guca1C_sar	no open reading frame…							
	Erinaceus europaeus **(European hedgehog)**	Guca1A_eeu	201	8182	150	299	94	221	164	NW_006804366.1:426773-436083
		Guca1B_eeu	207	7064	150	2187	118	1533 (Ns)	128	NW_006804366.1:409958-421344
		Guca1C_eeu	204	53062 (Ns)	150	1893	88	18112 (Ns)	170	NW_006805146.1:293787-367465
	Solenodon paradoxus (Hispaniolan solenodon)	Guca1A_spa	201	4891	150	250 (Ns)	94	661 (Ns)	167	RJWH01002261.1:229635-235704
		Guca1B_spa	207	4300	150	1265	121	701	128	RJWH01002261.1:219919-226790
		Guca1C_spa	no open reading frame…							
	Uropsilus gracilis **(Gracile shrew mole)**	Guca1A_ugr	201	3095	150	342	94	427	173	PVHY01000260.1:42029-46510
		Guca1B_ugr	207	4032	150	2096	121	693	128	PVHY01000260.1:49530-56956
		Guca1C_ugr	207 (+1)	17571	150	5124	91 (+1)	??	155 (+2)	PVHY01001815.1:4129-27269 (Exon1-3)
	Galemys pyrenaicus (Pyrenean desman)	Guca1A_gpy	201	5116 (Ns)	150	5611	94	259	167	JAGFMF010011803.1:8799291-8805622
		Guca1B_gpy	213 (+2)	3474	156 ?	1050	118 (1stop)	1109	155 (+3 / 1 stop)	JAGFMF010011803.1:8790844-8797113
		Guca1C_gpy	no open reading frame…							
	Scalopus aquaticus **(Eastern mole)**	Guca1A_saq	201	4399	150	394	94	300	167	PVIJ01012223.1:9480-15184
		Guca1B_saq	207	??	150	1058	121 (+1 / no splice cons.)	673	128	PVIJ01001169.1:214966-215173 (Exon1)
		Guca1C_saq	no open reading frame…							
	Condylura cristata **(Star-nosed mole)**	Guca1A_ccr	201	4776 (Ns)	150	328	94	282	161	NW_004567102.1:8973961-8979952
		Guca1B_ccr	198 (2 stops)	4880	147	1440	121 (+1 / 1 stop)	676	122 (+1)	NW_004567102.1:8981601-8989182
		Guca1C_ccr	no open reading frame…							
	Talpa occidentalis (Spanish mole)	Guca1A_toc	201	3933	150	339	94	221	173	NW_023602073.1: 63855236-63860346
		Guca1B_toc	207	4709	150	1154	121	1068	128	NW_023602073.1: 63863445-63870981
		Guca1C_toc	204 (+1)	15165	150	10423	88	4885	185 ?	NW_023604295.1: 55070025-55101123
**Xenarthra**	***Choloepus hoffmanni*** **(Hoffmann's two-toed sloth)**	Guca1A_cho	201	6627	150	7093 (Ns)	94	256	173	KN195696.1:780732-788548
		Guca1B_cho	207	4653	150	1381	121	709	128	KN195696.1:770313-777661
		Guca1C_cho	no open reading frame…							
	Myrmecophaga tridactyla **(Giant anteater)**	Guca1A_mti	201	??	150	372	94	??	173	PVIY010077990.1 (Exon1) / PVIY010084608.1 (Exon2&3)
		Guca1B_mti	??	??	150 (+1; no splice consens.)	2936	115 (+1 / -2)	548	129 (no stop / +1)	PVIY010040154.1
		Guca1C_mti	no open reading frame…							
	Dasypus novemcinctus **(Nine-banded armadillo)**	Guca1A_dno	201	6499	150	183	94	286	173	NW_004481207.1:58394-65979
		Guca1B_dno	207	3887	??	??	118	687	209 ??	NW_004481207.1:49369-55820
		Guca1C_dno	no open reading frame…							
**otheria**	***Procavia capensis*** **(Rock hyrax)**	Guca1A_pca	201	9057	150	552	94	504	164	PVIO02826110.1:33228026-33238747
		Guca1B_pca	207	5761	150	1297	112	915	128	PVIO02826110.1:33215558-33224127
		Guca1C_pca	no open reading frame…							
	Chrysochloris asiatica **(Cape golden mole)**	Guca1A_cas	201	10572	150	348	94	564	164	NW_006408556.1:13242295-13254387
		Guca1B_cas	207 (+1 / no ATG)	6574	150 (1 stop)	3175	91 (+2)	1342	??	NW_006408556.1:13257934-13269840
		Guca1C_cas	no open reading frame…							
	Elephantulus edwardii **(Cape elephant shrew)**	Guca1A_eed	201	8043	150	715	94	681	152	NW_006399767.1:15452011-15462046
		Guca1B_eed	207	4453	150	594	121	1530	128	NW_006399767.1:15438032-15445211
		Guca1C_eed	201 (+1 / 1 stop)		150 (+1 / 2 stops)	??	??	??	170 (+1)	
	Trichechus manatus latirostris **(West Indian manatee)**	Guca1A_tma	201	6159	150	341	94	535	167	NW_004444001.1:7781885-7789531
		Guca1B_tma	207	6168	150	1421	121	910	128	NW_004444001.1:7793630-7802734
		Guca1C_tma	195 (3 stops)	18277 (Ns)	154 ?? (+1)	…	…	…	…	NW_004443991.1:12508782-12527406 (Exon1&2)

	Species	Gene name	Exon1 from ATG	Intron 1	Exon 2	Intron 2	Exon 3	Intron 3	Exon 4 (till stop)	Accession Number (Gene sequence including introns)
Af	*Echinops telfairi* (Madagascar hedgehog)	Guca1A_ete	201	7627 (Ns)	150	301	94	526	147 + 8	NW_022107432.1:23969045-23978091
		Guca1B_ete	??	??	150	1264	118	763	119 (+1)	NW_022107432.1:23961471-23963883 (Exons 2-4)
		Guca1C_ete	no open reading frame…							
	Orycteropus afer (Aardvark)	Guca1A_oaf	201	10648 (Ns)	150	387	94	833	164	NW_006921621.1:16548445-16560440
		Guca1B_oaf	207	9368	150	1414	121	1190	128	NW_006921621.1:16531060-16543637
		Guca1C_oaf	no open reading frame…							
	***Loxodonta africana* (African bush elephant)**	Guca1A_laf	201	6694	150	381	94	695	164	NW_003573420.1:122345436-122353814
		Guca1B_laf	207	6033	150	1386	121	923	128	NW_003573420.1:122332689-122341636
		Guca1C_laf	204 (1 Stop / No splice cons. / +2	19664	150 (+1)	??	??	??	??	NW_003573532.1:346366-366377
Metatheria	*Dasyurus viverrinus* (Eastern quoll)	Guca1A_dvi	201	6320	150	5428	94	474	161	CM037000.1:198939769-198952596
		Guca1B_dvi	n.d.							
		Guca1C_dvi	204	33700	150	5021	88	14145	131	CM036999.1:291457067-291510505
	***Sarcophilus harrisii* (Tasmanian devil)**	Guca1A_sha	201	5439	150	6248	94	454	161	NC_045429.1:271476478-271489224
		Guca1B_sha								
		Guca1C_sha	204	39361	150	5078	88	16872	131	NC_045428.1:288091266-288153149
	***Myrmecobius fasciatus* (Numbat)**	Guca1A_mfa	201	7085	150	5686	94	490	161	JAJPUD010006481.1:88064-101930
		Guca1B_mfa								
		Guca1C_mfa	204	49485 (Ns)	150	4979	88	14159	131	JAJPUD010000054.1:638257-707452
	***Thylacinus cynocephalus* (Thylacine)**	Guca1A_tcy	201	5448	150	5301	94	536	161	CM040577.1:278952639-278964529
		Guca1B_tcy								
		Guca1C_tcy	204	44614 (Ns)	150	5935	88	12082 (Ns)	131	CM040576.1:297074758-297137961
	***Monodelphis domestica* (Gray short-tailed opossum)**	Guca1A_mdo	201	10799 (Ns)	150	2912	94	524	161	NC_008802.1:285335224-285350064
		Guca1B_mdo								
		Guca1C_mdo	204	22766	150	6242	88	9732	131	NC_008804.1:68986403-69025715
	***Notamacropus eugenii* (Tammar wallaby)**	Guca1A_neu	201	??	??	??	94	567	161	GL126717.1 (Exon1) / GL052756.1 (Exon 3&4)
		Guca1B_neu								
		Guca1C_neu	??	??	150	??	??	??	137	GL055092.1 (Exon 2 & 4)
	***Gymnobelideus leadbeateri* (Leadbeater's possum)**	Guca1A_gle	201	10543	150	3770	94	554	161	WOXC01006421.1:1246507-1261979
		Guca1B_gle								
		Guca1C_gle	204	41244	150	8455	88	16893	131	WOXC01007783.1:135730-202895
	***Phascolarctos cinereus* (Koala)**	Guca1A_pci	201	9503	150	3757	94	547	161	NW_018344352.1:192464-206876
		Guca1B_pci								
		Guca1C_pci	204	31832	150	5333	88	14866	131	NW_018344031.1:11001684-11054287
	***Vombatus ursinus* (Common wombat)**	Guca1A_vur	201	??	150	??	94	549	161	NW_020954604.1:12758357-12758558 (Exon1)
		Guca1B_vur								
		Guca1C_vur	204	29643 (Ns)	150	5280	88	5591	134	NW_020941197.1:18241385-18282474
	***Trichosurus vulpecula* (Common brushtail possum)**	Guca1A_tvu	201	8984	150	3759	94	558	161	NC_050579.1:244437081-244450987
		Guca1B_tvu								
		Guca1C_tvu	204	32313	150	5372	88	19151	131	NC_050574.1:352141812-352199220
	***Dromiciops gliroides* (Monito del monte)**	Guca1A_dgl	201	9541	150	3206	94	575	161	NC_057864.1:256059120-256073047
		Guca1B_dgl								
		Guca1C_dgl	204	29445	150	5804	88	15298	146	NC_057863.1:316931573-316982707
	***Ornithorhynchus anatinus* (Platypus)**	Guca1A_oan	201	3296	150	2199	94	940	152	NC_041734.1:57838126-57845157
		Guca1B_oan	207	5548	150	3553	121	696	122	NC_041734.1:57852414-57862810
		Guca1C_oan	192	4896	150	2456	88	1553	149	NC_041744.1:36382939-36392422
Gekkota	***Gekko japonicus* (Japanese gecko)**	Guca1A_gja	198	16951 (Ns)	150	2134 (Ns)	91	862	158	NW_015165378.1: 23340-43883
		Guca1Ab_gja	198	??	150	??	91	2104	128	NW_015168892.1 (Exon 1) / NW_015286333.1 (Exon 2)
		Guca1B_gja	204	9291	150	10086 (Ns)	115	798	122	NW_015165378.1: 66676-87441
		Guca1Bb_gja	-	-	-	-	-	-	-	
		Guca1C_gja	189	15693 (Ns)	150	3457 (Ns)	88	965	131	NW_015176507.1: 237367-258039
	***Sphaerodactylus townsendi* (Townsend's least gecko)**	Guca1A_sto	198	10083	150	1322	91	1057	161	NC_059429.1: 91793522-91806583
		Guca1Ab_sto	195	24271	150	4656	91	??	??	NC_059430.1: 3423618-3452980 (Exons 1-3)
		Guca1B_sto	204	11533	150	9948	115	773	128	NC_059429.1: 91753190-91775940
		Guca1Bb_sto	-							
		Guca1C_sto	**216 many stops**	31639	150	??	??	??	**131 (+1)**	NC_059428.1: 45374900-45419440
	Paroedura picta (Ocelot gecko)	Guca1A_ppic	198	8724	150	1077	91	1100	161	BDOT02000004.1: 38838983-38850483
		Guca1Ab_ppic	198	3252	150	1297	91	500	128	BDOT02000006.1: 1310337-1315952
		Guca1B_ppic	204	6935 (Ns)	150	2457	115	600	122	BDOT02000004.1: 38866400-38876988
		Guca1Bb_ppic								
		Guca1C_ppic	**?? (stops)**	??	150	??	**partial (stops)**	??	134 ?	BDOT02000005.1: 35307480-35318916
erata	***Lacerta viridis* (European green lizard)**	Guca1A_lvi	198	8124	150	320	91	885	152	OFHU01002337.1: 151859-161778
		Guca1Ab_lvi	198	6891	150	933	91	4571	131	OFHU01000328.1: 1175508-1188472
		Guca1B_lvi	204	7068	150	5125	115	2608	122	OFHU01002337.1: 121401-136793
		Guca1Bb_lvi	195	179	150	2170	109	2977	170	OFHU01001368.1: 105140-111089
		Guca1C_lvi	204	45626	150	1709	88	2290	131	OFHU01004209.1: 81020-131217

		Species	Gene name	Exon1 from ATG	Intron 1	Exon 2	Intron 2	Exon 3	Intron 3	Exon 4 (till stop)	Accession Number (Gene sequence including introns)
**Lepidosauria**	**Late**	***Aspidoscelis tigris* (Western whiptail)**	Guca1A_ati	198	10697	150	1244	91	2361	161	MTQE01003428.1: 822926-837827
			Guca1Ab_ati	198	2711	150	729	91	1956	131	MTQE01001065.1: 515059-521024
			Guca1B_ati	204	14592	150	4165	115		131	MTQE01003428.1: 781811-802459
			Guca1Bb_ati	-	-	-	-	-	-	-	
			Guca1C_ati	204	44950 (Ns)	150	2605	88	2184	131	MTQE01002494.1: 13129352-13179663
	**Anguimorpha**	***Varanus komodoensis* (Komodo dragon)**	Guca1A_vko	198	7347	150	946	91	509	161	NW_025335268.1: 133684647-133694048
			Guca1Ab_vko	198	2178	150	1617	91	1892	131	NW_025335268.1: 75129639-75135895
			Guca1B_vko	204	7749	150	3105	115	1103	128	NW_025335268.1: 133657816-133670369
			Guca1Bb_vko	195	176	150	3825	109	2572	137	NW_025335761.1: 2918594-2925757
			Guca1C_vko	204	44794	150	2491	88	2955	131	NW_025335941.1: 32557006-32607818
	**Iguania**	***Anolis carolinensis* (Green anole)**	Guca1A_aca	198	10485	150	2076	91	1266	158	NC_014779.1: 126232449-126246872
			Guca1Ab_aca	195	10880	150	??	??	??	??	NW_003341062.1: 142-11366 (Exons 1&2)
			Guca1B_aca	204	16803	150	1205	115	3196	128	NC_014779.1: 126191369-126213169
			Guca1Ab_aca	195	171	150	??	??	??	??	NW_003338830.1: 935078-938819
			Guca1C_aca	204	57776 (Ns)	150	2864	88	1256	131	NC_014778.1: 168953269-169015737
		***Pogona vitticeps* (Central bearded dragon)**	Guca1A_pvi	198	8328	150	1444	91	1512	161	NW_018150723.1: 4592704-4604587
			Guca1Ab_pvi	-	-	-	-	-	-	-	
			Guca1B_pvi	204	16444 (Ns)	150	2475	115	3463 (Ns)	128	NW_018150723.1: 4623320-4646298
			Guca1Bb_pvi	195	261	150	928	109	857	137	NW_018150737.1: 1554569-1557205
			Guca1C_pvi	204	64427 (Ns)	150	2731	88	721	131	NW_018151126.1: 841840-910291
	**Serpentes**	***Python bivittatus* (Burmese python)**	Guca1A_pbi	198	5429 (Ns)	150	1774 (Ns)	91	1635 (Ns)	161	NW_006532570.1: 57788-67225
			Guca1Ab_pbi	-	-	-	-	-	-	-	
			Guca1B_pbi	204	4110	150	1103	115	1052	128	NW_006532570.1: 42105-48966
			Guca1Bb_pbi	-	-	-	-	-	-	-	
			Guca1C_pbi	192	14194 (Ns)	150	2684	88	1552	131	NW_006533900.1: 49455-68445
		***Ophiophagus hannah* (King cobra)**	Guca1A_oha	198	3024	150	125	91	1527	158	AZIM01000597.1: 105546-110818
			Guca1Ab_oha	-	-	-	-	-	-	-	
			Guca1B_oha	204	4419	150	670	115	1068	128	AZIM01000597.1: 90738-97491
			Guca1Bb_oha	-	-	-	-	-	-	-	
			Guca1C_oha	192	7452	150	948	88	??	??	AZIM01003050.1: 1255-10084 (Exons 1-3)
		Hydrophis hardwickii (Spine-bellied sea snake)	Guca1A_hha	198	??	150	119	91	1592	158	RSAD01612050.1 (Exon 1)<br>RSAD01608655.1: 1039-3153 (Exons 2-4)
			Guca1Ab_hha	-	-	-	-	-	-	-	
			Guca1B_hha	??	??	150	715	115	997	128	RSAD01171676.1: 6457-8561 (Exons 2-4)
			Guca1Bb_hha								
			Guca1C_hha	192	??	150	912	88	??	??	RSAD01302449.1 (Exon 1)<br>RSAD01109233.1: 997-2149 (Eons 2&3)
		Anilios bituberculatus	Guca1A_abi	198	??	150	??	91	??	149	JAJFZI010651642.1 (Exon 1) / JAJFZI010746851.1 (Exon 2)<br>JAJFZI010981005.1 (Exon 3) / JAJFZI011270438.1 (Exon 4)
			Guca1Ab_abi	-	-	-	-	-	-	-	
			Guca1B_abi	204	??	150	??	112	??	128	JAJFZI011268083.1 (Exon 1) / JAJFZI010713786.1 (Exon 2)<br>JAJFZI010087482.1 (Exon 3) / JAJFZI011286616.1 (Exon 4)
			Guca1Bb_abi	-	-	-	-	-	-	-	
			Guca1C_abi	n.d							
	**Sphenodontia**	***Sphenodon punctatus* (Tuatara)**	Guca1A_spu	198	15769	150	827	91	12244	164	QEPC01000842.1: 798312-827754
			Guca1Ab_spu	198	29715 (Ns)	150	1045	91	1757	128	QEPC01003334.1: 98314-131397
			Guca1B_spu	204	11868 (Ns)	150	7747 (Ns)	115	3317	131	QEPC01000842.1: 723764-747295
			Guca1Bb_spu	195	206	150	12345 (Ns)	109	4381 (Ns)	161	QEPC01001391.1: 978507-996053
			Guca1C_spu	204	75662 (Ns)	150	15510 (Ns)	88	17334 (Ns)	131	QEPC01015351.1: 1975771-2084849
**Testudines**		***Chrysemys picta* (Painted turtle)**	Guca1A_cpi	198	5957 (Ns)	150	724	91	2921 (Ns)	161	NC_024230.2: 22952300-22962501
			Guca1Ab_cpi	198	8366 (Ns)	150	655	91	4360	128	NW_024885768.1: 9061-23008
			Guca1B_cpi	204	3612	150	3215	115	1939	128	NC_024230.2: 22980915-22990277
			Guca1B_cpi	-	-	-	-	-	-	-	
			Guca1C_cpi	204	37514 (Ns)	150	3189	88	3766	131	NC_024218.2: 91006114-91051155
		Pelodiscus sinensis (Chinese softshell turtle)	Guca1A_psi	198	5148	150	682	91	558	161	NW_005853904.1: 769652-776639
			Guca1Ab_psi	198	25655	150	1703	91	4575	128	NW_005854421.1: 411503-444002
			Guca1B_psi	204	??	??	??	??	??	??	NW_005853904.1: 754414-754617 (Exon 1)
			Guca1Bb_psi	-	-	-	-	-	-	-	
			Guca1C_psi	204	38016 (Ns)	150	803	88	8127	131	NW_005853487.1: 3639503-3687021
		Chelonia mydas (Green sea turtle)	Guca1A_cmy	198	6908	150	748	91	3197	161	NC_051261.2: 3598663-3610115
			Guca1Ab_cmy	198	8220	150	5240	91	4185	128	NC_057849.1: 348238720-348256931
			Guca1B_cmy	204	3624	150	6407	115	1932	128 (+1)	NC_051261.2: 3629218-3641776
			Guca1Bb_cmy	-	-	-	-	-	-	-	
			Guca1C_cmy	204	25158	150	3247	88	5276	131	NC_057849.1: 189343259-189377512
		Chelonoidis abingdonii (Pinta Island tortoise)	Guca1A_cab	198	4865	150	765	91	2703	161	NW_022643477.1: 1544423-1553355
			Guca1Ab_cab	198	9266	153	1676	91	7252 (Ns)	128	NW_022642953.1: 5214-23977
			Guca1B_cab	204	3604	150	3205	115	1851	128	NW_022643477.1: 1521856-1531112
			Guca1Bb_cab	-	-	-	-	-	-	-	
			Guca1C_cab	204	56778 (Ns)	150	3460	88	5756	131	NW_022647995.1: 292157-358723

Group	Species	Gene name	Exon1 from ATG	Intron 1	Exon 2	Intron 2	Exon 3	Intron 3	Exon 4 (till stop)	Accession Number (Gene sequence including introns)
Crocodylia	***Alligator mississippiensis* (American alligator)**	Guca1A_ami	198	1773	150	427	91	991	158	NW_017712480.1: 65361-69148
		Guca1Ab_ami	198	5718	150	482	91	708	128	NW_017711672.1: 30843-38317
		Guca1B_ami	204	4948	150	2358	115	862	128	NW_017712480.1: 44404-53168
		Guca1Bb_ami	-	-	-	-	-	-	-	
		Guca1C_ami	201	52211 (Ns)	150	1977	88	4685	131	NW_017713645.1: 16034133-16093575
	Gavialis gangeticus (Gharial)	Guca1A_ggan	198	1744	150	398	91	949	158	NW_017729019.1: 11203978-11207665
		Guca1Ab_ggan	198	??	150	1463	91	705	128	JRWT01276011.1 (Exon 1) / JRWT01276010.1 (Exons 2-4)
		Guca1B_ggan	204	5032	150	2681	115	857	128	NW_017729019.1: 11177796-11186962
		Guca1Bb_ggan	-	-	-	-	-	-	-	
		Guca1C_ggan	201	75231 (Ns)	150	7221	88	4009	131	NW_017729032.1: 257968507-258055236
	Crocodylus porosus (Saltwater crocodile)	Guca1A_cpo	198	1791	150	430	91	989	158	NW_017728935.1: 7391487-7395293
		Guca1Ab_cpo	198	5705	150	1471	91	3780 (Ns)	128	JRXG02002270.1: 105262-116784
		Guca1B_cpo	204	4984	150	2638	115	828	128	NW_017728935.1: 7407581-7416627
		Guca1Bb_cpo	-	-	-	-	-	-	-	
		Guca1C_cpo	201	43746 (Ns)	150	1992 (Ns)	88	3900 (Ns)	131	NW_017728906.1: 3433308-3483515
[illegible]	***Gallus gallus* (Chicken)**	Guca1A_gga	198	1286	150	1869	91	319	161	NC_052557.1: 3220728-3224801
		Guca1Ab_gga	198	1240	150	1888	91	1097	128	NC_052532.1: 446276-451067
		Guca1B_gga	204	801	150	1262	115	554	128	NC_052557.1: 3213871-3217084
		Guca1Bb_gga	-	-	-	-	-	-	-	
		Guca1C_gga	195	15540	150	2838	88	2151	131	NC_052532.1: 87915134-87936226
	Cygnus buccinator (Trumpeter swan)	Guca1A_cbu	198	1793	150	574	91	371	161	JAHMHU010000030.1: 1315498-1318835
		Guca1Ab_cbu	198	2238	150	1502	91	998	128	JAHMHU010000478.1: 17250-22554
		Guca1B_cbu	204	847	150	1324	115	633	128	JAHMHU010000030.1: 1324743-1328143
		Guca1Bb_cbu	-	-	-	-	-	-	-	
		Guca1C_cbu	195	13847	150	2706	88	2986	131	JAHMHU010000012.1: 15844310-15864412
	Aquila chrysaetos (Golden eagle)	Guca1A_ach	198	1935	150	564	91	407	161	NW_021941859.1: 1449234-1452739
		Guca1Ab_ach	198	2596	150	1720	91	1051	128	NW_021941840.1: 91627-97560
		Guca1B_ach	204	2069	150	1446	115	627	128	NW_021941859.1: 1459753-1464491
		Guca1Bb_ach	-	-	-	-	-	-	-	
		Guca1C_ach	204	28088	150	2776	88	3062	131	NW_021941842.1: 11560556-11595054
	***Apteryx mantelli* (North Island brown kiwi)**	Guca1A_aman	198	1552	150	??	??	??	??	NW_013992894.1 (Exons 1&2)
		Guca1Ab_aman	198	2575	150	3830	91	1144	128	NW_014004922.1: 18608-26723
		Guca1B_aman	204	2361	150	1559	115	652	128	NW_013998964.1: 99970-105138
		Guca1Bb_aman	-	-	-	-	-	-	-	
		Guca1C_aman	207 (-1 / possible stop)	34866 (Ns)	150	2844	88 (RNA editing GT -> GC)	2874	131	NW_013998331.1: 9432020-9473180
	Apteryx rowi (Okarito kiwi)	Guca1A_aro	198	1552	150	410	91	291	161	NW_020450705.1: 1443814-1446666
		Guca1Ab_aro	198	2575	150	2243	91	1144	128	NW_020447998.1: 1125-7653
		Guca1B_aro	204	2363	150	1559	115	652	128	NW_020450705.1: 1452786-1457956
		Guca1Bb_aro	-	-	-	-	-	-	-	
		Guca1C_aro	207 (-1 / possible stop)	33235	150	2844	88 (RNA editing GT -> GC)	2874	131	NW_020447708.1: 1326800-1366329
	Apteryx owenii (Little spotted kiwi)	Guca1A_aow	198	1547	150	410	91	192	161	PTFC01000039.1: 1441578-1444426
		Guca1Ab_aow	198	2575	150	2243	91	1139	128	PTFC01000558.1: 626007-632530
		Guca1B_aow	204	2355	150	1559	115	653	128	PTFC01000039.1: 1450541-1455704
		Guca1Bb_aow	-	-	-	-	-	-	-	
		Guca1C_aow	207 (-1)	32892	150	2842	88	2876	131	PTFC01000107.1: 1717647-1756833
	Apteryx haastii (Great spotted kiwi)	Guca1A_aha	198	1547	150	410	91	292	161	PTFD01000043.1: 1439853-1442701
		Guca1Ab_aha	198	2575	150	2242	91	1140	128	PTFD01001947.1: 11074-17597
		Guca1B_aha	204	2355	150	1559	115	653	128	PTFD01000043.1: 1448817-1453980
		Guca1Bb_aha	-	-	-	-	-	-	-	
		Guca1C_aha	207 (-1) (195 alternative exon ??)	32891	150	2842	88	2876	131	PTFD01000042.1: 1712571-1751756
	Buceros rhinoceros (Rhinoceros hornbill)	Guca1A_brh	198	1867	150	556	91	402	161	NW_010412518.1: 14830-18254
		Guca1Ab_brh	198	2144	150	2576	91	1003	128	NW_010412742.1: 3063-9352
		Guca1B_brh	204	1919	150	1456 (Ns)	115	621	128	NW_010412518.1: 4129-8571
		Guca1Bb_brh	-	-	-	-	-	-	-	
		Guca1C_brh	195	14233	150	2731	88	3091	131	NW_010426174.1: 17411-38029
	Archilochus colubris (Ruby-throated hummingbird)	Guca1A_aco	198	1592	150	472	91	392	152	CM041110.1: 2133078-2136124
		Guca1Ab_aco	3? + 195	2154	150	1673	91	970	128	CM041095.1: 21899404-21904765
		Guca1B_aco	204	1431	150	1297	115	534	128	CM041110.1: 2141200-2145058
		Guca1Bb_aco	-	-	-	-	-	-	-	
		Guca1C_aco	204	27173	150	2704	88	4262	131	CM041083.1: 77271631-77306342
	Apus apus (Common swift)	Guca1A_aap	198	1740	150	492	91	851	161	NC_067304.1: 5822090-5825772
		Guca1Ab_aap								
		Guca1B_aap	204	1688	150	1361	118	525	128	NC_067304.1: 5812926-5817099
		Guca1B_aap								
		Guca1C_aap	204	26148	150	2690	88	3059	131	NC_067282.1: 76689857-76722326
	Steatornis caripensis (Oilbird)	Guca1A_sca	198	1845	150	597	91	??	161	VZSC01002192.1: 59799-62684
		Guca1Ab_sca	198	2675	150	1697	91	1029	128	VZSC01003015.1: 22624-28591
		Guca1B_sca	204	2362	150	1418	115	613	128	VZSC01002192.1: 69370-74359
		Guca1Bb_sca	-	-	-	-	-	-	-	
		Guca1C_sca	195	15864	150	2729	88	3074	131	VZSC01011153.1: 25463-47693

	Species	Gene name	Exon1 from ATG	Intron 1	Exon 2	Intron 2	Exon 3	Intron 3	Exon 4 (till stop)	Accession Number (Gene sequence including introns)
**Ave**	*Larus smithsonianus* (American herring gull)	Guca1A_lsm	198	??	??	??	91	390	161	WAAC01113996.1 (Exon 1)<br>WAAC01008689.1: 59623-60264 (Exons 3&4)
		Guca1Ab_lsm	198	3675	150	2534	91	1012	128	WAAC01014077.1: 6224-14011
		Guca1B_lsm	204	2052	150	1415	115	714	128	WAAC01008689.1: 48265-53041
		Guca1Bb_lsm	-	-	.	-	-	-	-	
		Guca1C_lsm	204	29299	150	2757	88	3035	131	WAAC01013830.1: 173642-209305
	Ciconia maguari (Maguari stork)	Guca1A_cma	198	1955	150	564	91	406	161	CM030213.1: 6596922-6600446
		Guca1Ab_cma	198	2628	150	1723	91	1017	128	CM030193.1: 220357381-220363316
		Guca1B_cma	204	2069	150	1434	115	622	128	CM030213.1: 6585419-6590140
		Guca1Bb_cma								
		Guca1C_cma	204	25639	150	2765	88	4465	131	CM030193.1: 122521359-122554800
	Cuculus canorus (Common cuckoo)	Guca1A_cca	198	6822 (Ns)	150	514	91	683	161	NW_009245167.1: 655186-663804
		Guca1Ab_cca	198	4318	150	2513	91	3057	128	NW_009245394.1: 91098-101553
		Guca1B_cca	204	2034	150	1303	115	628	128	NW_009245167.1: 644444-649005
		Guca1Bb_cca	-	-	-	-	-	-	-	
		Guca1C_cca	204	29803 (Ns)	150	2742	88	2548	131	NW_009244326.1: 322561-358226
	***Struthio camelus* (Common ostrich)**	Guca1A_scam	198	1350	150	409	91	188	161	NW_009270943.1: 1417822-1420368
		Guca1Ab_scam	198	2583	150	1992	91	1122	128	NW_009270393.1: 7086-13349
		Guca1B_scam	204	2344	150	1556	115	664	128	NW_009270943.1: 1430898-1436058
		Guca1Bb_scam	-	-	-	-	-	-	-	
		Guca1C_scam	204	31098 (Ns)	150	2845	88	2966	131	NW_009271190.1: 2993405-3030886
	***Taeniopygia guttata* (Zebra finch)**	Guca1A_tgu	198	2102	150	576	91	587	161	NC_044238.2: 689462-693326
		Guca1Ab_tgu	198	5065	150	1658	91	957	128	NC_044212.2: 40705-48951
		Guca1B_tgu	204	1258	150	776	115	510	128	NC_044238.2: 698749-701889
		Guca1Bb_tgu	-	-	-	-	-	-	-	
		Guca1C_tgu	204	28234	150	2664	88	2297	131	NC_044211.2: 2888041-2921809
	Ara macao (Scarlet macaw)	Guca1A_ama	198	1788	150	538	91	??	??	AOUJ01079679.1: 367-3131
		Guca1Ab_ama	198	??	150	??	91	1049	128	KE093628.1: 1647-9077
		Guca1B_ama	??	??	150	1357	115	623	128	AOUJ01079674.1: 1756-4128 (Exons 2-4)
		Guca1Bb_ama	-	-	-	-	-	-	-	
		Guca1C_ama	204	??	150	2363	88	3099 (Ns)	131	AOUJ01390547.1 (Exon 1)<br>KE018619.1: 9326-15158 (Exons 2-4)
	Strigops habroptila (Kākāpō)	Guca1A_shab	198	2057	150	540	91	368	161	NC_044294.2: 2126879-2130443
		Guca1Ab_shab	198	2927	150	2314	91	1051	128	NC_044279.2: 48161-55019
		Guca1B_shab	204	2224	150	1360	115	618	128	NC_044294.2: 2115557-2120355
		Guca1Bb_shab	-	-	-	-	-	-	-	
		Guca1C_shab	204	27931	150	2390	88	3112	131	NC_044278.2: 16686841-16720846
	***Bubo bubo* (Eurasian eagle-owl)**	Guca1A_bbu	198	1827	150	533	91	406	161	ML979882.1: 1281336-1284701
		Guca1Ab_bbu	198	2638	150	1637	91	1004	128	ML984448.1: 623708-629553
		Guca1B_bbu	204	2058	150	1421	115	582	128	ML979882.1: 1290908-1295565
		Guca1Bb_bbu	-	-	-	-	-	-	-	
		Guca1C_bbu	204	30080	150	3882	88	3061	131	ML986463.1: 2980315-3017910
	Tyto alba (Barn owl)	Guca1A_tal	198	1860	150	541	91	399	161	NW_024881305.1: 6475987-6479386
		Guca1Ab_tal	198	1951	150	1651	91	1019	128	NW_024881282.1: 91618066-91623253
		Guca1B_tal	204	2042	150	1359	115	599	128	NW_024881305.1: 6464528-6469124
		Guca1Bb_tal	-							
		Guca1C_tal	204	27427	150	2672	88	3036	131	NW_024881276.1: 48716790-48750497
	Aptenodytes patagonicus (King	Guca1A_apa	198	1939	150	564	91	392	161	VULM01013663.1: 1973747-1977241
		Guca1Ab_apa	198	5401	150	1707	91	1023	128	VULM01001583.1: 135937-144634
		Guca1B_apa	204	2086	150	1434	115	634	128	VULM01013663.1: 1961846-1966596
		Guca1Bb_apa	-	-	-	-	-	-	-	
		Guca1C_apa	192	25913	150	2771	88	3096	131	VULM01007878.1: 138190-170530
	Pelecanus crispus (Dalmatian pelican)	Guca1A_pcr	198	1941	150	564	91	404	161 ?	NW_009113248.1: 1-3424
		Guca1Ab_pcr	198	2620	150	1714	91	1038	128	NW_009060194.1: 4356-10294
		Guca1B_pcr	204	2066	150	1403	115	638	128	NW_009069742.1: 3800-8503
		Guca1Bb_pcr	-	-	-	-	-	-	-	
		Guca1C_pcr	??	??	150	2786	88	4564	131	NW_009101830.1: 15484-23202
	***Xenopus tropicalis* (Western clawed frog)**	Guca1A_xtr	198	2346	150	1021	91	962	173	NC_030678.2: 50596311-50601251
		Guca1Ab_xtr	198	2511	150	3324	91	2130	128	NC_030679.2: 89913804-89922335
		Guca1B_xtr	201	12875	150	1739	115	1485	128	NC_030678.2: 50605236-50620328
		Guca1Bb_xtr	195	2201	150	679	109	977	128	NC_030680.2: 15026363-15030801
		Guca1C_xtr	195	13946	150	3152	88	1403	134	NC_030678.2: 76263171-76282238
	***Bufo bufo* (Common toad)**	Guca1A_bbu	198	1615	150	109	91	617	170	NC_053391.1: 226682243-226685201
		Guca1Ab_bbu	198	5665	150	17232	91	1038	128	NC_053389.1: 604606820-604631321
		Guca1B_bbu	201	20470	150	19920	115	5314	128	NC_053391.1: 226580278-226626575
		Guca1Bb_bbu	195	939	150	3776	109	4734	128	NC_053398.1: 17445695-17455725
		Guca1C_bbu	195	236190	150	170264	88	1463	134	NC_053391.1: 302070934-302479417

		Species	Gene name	Exon1 from ATG	Intron 1	Exon 2	Intron 2	Exon 3	Intron 3	Exon 4 (till stop)	Accession Number (Gene sequence including introns)
**Amphibia**	**Anura**	***Nanorana parkeri* (High Himalaya frog)**	Guca1A_npa	198	3279	150	4393	91	2690	179	NW_017306683.1: 1023711-1034690
			Guca1Ab_npa	198	2671	150	6186	91	1766	128	NW_017307017.1: 91272-102461
			Guca1B_npa	201	28994 (Ns)	150	2844	115	701	128	NW_017306683.1: 1049408-1082540
			Guca1Bb_npa	195	445	**96 ?? (+3)**	637	109	1863	128	NW_017307566.1: 324347-327816
			Guca1C_npa	204	21416 (Ns)	150	15248	88	3216	134	NW_017306525.1: 757349-797804
		***Eleutherodactylus coqui* (Common coquí)**	Guca1A_eco	198	5917	150	117	91	992	179	WNTK01000829.1: 29846-37489
			Guca1Ab_eco	198	11964	150	6561	91	597	128	CM034087.1: 83680768-83700456
			Guca1B_eco	201	??	150	1119	115	3590	128	CM034089.1: 76119667-76119871 (Exon 1)
			Guca1Bb_eco	n.d.							
			Guca1C_eco	195	25324 (Ns)	150	29300 (Ns)	88	1009	134	CM034086.1: 161513281-161569510
		Leptodactylus fallax (Giant ditch frog)	Guca1A_lfa	198	2904	150	104	91	1806	179	CAMRHE010000098.1: 523979-529410
			Guca1Ab_lfa	198	14180	150	3384	91	3036	128	CAMRHE010000602.1: 645824-666990
			Guca1B_lfa	201	21852	150	5261	115	3081	128	CAMRHE010000098.1: 480428-511215
			Guca1Bb_lfa	195	1097	150	659	109	1845	128	CAMRHE010000068.1: 717072-721254
			Guca1C_lfa	195	136415	150	45458	88	5184	134	CAMRHE010000030.1: 6154166-6341789
	**Caudata**	*Ambystoma mexicanum* (Axolotl)	Guca1A_amex	198	35577	150	107126	91	24765	173	CM030328.1: 181667039-181498960
			Guca1Ab_amex	198	73278 (Ns)	150	193245 (Ns)	91	2115	128	CM030327.1: 599684181-599953385
			Guca1B_amex	201	74315	150	3509	115	6257	128	CM030328.1: 181704768-181789442
			Guca1Bb_amex	195	2335	150	54767	109	103571	128	CM030325.1: 546555243-546716497
			Guca1C_amex	195	280917	150	246634 (Ns)	88	393512 (Ns)	131	CM030316.1: 902519997-903441622
	**Gymnophiona**	***Rhinatrema bivittatum* (Two-lined caecilian)**	Guca1A_rbi	198	3110	150	1339	91	1194	152	NC_042626.1: 7470837-7477070
			Guca1Ab_rbi	198	4415	150	6861	91	4600	128	NC_042623.1: 37815200-37831642
			Guca1B_rbi	204	2631	150	3074	115	1398	104	NC_042626.1: 7456990-7464665
			Guca1Bb_rbi	-	-	-	-	-	-	-	
			Guca1C_rbi	-	-	-	-	-	-	-	
		Microcaecilia unicolor (Caecilian)	Guca1A_mun	-	-	-	-	-	-	-	
			Guca1Ab_mun	198	1113	150	5244	91	2722	128	NC_044040.1: 26358235-26367880
			Guca1B_mun	-	-	-	-	-	-	-	
			Guca1Bb_mun	-	-	-	-	-	-	-	
			Guca1C_mun	-	-	-	-	-	-	-	
		***Geotrypetes seraphini* (Gaboon caecilian)**	Guca1A_gse	-	-	-	-	-	-	-	
			Guca1Ab_gse	198	3091	150	733	91	4490	128	NC_047092.1: 23767080-23775960
			Guca1B_gse	-	-	-	-	-	-	-	
			Guca1Bb_gse	-	-	-	-	-	-	-	
			Guca1C_gse	-	-	-	-	-	-	-	
**Coelacanthidae**		*Latimeria chalumnae* (West Indian Ocean coelacanth)	Guca1A_lch	-	-	-	-	-	-	-	
			Guca1Ab_lch	198	4226	150	5077 (Ns)	91	1294	128	NW_005820726.1:236129-247292
			Guca1B_lch	201	1142	150	1680	115	355	128	NW_005819025.1:3715909-3719679
			Guca1Bb_lch	195	1020	**150 (1 stop)**	??	**109 (1 stop)**	2036	128	NW_005820718.1:35263-36627 (Ex 1&2)<br>NW_005832290.1:1481-3754 (Ex 3&4)
			Guca1C_lch	195	945	150	4901	88	1357	131	NW_005819183.1:1783513-1791279
**Holostei (Gars & Bowfins)**		*Lepisosteus oculatus* (Spotted gar)	Guca1A_loc	201	3288	150	473	91	1024	146	NC_023181.1:32693583-32698955
			Guca1Ab_loc	198	5215 (Ns)	150	701	91	1147	128	NC_023186.1:8183246-8190875
			Guca1B_loc	201	1551	150	1895	115	1490	128	NC_023181.1:32677746-32683275
			Guca1Bb_loc	192	476	150	188	109	966	128	NC_023205.1:11400280-11402488
			Guca1Bb2_loc	204	479	150	816	109	374	128	NC_023205.1:11404673-11406932
			Guca1C_loc	195	3526	150	1442	88	516	131	NC_023181.1:18644893-18650940
