# Supplementary material for: Evolution of visual guanylyl cyclases and their activating proteins with respect to clade and species-specific visual system adaptation
Source: Front Mol Neurosci. 2023 Mar 16;16:1131093. doi: 10.3389/fnmol.2023.1131093 (PMC10061024; doi:10.3389/fnmol.2023.1131093)
Supplement: Supplementary file 3 [file Data_Sheet_3.pdf]

	Species	SWS1 (violet/UV) / Accession # and localization	SWS2 (blue) / Accession # and localization	RH2 (green) / Accession # and localization	LWS (green/red) / Accession # and localization
**Prototeria**	*Ornithorhynchus anatinus*	n.d.	NC_041733.1:19394649-19399831	n.d.	NC_041733.1:19364382-19374356 (partial, first exon missing)
**Sauropsida**	*Sphenodon punctatus*	QEPC01003334.1:680945-688807	QEPC01006279.1:156590-160715	QEPC01007384.1:3768915-3789714	QEPC01006279.1:169016-172684
	Gekko japonicus	NW_015176509.1:37156-50044	n.d.	NW_015160843.1:791877-807508	NW_015160281.1:306447-311405
	Sphaerodactylus townsendi	NC_059430.1:5825563-5844609	n.d.	NC_059429.1:80959421-80978062	NC_059427.1:146393332-146398029
	Varanus komodoensis	NW_025335268.1:74676872-74681899	NW_025335972.1:2930883-2935166	NW_025335268.1:124165989-124183417	NW_025335972.1:2950318-2954466
	Lacerta viridis	OFHU01003627.1:97274-102476	OFHU01000881.1:199634-206721	OFHU01004393.1:249209-262121	OFHU01000881.1:230860-236859
	Aspidoscelis tigris	JALMGB010000006.1:94835075-94842114	JALMGB010000007.1:55366377-55376940	JALMGB010000002.1:118372621-118392633	JALMGB010000007.1:55398618-55403336
	Anolis carolinensis	NW_003339375.1:107040-120175 (some Ns)	NC_014777.1:88634625-88640085	NC_014779.1:122808417-122826851	NC_014777.1:88664911-88668635
	Pogona vitticeps	NW_018151419.1:325124-332953	NW_018150848.1:421521-425710	NW_018150701.1:1923434-1940510	NW_018150848.1:388730-392845
	Python bivittatus	NW_006538180.1:19527-26395	n.d.	n.d.	NW_006532045.1:596098-599414
	Ophiophagus hannah	AZIM01006625.1:10778-19324	n.d.	n.d.	AZIM01001307.1:124885-128046
	Anilios bituberculatus	n.d.	n.d.	n.d.	JAJFZI010060813.1: partial / JAJFZI011244404.1: partial
	Chrysemys picta	NW_024885828.1:1886115-1895403	NW_024911077.1:78405-82832	NW_024885743.1:344726-353614	NW_024911077.1:90950-106646
	Pelodiscus sinensis	NW_005851206.1:162425-160137 (partial)	NW_005852250.1:21237-25298 (frame shift mutations in Ex 4)	NW_005854035.1:1065562-1074940	NW_005852250.1:567-3094 (Ex 2-5) / NW_005855075.1:4020-4136 (Ex 6)
	Chelonoidis abingdonii	NW_022645521.1:295060-303226 (multiple sbp del. In Ex4)	NW_022642750.1:5860-6548 (Ex 1&2)	NW_022641250.1:1169461-1177966	NW_022641603.1:29739-39662
	Chelonia mydas	NC_057849.1:343469409-343476781	n.d.	NC_051261.2:10046876-10055590	XM_007052772.1
	Alligator mississippiensis	n.d.	NW_017710695.1:25472-29600	pseudogen	NW_017710695.1:33909-37764
	Gallus gallus	JAJMOL010000001.1:1032475-1040933 (partial)	NW_024096018.1:3382-17279 (partial)	NC_052557.1:4599085-4603315	NW_024096021.1:21230-33663 (partial)
	Taeniopygia guttata	NC_044212.2:173859-177323	NC_054763.1:223599-229161	NC_044238.2:1934882-1938052	NC_054763.1:233082-239291
	Struthio camelus	AY227189.3 (partial)	NW_009271387.1:2265-2628 (partial)	NW_009270925.1:403158-407538	??? (microchromosome seq. problem ??)
	Bubo bubo	n.d.	??? (microchromosome seq. problem ??)	ML981161.1:955709-960380	??? (microchromosome seq. problem ??)
	Apteryx mantelli	NW_013987559.1:60455-60694 (exon4)	NW_014006645.1:36617-36833 (exon4)	NW_014005142.1:142531-147952 (1bp insert pos 14865)	??? (microchromosome seq. problem ??)
**Amphibia**	*Xenopus tropicalis*	NC_030679.2:89117698-89124112	NC_030684.2:32333490-32338638	n.d.	NC_030684.2:32348843-32352937
	Bufo bufo	NC_053389.1:601291286-601302768	NC_053396.1:26290421-26298192	n.d.	NC_053396.1:26325770-26333531
	Nanorana parkeri	NW_017306744.1:666538-678350	NW_017306743.1:748515-762353	n.d.	NW_017306743.1:783909-800415
	Eleutherodactylus coqui	CM034087.1:85388376-85419070	CM034094.1:11307264-11322388	n.d.	CM034094.1:11341102-11346306
	Rhinatrema bivittatum	n.d.	n.d.	n.d.	NC_042615.1:500854895-500868182
	Geotrypetes seraphini	n.d.	n.d.	n.d.	NC_047084.1:457714833-457735199
**Coelacanth**	*Latimeria chalumnae*	n.d.	NW_005819712.1:539942-551698	NW_005819268.1:1631870-1638555	n.d
**Holostei**	*Lepisosteus oculatus*	NC_023186.1:10398714-10404663	NC_023179.1:2621050-2624669	NC_023181.1:36554395-36556894	NC_023179.1:2605997-2610690

Green: Sense orientation / Brown: Antisense orientation / Red: Likely pseudogenized
