# Supplementary material for: Evolution of visual guanylyl cyclases and their activating proteins with respect to clade and species-specific visual system adaptation
Source: Front Mol Neurosci. 2023 Mar 16;16:1131093. doi: 10.3389/fnmol.2023.1131093 (PMC10061024; doi:10.3389/fnmol.2023.1131093)
Supplement: Supplementary file 4 [file Data_Sheet_4.pdf]

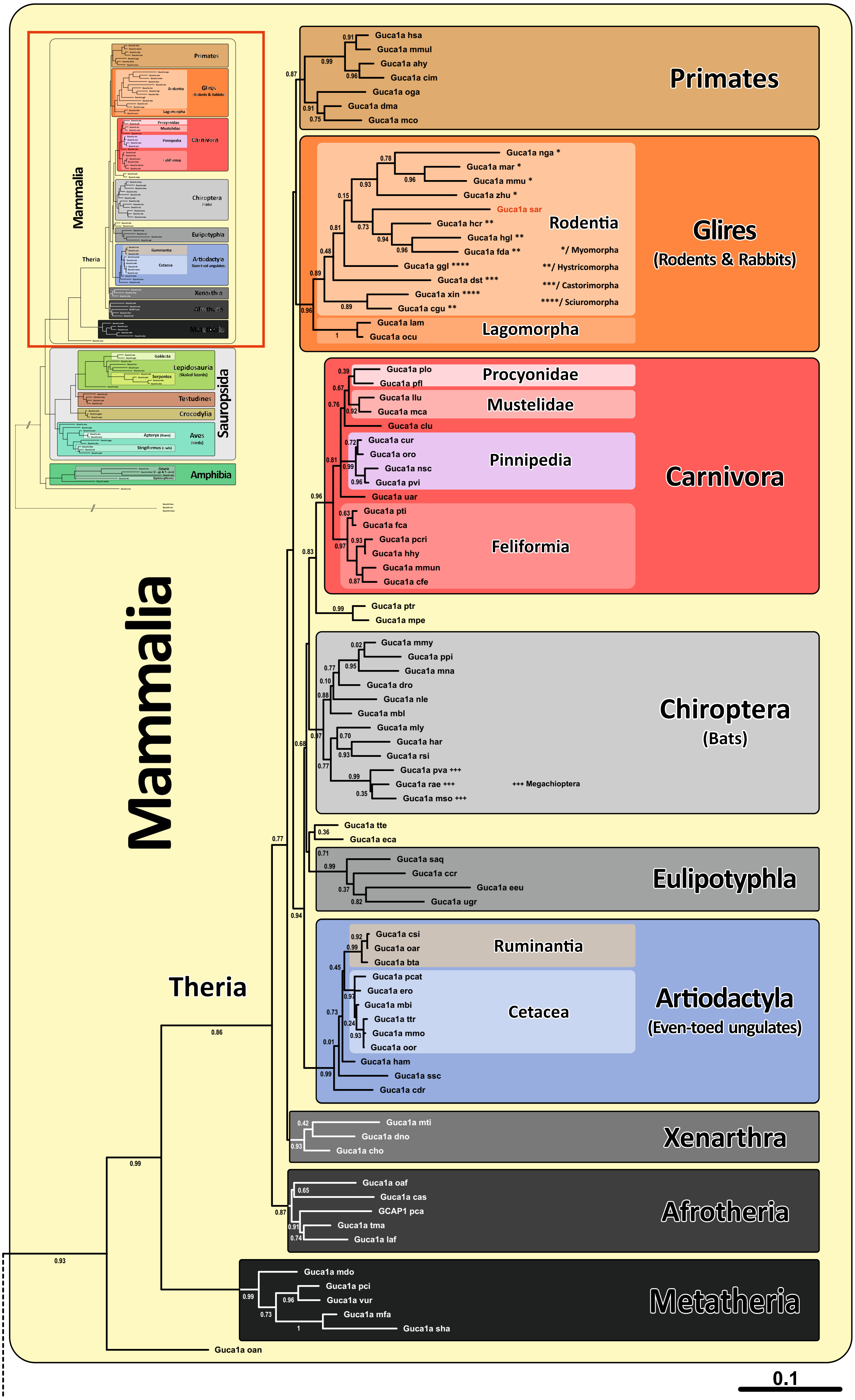

Figure S1: Mammalian *Guca1a* phylogenetic tree. Phylogeny was calculated based on the nucleotide alignment of 117 *guca1a* sequences from major vertebrate taxa. Key mammalian clades, orders and suborders are highlighted in colors. Species abbreviations can be found in supplementary table S1. Branch support values are given. Note that branch support values of less than 0.5 indicate the increasing likelihood of an alternate branch topology. The red highlighted sequence in the rodent branch represents the common shrew *Sorex araneus* sequence that counterintuitively groups into the rodent branch while generally belonging to the order of *Eulipotyphla*. The tree was rooted using *Guca1b* sequences from human, mouse and dog as outgroups.

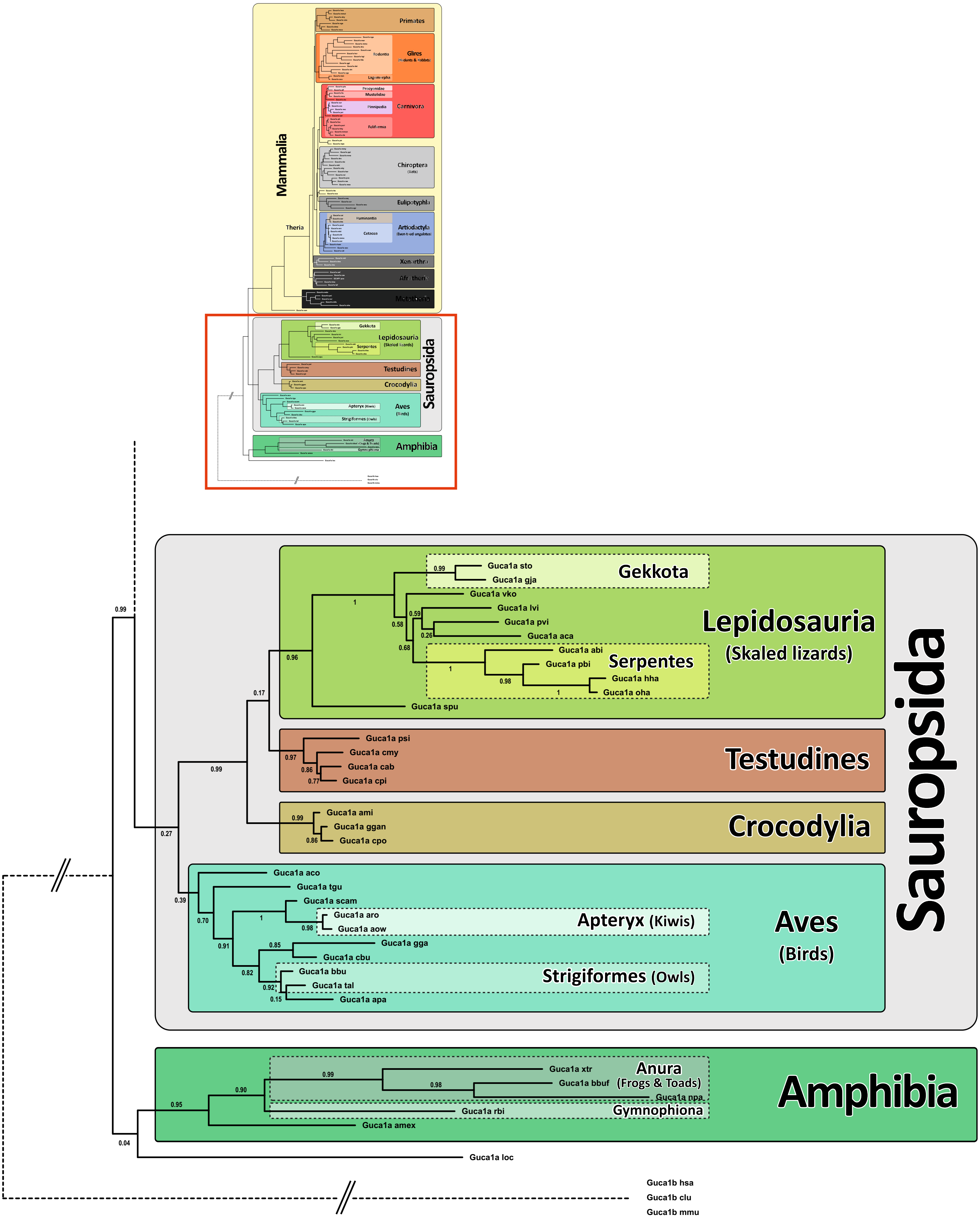

Figure S2: Sauropsida and Amphibia *Guca1a* phylogeny. Phylogeny was calculated based on the nucleotide alignment of 117 *guca1a* sequences from major vertebrate taxa. Key clades, orders and suborders are highlighted in colors. Species abbreviations can be found in supplementary table S1. Branch support values are given. Note that branch support values of less than 0.5 indicate the increasing likelihood of an alternate branch topology.

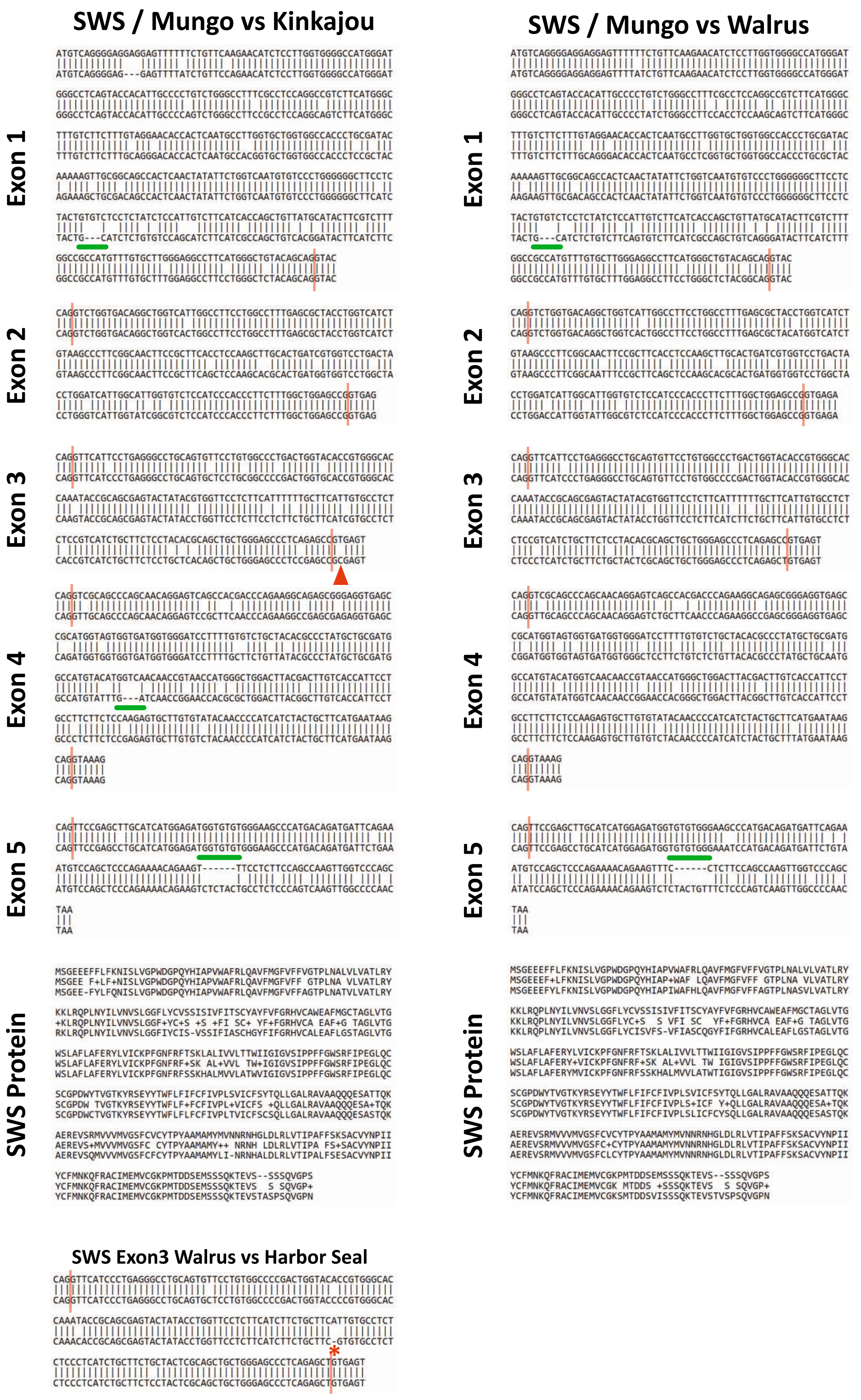

Figure S3: *Opsin1sw1* sequence alignments. Species used for alignment are indicated. Transparent red lines indicate intron/exon boundaries. Horizontal green lines indicate in frame deletions/insertions. The red triangle shows a possible mutation affecting the splice consensus sequence. However, we have seen multiple T – C exchanges which do not affect accurate splicing. A correct splice consensus could be generated by RNA editing. Note that no obvious mutation is detected in the *opsin1sw1* ORF in the walrus *Odobenus rosmarus*. For the harbor seal *Phoca vitulina* a single mutation in exon 3 is apparent (red asterisks).

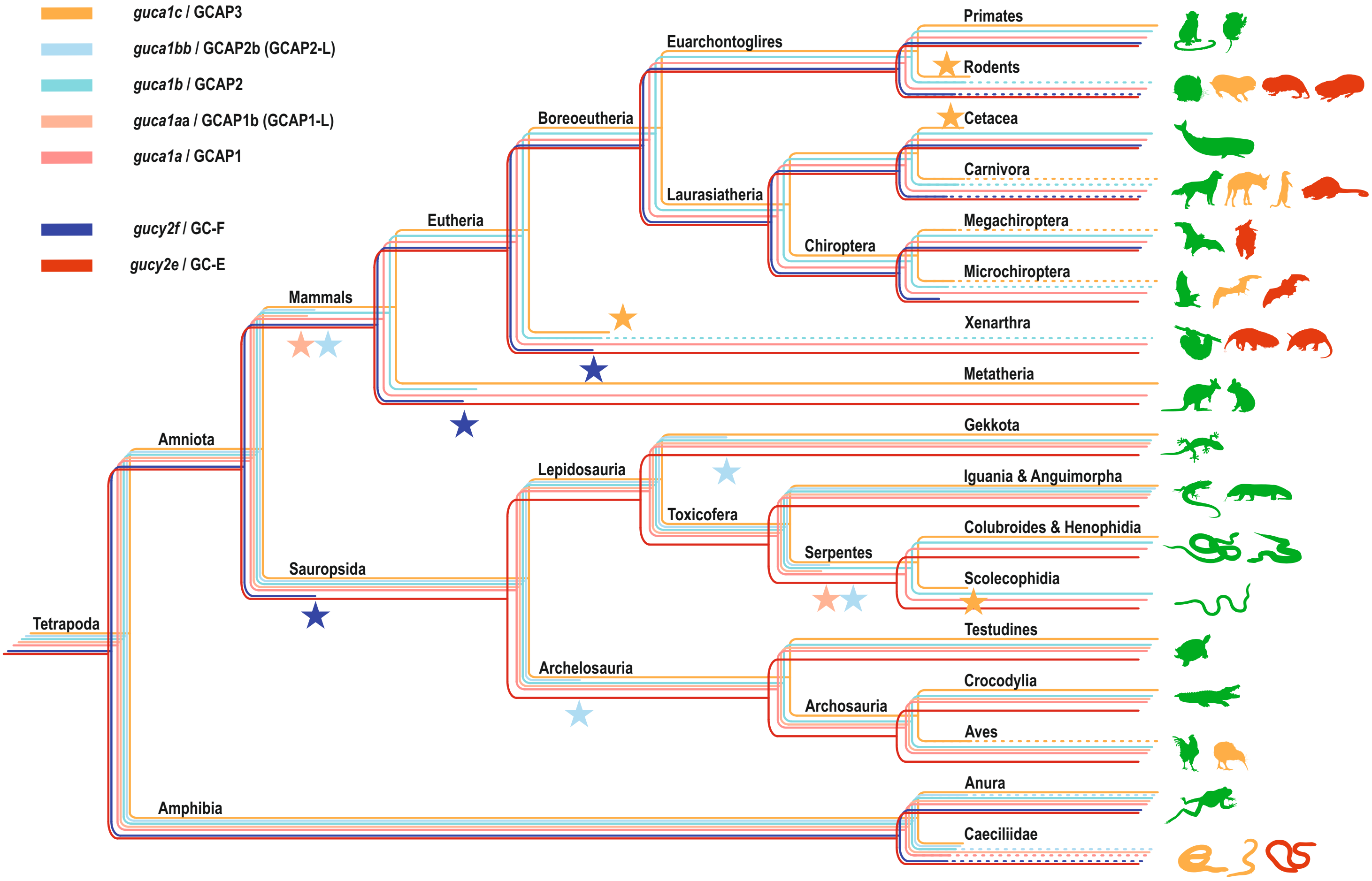

Figure S4: Evolution of guanylyl cyclase and guanylyl cyclase activating proteins in tetrapods. Cladograms for both visual guanylyl cyclases and for all the five GCAPs are given and the major vertebrate clades and families are labelled. The colour code for each gene/protein is indicated. While pseudogenizations/gene losses effecting an entire clade are shown by colour coded stars (the colour corresponds to the effected gene/protein), dotted lines indicate the selective inactivation of a given gene among the different spices of the clade. Green silhouettes on the right represent species having preserved the full gene content typical for this order/family, whereas orange silhouettes indicate species in which one or more genes are absent/inactivated within the clade and species highlighted by red silhouettes have reduced the gene content to the minimal 1:1 requirement. Note that the tree is a cladogram and not drawn to scale. For simplicity, spectral sensitivities are not indicated and some clades have been omitted.
